# Grey matter volume associations with personality functioning in a clinical cohort of female youths

**DOI:** 10.1016/j.nicl.2026.103988

**Published:** 2026-03-28

**Authors:** Madelyn Thomson, Marialuisa Cavelti, Ines Mürner-Lavanchy, Silvano Sele, Niklas Bürgi, Nora Seiffert, Franz Moggi, Roland Wiest, Julian Koenig, Michael Kaess

**Affiliations:** a University Hospital of Child and Adolescent Psychiatry and Psychotherapy, University of Bern, Bern, Switzerland; b Graduate School for Health Sciences, University of Bern, Switzerland; c Faculty of Psychology, University of Basel, Switzerland; d Department of Economics, University of Zurich, Switzerland; e Max Planck Institute for Biological Cybernetics, Tübingen, Germany; f Translational Research Center, University Hospital for Psychiatry and Psychotherapy, University of Bern, Switzerland; g University Institute of Diagnostic and Interventional Neuroradiology, Inselspital, Bern University Hospital, University of Bern, Bern, Switzerland; h Translational Imaging Center (TIC), Swiss Institute for Translational and Entrepreneurial Medicine, Bern, Switzerland; i University of Cologne, Faculty of Medicine and University Hospital Cologne, Department of Child and Adolescent Psychiatry, Psychosomatics and Psychotherapy, Cologne, Germany; j Department of Child and Adolescent Psychiatry, Centre for Psychosocial Medicine, University of Heidelberg, Heidelberg, Germany

**Keywords:** Personality disorder, Neuroimaging, Criterion A, Adolescents, AMPD, Dimensional

## Abstract

Borderline Personality Disorder (BPD) is a severe mental illness, although its neurobiological underpinnings remain largely unknown. Structural Magnetic Resonance Imaging (sMRI) in adolescents can offer insights into potential biomarkers to help advance early detection and targeted intervention. However, previous findings have been mixed, possibly due to clinical heterogeneity that may be better captured using a dimensional approach to personality functioning (PF). The current study explored grey matter volume (GMV) in youth with varying degrees of BPD pathology, and associations with dimensional PF. *N* = 93 females (14–21 years) comprising three groups (full-threshold BPD, sub-threshold BPD, and healthy controls) underwent sMRI and were assessed with the Semi-Structured Interview for Personality Functioning DSM-5 (STiP-5.1). Groups were combined to reflect dimensional personality pathology. Multiple linear regression analyses were conducted to determine associations between the STiP-5.1 total score, and each of its four elements with: (i) total GMV, (ii) GMV in individual brain regions defined by the Desikan-Killiany-Tourville atlas, (iii) selected regions of interest (ROIs). All analyses were statistically non-significant: STiP-5.1 total and total GMV (*p* = 0.61); STiP-5.1 total and individual brain regions (all corrected *p* values ≥0.82); STiP-5.1 total and ROIs (all corrected *p* values ≥0.91). Results were non-significant for each element, and a validity check using BPD criteria confirmed STiP-5.1 findings. We found no evidence of an association of dimensionally assessed PF with GMV in young females. The pursuit of clinical research efforts on other potential biomarkers using dimensional conceptualisations of PF may represent worthy endeavours.

## Highlights

• Structural MRI Borderline Personality Disorder (BPD) studies have mixed findings.
• Dimensional personality functioning (PF) was explored as an alternative in relation to grey matter volume (GMV).
• No evidence of an association between PF and GMV in female youths.
• Findings were confirmed when using BPD criteria as the predictor.
• Future studies should explore other potential biomarkers using PF.

## Graphical abstract

**Figure:**
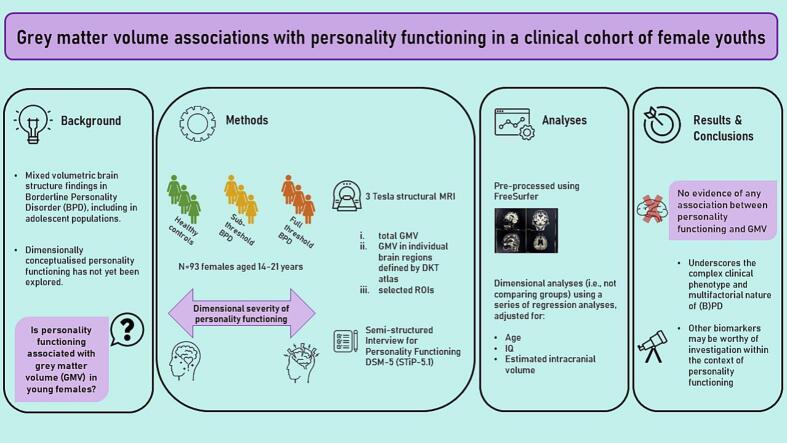

## 1 Introduction

Personality disorders (PD) are severe mental illnesses that incur substantial distress for patients, and high burden to society, with an estimated worldwide pooled prevalence of 7.8% (Winsper et al., 2020). Borderline Personality Disorder (BPD) is among the most commonly diagnosed PD, with recent evidence suggesting prevalence rates of up to 22% in clinical settings (Leichsenring et al., 2024), and is posited to represent core features of PD severity more broadly (Sharp et al., 2015). BPD typically first emerges during the developmentally vulnerable period of youth, which can have long lasting effects into adulthood (Sharp and Wall, 2018, Videler et al., 2019). Despite a remarkable increase in research on PD over the last 20 years (BPD in particular), the aetiology remains largely unknown (Hooley and Masland, 2017). While BPD has long been suggested to develop as a result of complex interactions between underlying (neuro)biological vulnerability and the environment (Linehan, 1993), the search for specific biomarkers remains elusive, and therefore, many theoretical models remain tentative (Brunner et al., 2015, Goodman et al., 2013). Given the clinical complexity of BPD, the lack of a comprehensive understanding of its neurobiological foundations ultimately hinders efforts at informing effective interventions for this frequently disabling disorder (Ruocco and Carcone, 2016). Indeed, BPD can be fatal, with high rates of suicide (up to 10%) (Leichsenring et al., 2024). Furthermore, even for those who experience some attenuation of symptoms over time, functional difficulties may persist for decades (Winograd et al., 2008, Winsper et al., 2015). Therefore, it is crucial to identify biomarkers that may help to elucidate PD phenotypes, enhance early detection (and possibly prevention), facilitate the development of targeted treatment, and improve the prediction of treatment response (Singh and Rose, 2009).

One promising biomarker in BPD research is brain structure, with most research focusing on grey matter volume (GMV) using structural Magnetic Resonance Imaging (sMRI). Previous studies in adults on GMV in BPD patients have produced inconsistent findings. Some have found reduced GMV in the hippocampus and amygdala compared to healthy controls (HC) (Ruocco et al., 2013, Schulze et al., 2016), others found no evidence of volume reductions in these regions (Aguilar-Ortiz et al., 2018), and others have found increases in limbic structure volume (Sampedro et al., 2021). Nevertheless, systematic reviews and *meta*-analyses have broadly implicated brain alterations in fronto-limbic regions involved in emotion processing and regulatory control processes, including the hippocampus, amygdala, anterior cingulate cortex (ACC), orbitofrontal cortex (OFC), and the dorsolateral prefrontal cortex (DLPFC) (Chan et al., 2020, Friedel et al., 2018, Krause-Utz et al., 2014, Nunes et al., 2009, O’Neill and Frodl, 2012, Perez-Rodriguez et al., 2018, Ruocco et al., 2012, Ruocco and Carcone, 2016, Schulze et al., 2016). In contrast, there exist considerably fewer sMRI studies in adolescent samples. Results have not reproduced volumetric differences reported in studies of adults with BPD (Goodman et al., 2014), and are frequently conflicting (for reviews of sMRI findings in adolescent BPD samples, see (Brunner et al., 2015, Courtney-Seidler et al., 2013, Goodman et al., 2013, Winsper et al., 2016)). Regardless, similar to adults, main study findings in adolescents with BPD also suggest some structural alterations in regions in the fronto-limbic network compared with HC (Yi et al., 2024).

One possible explanation for the largely mixed results regarding brain volume in (B)PD (especially in adolescent populations) is the heterogeneity of symptom presentation in categorically defined PD. Beyond within-category heterogeneity, the problem is further exacerbated by substantial diagnostic overlap and comorbidity between categorical PD (Widiger and Samuel, 2005), which appears to be even more diffuse in adolescent populations (Becker et al., 2000). A promising alternative avenue for research is the dimensional conceptualisation of PD as evidenced in section III Alternative Model of Personality Disorders (AMPD) of the *DSM-5*, and the *ICD-11.* Two key criteria of the AMPD include Criterion A, personality functioning (PF) impairment, and Criterion B, maladaptive personality traits, both of which must be met for a PD diagnosis. Criterion A in the AMPD is based on the Level of Personality Functioning Scale (LPFS) representing core deficits in self- and interpersonal functioning across a severity continuum that underlie all PD (Sharp and Wall, 2021). Criterion A is similarly required for a diagnosis in the *ICD-11*, while the maladaptive traits (Criterion B) are only optional specifiers (i.e., not required for diagnosis). Thus, Criterion A reflects the defining feature of PD in the newer diagnostic systems, also mirrored in findings suggesting that global indices of personality severity are a robust predictor of presence and prognosis of PD, as well as current and future adjustment (Christensen et al., 2019, Clark et al., 2018, Hopwood et al., 2011, Morey et al., 2013, Pincus et al., 2020). Criterion A’s dimensional nature aligns with emerging research frameworks such as the Research Domain Criteria (RDoC; (Insel et al., 2010, Stanley et al., 2018), which emphasises neurobiological studies of psychopathology across the lifespan using dimensional systems (Auerbach, 2022). RDoC holds promise for advancing neuroscience research and identifying phenotypes in PD (Koudys et al., 2019) by uncovering relevant (neuro)biomarkers through a dimensional approach (Abi-Dargham and Horga, 2016). Thus, investigating dimensional PF in the context of neural substrates may help to advance our understanding of personality pathology (Winsper et al., 2016).

Neurobiological research on self- and interpersonal functioning has mostly been conducted in the form of functional (f)MRI, or resting-state functional connectivity (rsFC; (Bertsch and Herpertz, 2021b, Herpertz et al., 2014, Herpertz et al., 2018, Ruocco and Carcone, 2016), using a range of proxy measures of self- and interpersonal processes, or measurement of related PD traits and symptoms. A wide variety of brain regions has been implicated in the study of self- and interpersonal functioning, which extend beyond the frequently studied ROIs in BPD. However, only one fMRI study has specifically examined associations with PF (based on the LPFS) (Traynor et al., 2021). Using group comparisons in an adult sample, they found that severity of PF impairment was associated with stronger intra-limbic rsFC. However, despite these broader findings in self- and interpersonal functioning processes (mostly using fMRI), to date – and to the best of our knowledge – no study has examined *structural* alterations in adolescents using direct assessment of self- and interpersonal functioning as proposed in Criterion A of the AMPD.

Therefore, the aim of the current study was to explore neural substrates in the form of brain structure (GMV) in youth with varying degrees of BPD pathology, and their associations with dimensionally conceptualised PF. Examining personality pathology in youth may be particularly useful for sMRI research because this developmental period coincides with the onset of PDs (Sharp and Wall, 2018), and early-stage personality pathology is less impacted by confounding factors such as duration of illness, drug abuse, or prolonged medication use (Brunner et al., 2010, Chanen et al., 2008). Relatedly, Criterion A is suggested to be better able to detect early signs of personality pathology in young people than categorical PD diagnoses (Weekers et al., 2020), and is posited to account for the emergence of PD in adolescence, reflecting the coalescence in the development of self- and interpersonal functioning – an important developmental task during this period (Sharp, 2020). Therefore, studying PF in association with brain architecture in adolescents may provide valuable insights into the aetiopathogenesis of PD, and elucidate important biomarkers than can inform early detection and intervention. Given the broad spectrum of brain regions potentially implicated in PF, we took an explorative approach. In addition, while neuroimaging findings in adolescents have been largely mixed, sMRI research in BPD has fronto-limbic regions as key targets (Cruz-Ausejo et al., 2025, Davies et al., 2020). Accordingly, we focused on the fronto-limbic regions of interest (ROI) in particular (i.e. amygdala, hippocampus, DLPFC, ACC, and OFC), in addition to individual brain regions as defined by the Desikan-Killiany-Tourville [DKT] atlas (Klein and Tourville, 2012), in relation to PF among adolescents with and without BPD features. Additionally, we also used BPD criteria (in place of PF) as a validity check for the results.

## 2 Methods and materials

### 2.1 Participants and procedures

Clinical participants were recruited through inpatient and outpatient facilities from the University Psychiatry Services in Bern, Switzerland. Those aged 14–17 years were recruited through the child and adolescent clinics, participants aged 18–21 years were recruited through adult psychiatry clinics, and HCs from the general population in the region of Bern, Switzerland. We sought to recruit participants with varying degrees of personality pathology, to obtain a representative sample of participants using a dimensional approach. Therefore, we included 3 groups: full-threshold BPD, sub-threshold BPD, and (age and sex matched) HC, all assessed for BPD criteria using structured clinical interviews. Inclusion criteria for all subgroups were: aged between 14–21 years; and exclusion criteria were: insufficient German language skills, contraindications for MRI, acute psychotic symptoms or suicidality, schizophrenia, bipolar affective disorder type 1, alcohol or substance dependence, inability to provide informed consent and/or adhere to the study procedure. For the full-threshold BPD group, additional inclusion criteria included having met 5–9 symptoms of *DSM-4* BPD criteria, and for the sub-threshold BPD group, additional inclusion criteria included having met 1–4 symptoms of *DSM-4* BPD criteria. For the HC subgroup, additional exclusion criteria included meeting any current and/or lifetime psychiatric disorder/treatment, and meeting any *DSM-4* BPD criteria.

Written informed consent was obtained from all participants. The study comprised two appointments for all participants. Psychopathology assessments were conducted by clinically trained psychologists or trained doctoral students. Participants were then asked to complete the online questionnaires, and at the second appointment underwent MRI. Participants were reimbursed with 20CHF for the psychiatric assessments, and 20CHF for participation in the MRI (total 40CHF). The study was approved by the local Ethics Committee (Ethics ID: 2019-01525).

### 2.2 Neuroimaging – MRI scanning

MRI acquisition was performed using a 3T Siemens MAGNETOM Prisma scanner at the Translational Imaging Center of the Swiss Institute for Translational and Entrepreneurial Medicine (SITEM) in Bern, Switzerland, in collaboration with the Institute of Diagnostic and Interventional Neuroradiology, Bern. No contrast agent was administered. The 64-channel head coil was used for all MR images. High-resolution T1-weighted MR images were obtained using a 3D magnetization-prepared rapid two-gradient-echo with 2 inversion times (MP2RAGE) sequence. The optimized acquisition parameters were as follows: 176 sagittal slices, 256 × 224 matrix (with a non-cubic field of view (FOV) of 256 × 224 mm^2^, yielding a nominal isotropic resolution of 1 mm^3^), 5000 ms repetition time (TR), 2.98 ms echo time (TE), 700 ms and 2500 ms inversion time (TI), flip angle 4° and 5°, GRAPPA acceleration factor 3 and an 8.22-minute total acquisition time.

### 2.3 Image processing

FreeSurfer version 6.0 (Reuter et al., 2010) was used to automatically segment T1-weighted images using the DKT atlas (Klein and Tourville, 2012), which is automatically classified. The volume-based segmentation process is performed in three main steps including: (1) labelling of brain structures by registration to MNI305 space; (2) segmentation based on a subject-independent probabilistic atlas and subject-specific measured values; and (3) classification of each voxel to a label. After segmentation, all images underwent visual quality checks by trained researchers. Nine (9.6%) participants had manual corrections applied, and the recon-all process (a series of automated and semi-automated processes that aim to reconstruct the cortical and subcortical structures from the structural MRI data) was re-run, and further visual inspections were conducted. Details of FreeSurfer segmentation are described elsewhere (Fischl et al., 2002, Fischl et al., 2004). The volumes (mm^3^) of regions which are labelled as a particular structure are then calculated by FreeSurfer for further use in statistical analyses. The full list of individual brain regions examined in the analyses can be found in Table S1 of the Supplementary Materials.

To represent the ROIs, some regions automatically labelled by FreeSurfer (in the DKT atlas) were combined as follows: (1) DLPFC: combined volumes of the superior frontal, rostral middle frontal, and caudal middle frontal regions; (2) ACC: combined volumes of the rostral anterior cingulate, and the caudal anterior cingulate cortexes; (3) OFC: combined volumes of the pars orbitalis, lateral orbitofrontal, and medial orbitofrontal regions. The combination of regions was conducted separately for left and right hemispheres.

### 2.4 Measures

*Sociodemographic data* were collected using a standardised set of interview questions. *Personality Functioning* was measured using the Semi-Structured Interview for Personality Functioning DSM-5 (STiP-5.1; Hutsebaut et al., 2017) which assesses the severity of impairments in PF reflected in the LPFS of the AMPD. Self- and interpersonal functioning domains are further broken down into four elements (identity, self-direction, empathy, and intimacy). For each, the level of functioning can range from *little to no impairment* (0) to *extreme impairment* (4) in functioning. *BPD criteria* were measured using the Borderline-Module of the Structured Clinical Interview for DSM-IV Axis II Disorders (SCID-II; Fydrich et al., 1997). *Comorbid psychiatric diagnoses* were assessed using The Mini-International Neuropsychiatric Interview for Children and Adolescents, (MINI-KID; Sheehan et al., 2010). To establish the *absence of psychiatric disorders for HC* for eligibility, the Structured Clinical Interview for *DSM-IV-TR*, non-patient edition (SCID-N/P; First et al., 2002) was used. *Intelligence Quotient (IQ)* was assessed using the Wechsler Intelligence Scale for Children – Fifth Edition (WISC-V; Wechsler and Petermann, 2017) and the Wechsler Adult Intelligence Scale – Fourth Edition (WAIS-IV; Wechsler and Petermann, 2012). An *MRI safety screening* questionnaire was applied before participants entered the scanner, to confirm that they did not meet criteria for MRI contraindication. *Depression severity* was assessed using the Beck Depression Inventory II (BDI-II; Beck et al., 1996). *Childhood trauma* was measured using the Childhood Trauma Questionnaire (CTQ; Bernstein et al., 1994). The Edinburgh Handedness Inventory (Oldfield, 1971) was used to assess *handedness*. For further detail regarding assessments, please refer to Text S1 in Supplementary Materials.

### 2.5 Statistical analyses

A series of multiple linear regression analyses were conducted to determine the association between the STiP-5.1 (total score and the four element scores) as continuous variables across participants (i.e., HC, sub-threshold, full-threshold all pooled together), and i) whole brain GMV, ii) individual brain regions (as defined by the DKT), and iii) specified ROIs as outcome variables in participants. We conducted the analyses over the following steps:

1. A regression analysis was conducted to test the overall association between the STiP-5.1 total and total GMV.
2. We conducted the same analysis (STiP-5.1 total score as the predictor) using each individual brain region as the outcome variable.
3. Both of these analyses (i.e., using total GMV and individual regions as outcome variables) were additionally conducted with each STiP-5.1 element (identity, self-direction, empathy, intimacy) as a separate predictor for four added models.
4. We conducted the same regression analyses (STiP-5.1 total, as well as individual elements as separate predictors in separate models) on the selected ROIs (ACC, OFC, hippocampus, amygdala, DLPFC).
5. Finally, we conducted a validity check of the results using number of fulfilled BPD criteria as the predictor variable (instead of STiP-5.1 total or elements), using the same regression analyses as above (i.e., total GMV, individual regions, and ROIs as outcome variables in separate models).

For consistency, non-parametric permutation was applied to all tests, using the Permuco package in R. The default permutation of Freedman and Lane (Freedman and Lane, 1983, Frossard and Renaud, 2021, Winkler et al., 2014) with 5,000 synchronised permutations over all the brain regions – which implicitly accounts for the dependence among them – was applied to all analyses. For regression analyses across individual brain regions (i.e., not for total GMV only), *p* values were corrected to control for multiple testing issues. For this purpose, we used a maxT correction, which creates a distribution of the null hypothesis using the maximum t-value of each brain region, then compares the observed t-value with non-permutated data (Frossard and Renaud, 2021, Winkler et al., 2014). Additionally, joint tests using Fisher’s method (Fisher, 1932, Winkler et al., 2016) were conducted for the STiP-5.1 total, and for each of the four elements, over all brain regions to determine any overall evidence for an effect. The alpha level was set at *α* = 0.05. All statistical analyses were performed using R and RStudio Statistical software (Version 4.3.2; (R Core Team, 2023)).

### 2.6 Model selection

The regression models used in all analyses were controlled for age, estimated intracranial volume (ICV), and intelligence quotient (IQ). We additionally tested for a STiP-5.1 × age interaction effect, which was statistically non-significant, and therefore, a simpler final model (i.e., model without the interaction term) was chosen. For further information regarding covariates, including various model iterations for transparency purposes, please refer to Text S2 and Table S2 in Supplementary Materials.

## 3 Results

### 3.1 Participants

Ninety-four participants underwent MRI. However, one participant (from the full-threshold BPD group) did not have data for IQ and was therefore excluded from the final analyses, leaving a final total of *N* = 93 (*M*_age_ = 16.21 years, *SD* = 1.47, range = 14–21 years). All participants were female. In total, 32 participants (34%) met diagnostic threshold for Criterion A in the *DSM-5* AMPD. See Table 1 for descriptive statistics for the sample by subgroup.

**Table 1:** Sample description by subgroup (N = 93).

		Healthy controls (HC) *n* = 30	Sub-threshold BPD *n* = 31	Full-threshold BPD *n* = 32
*Sociodemographic variables*				
Age (years) M (SD)		16.10 (1.18)	16.10 (1.56)	16.44 (1.63)
Handedness n right, %		26 (86.7%)	24 (77.4%)	28 (87.5%)
IQ (M, SD)		108.07 (10.96)	97.42 (12.28)	99.34 (12.10)
Education level (currently completing or graduated)^a^				
	Primary School (ISCED level 1; at least 6 school years) n, (%)	0 (0%)	0 (0%)	1 (3.13%)
	Lower secondary School (ISCED level 2; 9–10 school years) n, (%)	4 (13.33%)	22 (70.96%)	26 (81.25%)
	Upper secondary Education (ISCED level 3; 12–13 school years) n, (%)	26 (86.66%)	6 (19.35%)	4 (12.5%)
	Other	0 (0%)	3 (9.68%)	1 (3.13%)
Living situation				
	Both parents n, (%)	27 (90%)	11 (35.48%)	11 (34.38%)
	Mother only n, (%)	1 (3.33%)	4 (12.90%)	5 (15.63%)
	Father only n, (%)	0 (0%)	0 (0%)	0 (0%)
	Other (e.g. step parents, etc.) n, (%)	2 (6.67%)	16 (51.61%)	16 (50%)

*Clinical variables*				

	Criterion A fulfilled n, (%)	0 (0%)	11 (35%)	21 (66%)
	STiP-5.1 total score (M, SD)	0.15 (0.13)	1.23 (0.69)	1.90 (0.64)
	STiP-5.1 Identity (M, SD)	0.21 (0.20)	1.98 (1.12)	2.80 (0.96)
	STiP-5.1 Self-direction (M, SD)	0.11 (0.18)	1.25 (0.81)	1.90 (0.72)
	STiP-5.1 Empathy (M, SD)	0.16 (0.23)	0.82 (0.74)	1.26 (0.66)
	STiP-5.1 Intimacy (M, SD)	0.10 (0.18)	0.87 (0.74)	1.73 (1.08)
	Number of BPD criteria fulfilled (M, SD)	−	2.90 (1.27)	6.78 (1.39)
	BDI (depressivity) (M, SD)	5.30 (3.50)	32.74 (16.28)	39.55 (13.78)
	CTQ (trauma) (M, SD)	30.73 (6.71)	48.23 (15.33)	58.91 (18.63)
	Number of MINI diagnoses (M, SD)	0 (0)	3.16 (2.86)	4.0 (2.76)
	Any F1 disorder (substance related disorders, e.g. drugs or alcohol) n, (%)	0 (0%)	8 (25.81%)	20 (62.5%)
	Any F2 disorder (psychotic disorders) n, (%)	0 (0%)	0 (0%)	0 (0%)
	Any F3 disorder (affective disorders) n, (%)	0 (0%)	26 (83.87%)	32 (100%)
	Any F4 disorder (anxiety, dissociative, stress-related, somatoform and other nonpsychotic mental disorders) n, (%)	0 (0%)	24 (77.42%)	29 (90.62%)
	Any F5 disorder (behavioural syndromes associated with physiological disturbances and physical factors, e.g. eating disorders, sleep disorders, sexual dysfunction) n, (%)	0 (0%)	6 (19.35%)	5 (15.63%)
	Any F8 disorder (pervasive and specific developmental disorders, e.g. disorders of speech and language, scholastic skills) n, (%)	0 (0%)	0 (0%)	3 (9.38%)
	Any F9 disorder (Behavioural and emotional disorders with onset usually occurring in childhood and adolescence, e.g. ADHD, conduct disorder) n, (%)	0 (0%)	5 (16.13%)	8 (25%)
	Psychotropic medication n yes, (%)	0 (0%)	11 (35.48%)	17 (53.13%)

*Notes:* STiP-5.1 = Semi-Structured Interview for Personality Functioning DSM-5, BDI = Beck Depression Inventory, CTQ = Childhood Trauma Questionnaire. F6 diagnoses (personality disorders) were not made using the MINI, and only the Borderline module of the SCID-II was conducted, therefore, the frequency of F6 (disorders of personality and behaviour) cannot be reported. Similarly, F7 diagnoses (mental retardation) cannot be assessed by the MINI-KID, and therefore, the frequency of F7 diagnoses cannot be reported.

a Education levels are based on International Standard Classification of Education (ISCED).

### 3.2 Associations between the STiP-5.1 and total GMV

The association between the STiP-5.1 total and total GMV was statistically non-significant (uncorrected *p* = 0.61). When examined individually using each of the four elements as a separate predictor variable, all regression analyses were non-significant for total GMV (all four *p* values ≥0.36). See Table 2 for further details of the main regression analysis results (i.e., STiP-5.1 total), and Tables S3–S6 in Supplementary Materials for results of regression analyses for each element with total GMV.

**Table 2:** Regression analysis results of STiP-5.1 total scores on total GMV (N = 93).

Predictors	Estimates, [95% CI]	p value
(Intercept)	658271.73 [648417.47, 668125.98]	<0.01
STiP-5.1 total	−1798.75 [−8857.65, 5260.15]	0.61
Age	−285.69 [−4427.29, 3855.91]	0.89
ICV	43619.29 [37286.11, 49952.48]	<0.01
IQ	204.18 [−304.41, 712.76]	0.43
Num.Obs.	93	−–
R2	0.717	−–

Shown are parameter estimates (coefficients) with 95% confidence intervals.

*Notes:* CI = confidence intervals; STiP-5.1=Semi-structured Interview for Personality Functioning DSM-5; ICV = Estimated Intracranial Volume; IQ = Intelligence Quotient.

### 3.3 Associations between the STiP-5.1 and individual brain regions

When examining the association between the STiP-5.1 total and each individual brain region, all regression analyses were found to be statistically non-significant when *p* values were corrected (all corrected *p* values ≥0.82). Joint tests for the STiP-5.1 total revealed no evidence for an overall effect (*p* = 0.45). The lowest uncorrected *p*-value was found for the association between the STiP-5.1 total and the right putamen (uncorrected *p* value = 0.03). See Table 3 for results of all regression analyses for the STiP-5.1 total and each individual brain region. Additionally, when broken down into each of the four elements, joint tests revealed no evidence for an overall effect for any element (Identity: *p* = 0.40; Self-direction: *p* = 0.51; Empathy: *p* = 0.74; Intimacy: *p* = 0.42). Further, none of the regression analyses were found to be statistically significant when *p* values were corrected for multiple testing (Identity: all corrected *p* values ≥0.76; Self-direction: all corrected *p* values ≥0.87; Empathy: all corrected *p* values ≥0.66; Intimacy: all corrected *p* values ≥0.33). The lowest uncorrected *p*-values for each element were as follows: Identity: Right caudal middle frontal gyrus (uncorrected *p* value = 0.02), right putamen (uncorrected *p* value = 0.04), and right pallidum (uncorrected *p* value = 0.04); Self-direction: Right putamen (uncorrected *p* value = 0.03), right pallidum (uncorrected *p* value = 0.04); Empathy: Left caudal middle frontal gyrus (uncorrected *p* value = 0.02), lateral occipital gyrus (uncorrected *p* value = 0.02); and Intimacy: Right lateral occipital gyrus (uncorrected *p* value = 0.04), right posterior cingulate cortex (uncorrected *p* value = 0.03), right precuneus (uncorrected *p* value < 0.01), left putamen (uncorrected *p* value = 0.04), right putamen (uncorrected *p* value = 0.02), right accumbens-area (uncorrected *p* value = 0.02). See Tables S7–S10 in Supplementary Materials for results of each regression analysis for individual brain regions, by element.

**Table 3:** Regression analysis results of STiP-5.1 total scores on individual brain region GMV (N = 93).

	(Intercept)	STiP-5.1 total	Age	ICV	IQ	PvalCor
Lh-banks superior temporal sulcus	2518.01 [2372.05, 2663.98] < 0.01	−31.98 [−136.55, 72.58] 0.54	−17.53 [−78.88, 43.81] 0.57	257.83 [164.02, 351.64] < 0.01	−4.45 [−11.98, 3.08] 0.24	1.00
Lh-caudal anterior cingulate cortex	1846.39 [1682.47, 2010.32] < 0.01	16.20 [−101.23, 133.62] 0.78	14.61 [−54.29, 83.50] 0.67	177.81 [72.46, 283.16] < 0.01	0.17 [−8.29, 8.63] 0.97	1.00
Lh-caudal middle frontal gyrus	7032.20 [6678.59, 7385.81] < 0.01	−210.88 [−464.18, 42.43] 0.10	−180.19 [−328.80, −31.57] 0.02	229.49 [2.23, 456.75] 0.05	−3.03 [−21.28, 15.22] 0.74	1.00
Lh-cuneus	2908.05 [2720.22, 3095.89] < 0.01	−61.14 [−195.69, 73.41] 0.37	4.76 [−74.19, 83.70] 0.90	223.27 [102.55, 343.98] < 0.01	0.26 [−9.43, 9.96] 0.96	1.00
Lh-entorhinal	2028.04 [1871.88, 2184.19] < 0.01	−80.77 [−192.63, 31.08] 0.15	1.63 [−64.00, 67.26] 0.96	61.80 [−38.55, 162.16] 0.22	−0.09 [−8.15, 7.97] 0.98	1.00
Lh-fusiform	9507.82 [9179.97, 9835.67] < 0.01	−107.16 [−342.01, 127.69] 0.37	92.66 [−45.13, 230.45] 0.18	741.87 [531.17, 952.58] < 0.01	11.62 [−5.30, 28.54] 0.18	1.00
Lh-inferior parietal lobule	12385.81 [11869.19, 12902.43] < 0.01	−4.15 [−374.22, 365.92] 0.98	9.39 [−207.74, 226.52] 0.93	1007.04 [675.02, 1339.07] < 0.01	2.67 [−24.00, 29.33] 0.84	1.00
Lh- inferior temporal gyrus	11494.61 [10946.21, 12043.00] < 0.01	–233.62 [−626.46, 159.21] 0.24	−53.85 [−284.33, 176.64] 0.64	797.68 [445.23, 1150.12] < 0.01	1.16 [−27.14, 29.47] 0.94	1.00
Lh-istmus cingulate cortex	2605.41 [2515.00, 2695.81] < 0.01	−15.24 [−80.00, 49.51] 0.64	−21.25 [−59.25, 16.74] 0.27	176.87 [118.77, 234.97] < 0.01	2.87 [−1.80, 7.54] 0.22	1.00
Lh-lateral occipital gyrus	11530.31 [11095.67, 11964.96] < 0.01	40.16 [−271.19, 351.51] 0.80	27.17 [−155.51, 209.85] 0.77	716.76 [437.42, 996.10] < 0.01	−4.95 [−27.38, 17.48] 0.66	1.00
Lh-lateral orbitofrontal	7953.98 [7652.82, 8255.15] < 0.01	−38.39 [−254.12, 177.34] 0.72	24.25 [−102.33, 150.82] 0.70	493.47 [299.91, 687.02] < 0.01	16.30 [0.76, 31.84] 0.04	1.00
Lh-Lingual	6054.41 [5757.19, 6351.63] < 0.01	−119.53 [–332.44, 93.38] 0.27	−77.01 [−201.92, 47.91] 0.22	336.24 [145.22, 527.26] < 0.01	1.70 [−13.64, 17.04] 0.83	1.00
Lh-medial orbitofrontal	5618.91 [5395.42, 5842.41] < 0.01	64.99 [−95.10, 225.08] 0.42	−39.28 [−133.21, 54.65] 0.41	461.44 [317.80, 605.07] < 0.01	6.29 [−5.24, 17.82] 0.28	1.00
Lh-middle temporal gyrus	11375.94 [10891.56, 11860.31] < 0.01	−6.77 [−353.74, 340.20] 0.97	−109.65 [−313.23, 93.92] 0.29	906.69 [595.39, 1217.99] < 0.01	−14.35 [−39.35, 10.65] 0.26	1.00
Lh-parahippocampal	2130.87 [2038.09, 2223.65] < 0.01	−19.28 [−85.74, 47.18] 0.57	−11.95 [−50.94, 27.05] 0.54	52.53 [−7.10, 112.16] 0.08	5.95 [1.17, 10.74] 0.02	1.00
Lh-paracentral	3464.80 [3324.02, 3605.58] < 0.01	13.15 [−87.69, 113.99] 0.80	7.03 [−52.14, 66.19] 0.81	214.37 [123.90, 304.85] < 0.01	3.73 [−3.54, 10.99] 0.31	1.00
Lh-pars opercularis	4925.46 [4703.48, 5147.44] < 0.01	119.37 [−39.64, 278.38] 0.14	−109.17 [−202.47, −15.88] 0.02	402.20 [259.54, 544.86] < 0.01	3.34 [−8.12, 14.80] 0.56	1.00
Lh-pars orbitalis	2696.25 [2556.00, 2836.51] < 0.01	−31.42 [−131.89, 69.05] 0.54	11.60 [−47.35, 70.54] 0.70	221.15 [131.01, 311.29] < 0.01	4.15 [−3.09, 11.39] 0.26	1.00
Lh-pars triangularis	4067.20 [3859.80, 4274.60] < 0.01	−2.64 [−151.21, 145.93] 0.97	−2.69 [−89.86, 84.47] 0.95	393.26 [259.96, 526.55] < 0.01	5.51 [−5.20, 16.21] 0.31	1.00
Lh-pericalcarine	1849.30 [1711.08, 1987.52] < 0.01	−42.65 [−141.66, 56.36] 0.39	3.19 [−54.90, 61.29] 0.91	120.22 [31.39, 209.05] < 0.01	0.05 [−7.08, 7.19] 0.99	1.00
Lh-postcentral	9213.10 [8840.19, 9586.01] < 0.01	51.67 [−215.46, 318.79] 0.70	108.81 [−47.92, 265.54] 0.17	759.02 [519.36, 998.68] < 0.01	2.71 [−16.53, 21.96] 0.78	1.00
Lh-posterior cingulate cortex	3203.34 [3057.18, 3349.50] < 0.01	−17.55 [−122.25, 87.15] 0.74	30.00 [−31.43, 91.43] 0.33	216.08 [122.14, 310.02] < 0.01	6.04 [−1.50, 13.59] 0.12	1.00
Lh-precentral	13333.75 [12935.05, 13732.46] < 0.01	−113.65 [−399.26, 171.96] 0.43	−77.35 [−244.92, 90.23] 0.36	767.79 [511.55, 1024.04] < 0.01	0.95 [−19.63, 21.53] 0.93	1.00
Lh-precuneus	9597.23 [9277.88, 9916.57] < 0.01	47.69 [−181.06, 276.45] 0.68	101.79 [–32.42, 236.01] 0.14	784.59 [579.35, 989.83] < 0.01	10.01 [−6.47, 26.49] 0.23	1.00
Lh-rostral anterior cingulate cortex	2838.75 [2678.68, 2998.82] < 0.01	−110.55 [−225.21, 4.11] 0.06	57.32 [−9.95, 124.60] 0.09	210.90 [108.03, 313.77] < 0.01	4.28 [−3.98, 12.54] 0.31	0.97
Lh-rostral middle frontal gyrus	17498.48 [16958.74, 18038.23] < 0.01	−151.68 [−538.32, 234.95] 0.44	−109.83 [−336.68, 117.01] 0.34	1616.63 [1269.74, 1963.51] < 0.01	−3.04 [−30.89, 24.82] 0.83	1.00
Lh-superior frontal gyrus	24916.13 [24205.18, 25627.07] < 0.01	39.07 [−470.20, 548.34] 0.88	1.45 [−297.35, 300.25] 0.99	1858.02 [1401.10, 2314.93] < 0.01	24.20 [−12.49, 60.89] 0.19	1.00
Lh-superior parietal lobule	13104.31 [12565.60, 13643.01] < 0.01	–32.53 [−418.42, 353.36] 0.87	128.12 [−98.29, 354.53] 0.26	924.81 [578.59, 1271.02] < 0.01	0.26 [−27.54, 28.06] 0.99	1.00
Lh-superior temporal gyrus	12544.22 [12116.53, 12971.91] < 0.01	−44.64 [−351.01, 261.73] 0.77	27.58 [−152.17, 207.33] 0.76	887.11 [612.24, 1161.98] < 0.01	−3.51 [−25.58, 18.57] 0.75	1.00
Lh-supramarginal gyrus	11649.24 [11064.89, 12233.58] < 0.01	164.10 [−254.48, 582.69] 0.44	−2.93 [−248.53, 242.66] 0.98	1220.56 [845.01, 1596.11] < 0.01	11.22 [−18.94, 41.38] 0.46	1.00
Lh-frontal pole	1112.69 [1057.62, 1167.76] < 0.01	14.04 [−25.41, 53.49] 0.48	−19.60 [−42.75, 3.54] 0.10	24.21 [−11.19, 59.60] 0.18	0.98 [−1.86, 3.83] 0.49	1.00
Lh-temporal pole	2457.65 [2278.03, 2637.28] < 0.01	−56.45 [−185.12, 72.23] 0.39	35.09 [−40.41, 110.58] 0.36	33.74 [−81.71, 149.18] 0.56	2.08 [−7.19, 11.35] 0.66	1.00
Lh-transverse temporal	1079.47 [1017.76, 1141.18] < 0.01	−9.88 [−54.09, 34.32] 0.66	9.31 [−16.63, 35.24] 0.48	61.54 [21.88, 101.20] < 0.01	−0.74 [−3.92, 2.45] 0.65	1.00
Lh-insula	7242.71 [7029.45, 7455.96] < 0.01	−48.39 [−201.16, 104.37] 0.53	30.70 [−58.93, 120.33] 0.50	387.65 [250.59, 524.71] < 0.01	5.85 [−5.15, 16.86] 0.29	1.00
Rh-banks superior temporal sulcus	2369.44 [2253.02, 2485.85] < 0.01	−14.86 [−98.25, 68.52] 0.72	−15.15 [−64.08, 33.77] 0.54	174.81 [100.00, 249.63] < 0.01	−0.62 [−6.63, 5.39] 0.84	1.00
Rh-caudal anterior cingulate cortex	2066.83 [1911.49, 2222.17] < 0.01	−0.59 [−111.87, 110.68] 0.99	−14.87 [−80.16, 50.41] 0.65	233.42 [133.58, 333.25] < 0.01	1.06 [−6.96, 9.08] 0.79	1.00
Rh-caudal middle frontal gyrus	6881.69 [6551.33, 7212.05] < 0.01	−239.32 [−475.97, −2.67] 0.05	−34.76 [−173.61, 104.08] 0.62	488.56 [276.24, 700.88] < 0.01	11.47 [−5.58, 28.52] 0.18	0.94
Rh-cuneus	3178.83 [2981.00, 3376.65] < 0.01	−41.69 [−183.40, 100.01] 0.56	−16.06 [−99.20, 67.08] 0.70	171.45 [44.31, 298.59] < 0.01	−3.78 [−13.99, 6.43] 0.46	1.00
Rh-entorhinal	1874.54 [1741.50, 2007.58] < 0.01	−41.50 [−136.81, 53.80] 0.39	−27.29 [−83.20, 28.63] 0.33	85.77 [0.26, 171.27] 0.05	1.89 [−4.97, 8.76] 0.59	1.00
Rh-fusiform	9132.30 [8803.48, 9461.12] < 0.01	−142.63 [−378.18, 92.91] 0.23	−28.83 [−167.03, 109.36] 0.68	825.09 [613.77, 1036.42] < 0.01	−2.84 [−19.81, 14.13] 0.74	1.00
Rh-inferior parietal lobule	14832.63 [14258.53, 15406.73] < 0.01	246.10 [−165.14, 657.34] 0.24	81.35 [−159.93, 322.64] 0.50	1231.98 [863.02, 1600.94] < 0.01	26.19 [−3.44, 55.82] 0.08	1.00
Rh-inferior temporal gyrus	11174.34 [10731.58, 11617.09] < 0.01	–23.38 [−340.54, 293.78] 0.88	45.03 [−141.05, 231.12] 0.63	976.13 [691.58, 1260.68] < 0.01	17.30 [−5.55, 40.16] 0.14	1.00
Rh-istmus cingulate cortex	2486.14 [2374.44, 2597.84] < 0.01	−4.95 [−84.96, 75.07] 0.90	30.92 [−16.02, 77.87] 0.19	165.92 [94.14, 237.71] < 0.01	2.28 [−3.49, 8.04] 0.43	1.00
Rh-lateral occipital gyrus	11472.97 [11011.34, 11934.59] < 0.01	279.08 [−51.59, 609.76] 0.10	−83.68 [−277.69, 110.33] 0.39	585.83 [289.15, 882.50] < 0.01	0.63 [–23.20, 24.45] 0.96	1.00
Rh-lateral orbitofrontal	7862.07 [7547.79, 8176.34] < 0.01	−65.27 [−290.40, 159.85] 0.57	−47.48 [−179.56, 84.61] 0.48	627.49 [425.51, 829.47] < 0.01	6.01 [−10.21, 22.23] 0.46	1.00
Rh-Lingual	6665.20 [6338.75, 6991.66] < 0.01	−184.25 [−418.10, 49.60] 0.12	−86.56 [–223.77, 50.64] 0.21	305.99 [96.18, 515.80] < 0.01	−14.68 [−31.53, 2.17] 0.09	1.00
Rh-medial orbitofrontal	5842.69 [5677.05, 6008.33] < 0.01	−27.38 [−146.04, 91.27] 0.65	−27.24 [−96.86, 42.37] 0.44	453.36 [346.91, 559.82] < 0.01	5.72 [−2.83, 14.27] 0.19	1.00
Rh-middle temporal gyrus	12742.52 [12297.45, 13187.59] < 0.01	−208.94 [−527.75, 109.88] 0.20	−101.87 [−288.93, 85.18] 0.28	805.75 [519.71, 1091.79] < 0.01	−4.30 [−27.27, 18.67] 0.71	1.00
Rh-parahippocampal	2027.13 [1943.09, 2111.17] < 0.01	–33.02 [−93.22, 27.18] 0.28	9.97 [−25.35, 45.29] 0.58	85.41 [31.39, 139.42] < 0.01	−1.28 [−5.61, 3.06] 0.56	1.00
Rh-paracentral	3731.88 [3567.81, 3895.95] < 0.01	89.07 [−28.45, 206.60] 0.14	22.22 [−46.74, 91.17] 0.52	227.67 [122.22, 333.11] < 0.01	−1.21 [−9.68, 7.26] 0.78	1.00
Rh-pars opercularis	4240.00 [4048.04, 4431.95] < 0.01	−38.21 [−175.71, 99.29] 0.58	−86.41 [−167.09, −5.73] 0.04	378.87 [255.50, 502.24] < 0.01	−1.60 [−11.51, 8.30] 0.75	1.00
Rh-pars orbitalis	3141.37 [2973.16, 3309.59] < 0.01	−51.01 [−171.51, 69.48] 0.40	−41.97 [−112.66, 28.73] 0.24	243.59 [135.48, 351.70] < 0.01	1.86 [−6.82, 10.54] 0.67	1.00
Rh-pars triangularis	4519.52 [4275.88, 4763.16] < 0.01	17.23 [−157.30, 191.75] 0.84	−47.40 [−149.80, 55.00] 0.36	432.02 [275.44, 588.60] < 0.01	−10.64 [–23.21, 1.93] 0.10	1.00
Rh-pericalcarine	1976.61 [1824.96, 2128.26] < 0.01	−2.75 [−111.38, 105.88] 0.96	11.87 [−51.86, 75.61] 0.71	126.08 [28.62, 223.55] 0.01	−0.14 [−7.97, 7.69] 0.97	1.00
Rh-postcentral	9035.28 [8681.26, 9389.30] < 0.01	−104.30 [−357.89, 149.30] 0.42	108.34 [−40.45, 257.13] 0.15	799.36 [571.84, 1026.89] < 0.01	2.44 [−15.83, 20.71] 0.79	1.00
Rh-posterior cingulate cortex	3402.26 [3267.14, 3537.38] < 0.01	−96.12 [−192.91, 0.67] 0.05	−39.13 [−95.92, 17.66] 0.17	272.86 [186.02, 359.70] < 0.01	−0.49 [−7.47, 6.48] 0.89	0.95
Rh-precentral	13036.90 [12660.44, 13413.35] < 0.01	−152.86 [−422.53, 116.81] 0.26	73.24 [−84.98, 231.46] 0.36	591.68 [349.74, 833.63] < 0.01	9.83 [−9.60, 29.26] 0.32	1.00
Rh-precuneus	9806.17 [9471.65, 10140.69] < 0.01	203.01 [−36.62, 442.64] 0.10	79.14 [−61.45, 219.74] 0.27	855.76 [640.77, 1070.75] < 0.01	18.40 [1.14, 35.67] 0.04	1.00
Rh-rostral anterior cingulate cortex	2171.99 [2031.08, 2312.90] < 0.01	–23.79 [−124.73, 77.15] 0.64	−5.36 [−64.58, 53.86] 0.86	222.48 [131.92, 313.05] < 0.01	2.31 [−4.96, 9.58] 0.53	1.00
Rh-rostral middle frontal gyrus	17629.28 [17060.60, 18197.97] < 0.01	−328.36 [−735.73, 79.00] 0.11	−224.87 [−463.88, 14.14] 0.06	1630.65 [1265.17, 1996.14] < 0.01	−19.49 [−48.84, 9.86] 0.19	1.00
Rh-superior frontal gyrus	23630.92 [22918.92, 24342.92] < 0.01	265.43 [−244.59, 775.46] 0.30	48.39 [−250.85, 347.63] 0.75	1367.54 [909.95, 1825.13] < 0.01	31.82 [−4.93, 68.57] 0.09	1.00
Rh-superior parietal lobule	12759.24 [12221.69, 13296.79] < 0.01	37.48 [−347.58, 422.54] 0.85	88.39 [−137.53, 314.32] 0.44	995.85 [650.38, 1341.33] < 0.01	12.38 [−15.36, 40.13] 0.38	1.00
Rh-superior temporal	12049.58 [11614.71, 12484.46] < 0.01	−103.34 [−414.85, 208.18] 0.51	−82.16 [−264.94, 100.61] 0.37	888.55 [609.06, 1168.04] < 0.01	−6.83 [−29.28, 15.61] 0.55	1.00
Rh-supramarginal gyrus	10644.40 [10222.13, 11066.68] < 0.01	−115.44 [−417.92, 187.05] 0.45	20.66 [−156.82, 198.13] 0.82	878.24 [606.85, 1149.63] < 0.01	−1.29 [–23.09, 20.50] 0.91	1.00
Rh-frontal pole	1368.56 [1297.31, 1439.81] < 0.01	−17.34 [−68.38, 33.70] 0.50	−43.50 [−73.45, −13.56] < 0.01	39.98 [−5.81, 85.77] 0.09	0.44 [−3.24, 4.12] 0.81	1.00
Rh-temporal pole	2373.38 [2235.75, 2511.01] < 0.01	14.14 [−84.45, 112.72] 0.78	6.47 [−51.37, 64.32] 0.82	55.14 [–33.31, 143.60] 0.22	5.56 [−1.54, 12.66] 0.12	1.00
Rh-transverse temporal	885.77 [839.29, 932.25] < 0.01	−18.47 [−51.76, 14.83] 0.27	−5.20 [−24.74, 14.33] 0.60	70.79 [40.92, 100.67] < 0.01	−2.20 [−4.60, 0.20] 0.07	1.00
Rh-insula	7311.99 [7063.76, 7560.23] < 0.01	−125.88 [−303.70, 51.94] 0.16	25.34 [−78.99, 129.67] 0.63	460.63 [301.10, 620.17] < 0.01	3.52 [−9.29, 16.34] 0.59	1.00
Left-Thalamus-proper	8017.27 [7773.73, 8260.81] < 0.01	−54.97 [−229.42, 119.49] 0.53	11.22 [−91.14, 113.57] 0.83	632.77 [476.25, 789.29] < 0.01	10.28 [−2.29, 22.85] 0.11	1.00
Right-Thalamus-proper	7497.75 [7272.05, 7723.44] < 0.01	−64.40 [−226.08, 97.27] 0.43	−28.23 [−123.08, 66.63] 0.56	519.07 [374.02, 664.12] < 0.01	6.93 [−4.72, 18.58] 0.24	1.00
Left-Putamen	4076.83 [3882.01, 4271.66] < 0.01	137.23 [−2.34, 276.79] 0.05	−6.34 [−88.23, 75.54] 0.88	264.14 [138.92, 389.35] < 0.01	−2.61 [−12.67, 7.44] 0.61	0.96
Right-Putamen	4160.95 [3954.68, 4367.21] < 0.01	164.72 [16.96, 312.47] 0.03	13.56 [−73.13, 100.25] 0.76	254.08 [121.52, 386.65] < 0.01	−3.11 [−13.75, 7.54] 0.56	0.82
Left-Pallidum	1829.18 [1737.07, 1921.29] < 0.01	−6.18 [−72.16, 59.80] 0.85	20.11 [−18.60, 58.82] 0.30	141.76 [82.57, 200.96] < 0.01	−0.23 [−4.98, 4.53] 0.93	1.00
Right-Pallidum	1703.22 [1619.91, 1786.53] < 0.01	54.55 [−5.13, 114.23] 0.07	−8.81 [−43.82, 26.21] 0.62	128.33 [74.79, 181.87] < 0.01	−0.65 [−4.95, 3.65] 0.76	0.98
Left-Caudate	3417.94 [3289.71, 3546.17] < 0.01	−61.02 [−152.88, 30.84] 0.19	−49.41 [−103.31, 4.48] 0.07	276.55 [194.14, 358.97] < 0.01	−4.00 [−10.62, 2.62] 0.23	1.00
Right-Caudate	3392.33 [3267.31, 3517.35] < 0.01	−9.91 [−99.46, 79.65] 0.83	−50.75 [−103.30, 1.79] 0.06	282.01 [201.66, 362.36] < 0.01	−2.86 [−9.32, 3.59] 0.38	1.00
Left-Hippocampus	4046.78 [3945.42, 4148.14] < 0.01	−37.09 [−109.70, 35.51] 0.31	7.46 [−35.14, 50.06] 0.73	237.99 [172.84, 303.13] < 0.01	4.73 [−0.50, 9.96] 0.08	1.00
Right-Hippocampus	3961.64 [3857.38, 4065.90] < 0.01	0.28 [−74.40, 74.97] 0.99	15.75 [−28.07, 59.57] 0.48	218.93 [151.92, 285.93] < 0.01	−0.56 [−5.95, 4.82] 0.84	1.00
Left-Amygdala	1533.70 [1477.45, 1589.95] < 0.01	–22.81 [−63.10, 17.49] 0.26	16.54 [−7.10, 40.19] 0.17	84.50 [48.35, 120.66] < 0.01	0.31 [−2.59, 3.22] 0.83	1.00
Right-Amygdala	1640.47 [1587.89, 1693.05] < 0.01	−16.51 [−54.18, 21.15] 0.39	−2.97 [−25.06, 19.13] 0.79	106.72 [72.92, 140.51] < 0.01	1.68 [−1.03, 4.40] 0.22	1.00
Left-Accumbens-area	485.23 [438.22, 532.24] < 0.01	−15.62 [−49.30, 18.06] 0.36	9.95 [−9.81, 29.71] 0.32	38.07 [7.85, 68.28] 0.01	2.52 [0.10, 4.95] 0.04	1.00
Right-Accumbens-area	539.86 [509.95, 569.77] < 0.01	−15.12 [−36.55, 6.30] 0.16	5.45 [−7.12, 18.02] 0.39	35.06 [15.83, 54.28] < 0.01	0.35 [−1.19, 1.89] 0.65	1.00

Shown are parameter estimates (coefficients) with 95% confidence intervals.

PvalCor = Multiple comparison corrected p-values.

*Notes:* STiP-5.1=Semi-structured Interview for Personality Functioning DSM-5; ICV = Estimated Intracranial Volume; IQ = Intelligence Quotient.

### 3.4 Associations between the STiP-5.1 and ROIs

Associations between the STiP-5.1 total and each of the selected ROIs were all statistically non-significant (all corrected *p* values ≥0.91). Further, results for each element yielded no statistically significant associations (Identity: all corrected *p* values ≥0.92; Self-direction: all corrected *p* values ≥0.84; Empathy: all corrected *p* values ≥0.78; Intimacy: all corrected *p* values ≥0.83). See Tables S11–S15 in Supplementary Materials for full regression analysis results for STiP-5.1 total and each of the four elements for the ROIs.

### 3.5 Validity check using BPD criteria

Using BPD criteria as the predictor also yielded no statistically significant associations with total GMV (*p* = 0.33), nor for any of the individual brain regions (all corrected *p* values ≥0.55). The lowest uncorrected *p*-values found for the associations with BPD criteria were: Right caudal middle frontal gyrus (uncorrected *p* value = 0.01), right fusiform (uncorrected *p* value = 0.04), right posterior cingulate cortex (uncorrected *p* value = 0.03), right amygdala (uncorrected *p* value = 0.04). All ROIs yielded non-significant results (all corrected *p* values ≥ 0.28). See Tables S16–S18 in Supplementary Materials for regression analysis results with BPD criteria as the predictor variable.

## 4 Discussion

PF impairments represent core deficits in self- and interpersonal functioning along a continuum that are evident across all PD (especially BPD) according to the new dimensional classification systems of PD in the *DSM-5* AMPD and the *ICD-11.* The aim of the current study was to explore structural brain correlates with PF in youth – a sensitive period for the development of PDs, but also potentially representing a ‘purer’ developmental stage for exploration, with fewer confounding factors that might impact neuroanatomy. Overall, our findings indicate that there is no evidence for an association between dimensionally assessed PF severity (as a total STiP-5.1 score, or individual elements) and GMV in young females. Further, restricting the analyses to the ROIs predominantly studied in BPD (in the fronto-limbic network) did not yield any significant associations with PF either. Results were further confirmed when using number of BPD criteria as the predictor, which was also unrelated to brain structure.

Given that the brain volumetric correlates of PF have been rarely investigated, direct comparisons with previous dimensional studies are challenging. However, our results partly align with a recent study using two combined large community adult samples (total *N* > 2,000) showing no reliable associations between a composite measure of BPD traits (a proxy for dimensional PD) and structural brain variation in defined regions (Baranger et al., 2020). Conversely, our findings diverge from those of a recent study using diagnostic group comparisons (Yi et al., 2024). They found altered GMV in adolescents with (full-threshold categorical) BPD compared to HC in cortico-limbic regions and regions comprising the default mode network. However, it should be noted that in this study BPD participants were required to not have any Axis 1 DSM-IV disorder met – limiting generalizability of their findings (given that presentation of BPD without any comorbid psychiatric disorders is rare (Kaess et al., 2012), and they used different imaging and volume analysis (i.e., voxel-based morphometry (VBM)). Finally, our results are in contrast with earlier studies reporting structural alterations in specific ROI (e.g. OFC, ACC, DLPFC) in adolescents with a categorical BPD diagnosis (i.e., Brunner et al., 2010, Chanen et al., 2008, Goodman et al., 2011, Whittle et al., 2009). Overall, our findings align more closely with a large study using a (proxy) measure of dimensional BPD.

Results of the current study indicate no evidence of an association between PF and GMV, at least not in the early stages of (B)PD in adolescents. Using the dimensional personality pathology approach to exploring structural brain aberrations – which can help account for some of the previous challenges associated with research on categorical diagnoses – our findings challenge structural pathology assumptions in (B)PD. It is possible that dimensionally conceptualised PD may not be rooted in anatomical brain structures, and other biomarkers (e.g., neuroendocrinology, epigenetics, etc.; Marceau et al., 2018, Marceau et al., 2023, Ruocco and Carcone, 2016), may represent more promising avenues for clinical research in the context of PF impairments.

### 4.1 Strengths, limitations, and future directions

Key strengths of the this study include the use of the STiP-5.1 interview conducted by trained psychologists, the inclusion of youths across the full spectrum of PD symptom severity, a representative sample of youth with personality pathology and psychiatric comorbidities (Kaess et al., 2012), and our exploratory approach that extended beyond common fronto-limbic ROIs to examine individual brain regions across the whole brain using an established atlas. Naturally, some limitations should be acknowledged. Our entire sample comprised biologically female youths, and therefore, the current findings cannot be generalized to biological males. Indeed, although most sMRI studies on BPD comprise female only samples (and thus, empirical examination of sex differences is scarce), some work has found sex differences in relation to aggression in BPD, with reduced amygdala and hippocampal GMV in female BPD patients, but not male patients with BPD (Mancke et al., 2015). Another study found females with BPD (and abused females with BPD), but not males with BPD, had significant reductions in medial temporal lobe, including the amygdala. Furthermore, males with BPD, but not females with BPD, showed diminished grey matter concentrations in the anterior cingulate gyrus compared with findings in HC (Soloff et al., 2008). In adolescents, it has been found that males were more likely to have BPD symptoms if they were high on affiliation (representing a desire for closeness with others) but also had atypical rightward hippocampal asymmetry, and females were more likely to have higher BPD symptoms if they were low in effortful control (representing poor self-regulation) and had atypical rightward hippocampal asymmetry (Jovev et al., 2014). Future studies should consider actively recruiting male participants for sMRI studies to determine any sex-related differences in association with PF. Relatedly – although a common limitation in MRI studies (especially using clinical samples) – our sample size may be considered relatively modest, and future research will need to include larger samples sizes to confirm the current findings, which might be achieved by using multi-site studies. Our data are limited by their cross-sectional quality, limiting any inferences regarding change over time. To that end, future studies should conduct longitudinal sMRI research on PF throughout adolescence into adulthood to detect changes as a result of the developmental course, and differences in brain structure between youths and adults. Further, in our study, we focused on GMV, as this has been most commonly considered in sMRI literature on adolescent BPD. However, it is possible that surface, curvature, or white matter volume might yield differing results. Similarly, our ROI and atlas-based morphometric approach was used intentionally, as it aligns with a broader majority of adolescent BPD sMRI studies, enhancing comparability between findings. However, future studies could consider using VBM as an exploratory alternative, which may yield different results. It is also possible that the study of neurobiological substrates of PF impairment might be better suited to fMRI (including rsfMRI), where findings regarding self-referential and interpersonal processes (including emotion dysregulation in particular) in BPD have yielded more consistent findings (Bertsch and Herpertz, 2021a, Grandjean et al., 2024, Herpertz et al., 2018, Krause-Utz et al., 2014, O’Neill and Frodl, 2012, Ruocco et al., 2013, Schurz et al., 2024, Shafie et al., 2023, Visintin et al., 2016), and future studies should endeavour to further explore this in adolescent populations. Finally, as suggested earlier, heterogeneity of categorically defined PD may have posed a substantial challenge and impeded on neuroimaging studies in the past, and it is plausible that dimensional constructs overcome at least some of those challenges (i.e., within-category heterogeneity). However, even dimensionally conceptualised PF represents a complex construct which may still result in variable clinical presentation. Indeed, some of our previous work has shown that the latent structure of the STiP-5.1 comprises both dimensional and categorical components, and that adolescents may score in different patterns or classes (Thomson et al., 2024). Future work may consider examining structural alterations based on classes of adolescents with different PF impairment patterns.

### 4.2 Conclusions

Our study is the first to examine volumetric sMRI associations with PF in adolescents, using a validated clinical interview specifically designed to assess the dimensionally oriented Criterion A of the AMPD. While results indicate no association between PF and brain volume in young females, the current study contributes to the important goal of furthering the scientific understanding of dimensional constructs of PD such as in the AMPD, and the *ICD-11*. Current findings underscore the notoriously complex (and heterogeneous) clinical phenotype and multifactorial nature of (B)PD. Therefore, this may require the pursuit of clinical research efforts on other potential biomarkers (e.g., endocrinology, epigenetics, functional connectivity, etc.), using dimensional conceptualisations of PF to elucidate clinically meaningful phenotypes of personality pathology.

## Funding sources

Marialuisa Cavelti was supported by a grant from the 10.13039/501100001711Swiss National Science Foundation (PZ00P1_193279). This research did not receive any specific grant from funding agencies in the public, commercial, or not-for-profit sectors.

## CRediT authorship contribution statement

**Madelyn Thomson:** Writing – original draft, Formal analysis, Data curation, Conceptualization. **Marialuisa Cavelti:** Writing – review & editing, Supervision, Methodology, Conceptualization. **Ines Mürner-Lavanchy:** Writing – review & editing, Methodology, Data curation. **Silvano Sele:** Writing – review & editing, Methodology, Formal analysis. **Niklas Bürgi:** Writing – review & editing, Methodology. **Nora Seiffert:** Writing – review & editing, Project administration, Investigation. **Franz Moggi:** Resources, Methodology, Investigation. **Roland Wiest:** Resources, Methodology, Investigation. **Julian Koenig:** Writing – review & editing, Supervision. **Michael Kaess:** Writing – review & editing, Supervision, Resources, Methodology, Funding acquisition, Conceptualization.

## Declaration of competing interest

The authors declare that they have no known competing financial interests or personal relationships that could have appeared to influence the work reported in this paper.

## Data Availability

The datasets generated and/or analysed in the current study are not publicly available as participants did not provide consent for this purpose. Data can be made available from the corresponding author upon reasonable request.
