# Supplementary material for: Grey matter volume associations with personality functioning in a clinical cohort of female youths
Source: Neuroimage Clin. 2026 Mar 28;50:103988. doi: 10.1016/j.nicl.2026.103988 (PMC13068870; doi:10.1016/j.nicl.2026.103988)
Supplement: Supplementary Data 1 [file mmc1.docx]

**Supplementary Material for:**

***“Grey matter volume associations with personality functioning in a clinical cohort of female youths”***

Madelyn Thomson, Marialuisa Cavelti, Ines Mürner-Lavanchy, Silvano Sele, Niklas Bürgi, Nora Seiffert, Franz Moggi, Roland Wiest, Julian Koenig, Michael Kaess

Content: 2 Supplementary Texts, 18 Supplementary Tables

- Text S1: Online measures and clinical assessments used in the study
- Table S1: List and abbreviations of (82) regions included for analysis (Desikan-Killiany Tourville Atlas) by left and right hemispheres
- Text S2: Regression model iterations and testing for STiP-5.1 × Age interaction term
- Table S2: Regression model selection including tests for STiP-5.1 × Age interaction (*N*=93)
- Table S3: Regression analysis results of STiP-5.1 Identity on total GMV (*N*=93)
- Table S4: Regression analysis results of STiP-5.1 Self-direction on total GMV (*N*=93)
- Table S5: Regression analysis results of STiP-5.1 Empathy on total GMV (*N*=93)
- Table S6: Regression analysis results of STiP-5.1Intimacy on total GMV (*N*=93)
- Table S7: Regression analysis results of STiP-5.1 Identity on individual brain region GMV (*N*=93)
- Table S8: Regression analysis results of STiP-5.1 Self-direction on individual brain region GMV (*N*=93)
- Table S9: Regression analysis results of STiP-5.1 Empathy on individual brain region GMV (*N*=93)
- Table S10: Regression analysis results of STiP-5.1 Intimacy on individual brain region GMV (*N*=93)
- Table S11: Regression analysis results of STiP-5.1 total scores on selected ROIs GMV (*N*=93)
- Table S12: Regression analysis results of STiP-5.1 Identity on selected ROIs GMV (*N*=93)
- Table S13: Regression analysis results for Self-direction predicting selected ROIs GMV (*N*=93)
- Table S14: Regression analysis results of STiP-5.1 Empathy on selected ROIs GMV (*N*=93)
- Table S15: Regression analysis results for Intimacy predicting selected ROIs GMV (*N*=93)
- Table S16: Regression analysis results of BPD criteria on total GMV (*N*=93)
- Table S17: Regression analysis results of BPD criteria on individual brain region GMV (*N*=93)
- Table S18: Regression analysis results of BPD criteria on selected ROIs GMV (*N*=93)

**Text S1.** *Online measures and clinical assessments used in the study*

German versions of all measures were used in the current study.

**Online questionnaires**

*Depression severity*. The Beck Depression Inventory II (BDI-II; (1) was used to assess the severity of depressive symptoms. It contains of 21 multiple-choice items that are scored on a scale ranging from 0 to 3. The BDI-II is validated for adolescents (2), and the German version demonstrates good psychometric properties (3).

*Childhood trauma.* Childhood trauma was assessed using the Childhood Trauma Questionnaire (CTQ; (4). The CTQ is a 28-item self-report retrospective inventory to measure childhood or adolescent emotional, physical, and sexual abuse, and emotional and physical neglect. A 5-point scale is used for responses ranging from “never true” to “very often true.” The German version has adequate psychometric properties (5).

**Clinical assessments**

*Sociodemographic data.* Information regarding age, schooling, living situation, and medication use was collected using a standardised set of interview questions.

*Handedness.* The Edinburgh Handedness Inventory (6) was used to assess handedness. The EHI is one of the most widely used instruments for this purpose. It quantifies handedness by querying handedness behaviour in relation to several everyday unimanual activities (e.g., writing, using a toothbrush, etc.) and respondents report whether they tend to use one hand more than the other, and if so, the strength of the tendency.

*Personality Functioning.* PF was measured using the STiP-5.1 (7) which assesses the severity of impairments in PF reflected in the Level of Personality Functioning Scale (LPFS) of the AMPD, comprising self- and interpersonal-functioning. Self- and interpersonal functioning further comprise four elements (identity, self-direction, empathy, and intimacy) with 3 facets each, for a total of 12 facets. For each of these, the level of functioning can be determined based on five levels ranging from *little to no impairment* (0), *mild* (1), *moderate* (2), *severe* (3) *or extreme* (4) *impairment in functioning*, (higher scores indicating higher levels of impairment). Scores are summed, and a mean score is established from this for an overall score. The STiP-5.1 has been found to have good psychometric properties including high internal consistency (Cronbach’s α=0.97), high inter-rater reliability (interclass correlations ranging from .81 to .92), good constructive validity, and is shown to distinguish between ‘normal’ and ‘clinical’ respondents, as well as between those with and without PD (8). Importantly, it has also demonstrated good psychometric properties in adolescents (9). For the current study, Criterion A (Impairment in PF) was considered fulfilled according to *DSM-5* AMPD guidance. While the diagnostic threshold required for *general* PD is an overall mean score of two (*moderate impairment*), we also applied AMPD guidance for *trait-specified* and *specific* PD for descriptive statistics purposes, whereby the individual must score two or higher (moderate or greater) on two or more elements for a PD diagnosis. This is to accommodate broader clinical applicability where clinicians may also use Criterion A in the context of *trait specified* or *specific* PD, wherein the option for a BPD diagnosis in the hybrid format is also possible.

*Borderline Personality Disorder criteria*. BPD was measured using the Borderline-Module of the Structured Clinical Interview for DSM-IV Axis II Disorders (SCID-II) (10). The SCID-II is a structured clinical interview for the detection of PD according to *DSM-IV*. The BPD module examines the presence of the nine criteria defined by *DSM-IV*. Each item is scored on a three-point scale (1=absent, 2=subthreshold, 3=present). The diagnosis requires that five or more criteria are met. Aligning with previous literature, a BPD feature is scored present if it has been present for two years for individuals older than 18 years, and 1 year for those under the age of 18 years (11). In the current study, the BPD module of the SCID-II was used to ensure the presence of BPD features for the patient groups, and the absence of BPD features for the HC.

*Psychiatric diagnoses.* The Mini-International Neuropsychiatric Interview for Children and Adolescents, (MINI-KID; (12) was used to assess co-occurring psychiatric disorders in the BPD groups. It is a short, structured clinical interview for the assessment of mental disorders in children and adolescents according to *DSM-IV* and *ICD-10*.

*Absence of psychiatric disorders for HC.* To establish eligibility for the study as a HC, the Structured Clinical Interview for *DSM-IV-TR*, non-patient edition (SCID-N/P; (13) was used to ensure the absence of any psychiatric disorders.

*Intelligence Quotient.* The German versions of the Wechsler Intelligence Scale for Children – Fifth Edition (WISC-V; (14) and the Wechsler Adult Intelligence Scale – Fourth Edition (WAIS-IV; (15) were used to assess intelligence quotient (IQ) in participants 14-16 years, and above 16 years of age, respectively.

*MRI Safety Screening.* An MRI safety screening questionnaire was applied before participants entered the scanner, in order to confirm that they do not meet any contraindication for MRI (e.g., metal implants, body piercing, pregnancy).

**Table S1.** *List and abbreviations of (82) regions included for analysis (Desikan-Killiany Tourville Atlas) by left and right hemispheres*

| **Left hemisphere** | | **Right hemisphere** | |
| --- | --- | --- | --- |
| *Abbreviation* | *DKT region name* | *Abbreviation* | *DKT region name* |
| **Cortical regions** | | **Cortical regions** | |
| L.BSTS | Lh-banks superior temporal sulcus | R.BSTS | Rh-banks superior temporal sulcus |
| L.cACC | Lh-caudal anterior cingulate cortex | R.cACC | Rh-caudal anterior cingulate cortex |
| L.cMFG | Lh-caudal middle frontal gyrus | R.cMFG | Rh-caudal middle frontal gyrus |
| L.CUN | Lh-cuneus | R.CUN | Rh-cuneus |
| L.ENT | Lh-entorhinal | R.ENT | Rh-entorhinal |
| L.FUS | Lh-fusiform | R.FUS | Rh-fusiform |
| L.IPL | Lh-inferior parietal lobule | R.IPL | Rh-inferior parietal lobule |
| L.ITG | Lh- inferior temporal gyrus | R.ITG | Rh-inferior temporal gyrus |
| L.iCC | Lh-istmus cingulate cortex | R.iCC | Rh-istmus cingulate cortex |
| L.LOG | Lh-lateral occipital gyrus | R.LOG | Rh-lateral occipital gyrus |
| L.LOF | Lh-lateral orbitofrontal | R.LOF | Rh-lateral orbitofrontal |
| L.LING | Lh-Lingual | R.LING | Rh-Lingual |
| L.MOF | Lh-medial orbitofrontal | R.MOF | Rh-medial orbitofrontal |
| L.MTG | Lh-middle temporal gyrus | R.MTG | Rh-middle temporal gyrus |
| L.PARH | Lh-parahippocampal | R.PARH | Rh-parahippocampal |
| L.paraC | Lh-paracentral | R.paraC | Rh-paracentral |
| L.pOPER | Lh-pars opercularis | R.pOPER | Rh-pars opercularis |
| L.pORB | Lh-pars orbitalis | R.pORB | Rh-pars orbitalis |
| L.pTRI | Lh-pars triangularis | R.pTRI | Rh-pars triangularis |
| L.periCAL | Lh-pericalcarine | R.periCAL | Rh-pericalcarine |
| L.postC | Lh-postcentral | R.postC | Rh-postcentral |
| L.PCC | Lh-posterior cingulate cortex | R.PCC | Rh-posterior cingulate cortex |
| L.preC | Lh-precentral | R.preC | Rh-precentral |
| L.PCUN | Lh-precuneus | R.PCUN | Rh-precuneus |
| L.rACC | Lh-rostral anterior cingulate cortex | R.rACC | Rh-rostral anterior cingulate cortex |
| L.rMFG | Lh-rostral middle frontal gyrus | R.rMFG | Rh-rostral middle frontal gyrus |
| L.SFG | Lh-superior frontal gyrus | R.SFG | Rh-superior frontal gyrus |
| L.SPL | Lh-superior parietal lobule | R.SPL | Rh-superior parietal lobule |
| L.STG | Lh-superior temporal gyrus | R.STG | Rh-superior temporal |
| L.SMAR | Lh-supramarginal gyrus | R.SMAR | Rh-supramarginal gyrus |
| L.FP | Lh-frontal pole | R.FP | Rh-frontal pole |
| L.TP | Lh-temporal pole | R.TP | Rh-temporal pole |
| L.TT | Lh-transverse temporal | R.TT | Rh-transverse temporal |
| L.INS | Lh-insula | R.INS | Rh-insula |
| **Subcortical regions** | | **Subcortical regions** | |
| L.THAL | Left-Thalamus-proper | R.THAL | Right-Thalamus-proper |
| L.PUT | Left-Putamen | R.PUT | Right-Putamen |
| L.PALL | Left-Pallidum | R.PALL | Right-Pallidum |
| L.CAUD | Left-Caudate | R.CAUD | Right-Caudate |
| L.HIPP | Left-Hippocampus | R.HIPP | Right-Hippocampus |
| L.AMYG | Left-Amygdala | R.AMYG | Right-Amygdala |
| L.AC | Left-Accumbens-area | R.AC | Right-Accumbens-area |

**Text S2.** *Regression model iterations and testing for STiP-5.1 × Age interaction term*

Age, sex, estimated intracranial volume (ICV), and intelligence quotient (IQ) are among the most commonly included covariates in structural neuroimaging studies (16). Therefore, we included these in the overall model used for the subsequent main analyses (with the exception of sex, as our entire sample was female). For transparency purposes, however, we modelled various iterations with i) no covariates included – a ‘basic’ model (i.e., only STiP-5.1 as a predictor); ii) individually including each covariate, and; iii) covariates added sequentially. Using total GMV as the outcome variable in these model variations, the reader can view the effect of each individual covariate, as well as the additive effects of sequential inclusion of covariates (on total GMV). See Table S2 for results of all iterated models. Further, given the (normative) age-related dynamic structural changes in the brain throughout adolescence (17), which may also coincide with vulnerability to psychopathology (18,19), and that our sample represents this developmentally sensitive period of protracted brain development (20,21), we sought to determine if a STiP-5.1 × Age interaction term was indicated for the main analyses. Therefore, we tested for this by adding the interaction term into separate versions of the aforementioned iterations of the models (models E, F, G, H in Table S2). There was no evidence for a STiP-5.1 × Age interaction in any of the models (all *p*≥ 0.38). Therefore, the final chosen (main) model was a simpler version as follows: STiP-5.1 total score (or element) as the predictor variable, and age, ICV and IQ included as covariates. ICV, IQ and age were mean centred for all analyses.

**Table S2.** *Regression model selection including tests for STiP-5.1 × Age interaction on total GMV (N=93)*

|  | A | B | C | D | E | F | G | H | I | J | K | L |
| --- | --- | --- | --- | --- | --- | --- | --- | --- | --- | --- | --- | --- |
| (Intercept) | 673353.33 [656469.74, 690236.92] <0.01 | 658847.95 [649238.06, 668457.84] <0.01 | 574117.80 [481421.56, 666814.05] <0.01 | 637112.96 [583622.73, 690603.19] <0.01 | 673577.79 [656259.78, 690895.81] <0.01 | 658231.78 [648398.22, 668065.34] <0.01 | 574085.28 [479660.46, 668510.10] <0.01 | 638397.43 [584097.48, 692697.38] <0.01 | 673518.33 [656491.60, 690545.06] <0.01 | 658934.24 [649240.53, 668627.95] <0.01 | 574263.60 [480399.41, 668127.78] <0.01 | 637544.57 [583385.94, 691703.21] <0.01 |
| STiP-5.1 total | -15308.34 [-27094.91, -3521.78] 0.01 | -2312.65 [-9116.41, 4491.11] 0.50 | -11964.69 [-23923.71, -5.68] 0.05 | -1735.31 [-8694.28, 5223.65] 0.62 | -15555.06 [-28074.71, -3035.41] 0.02 | -1450.88 [-8663.41, 5761.64] 0.69 | -12251.24 [-24913.01, 410.54] 0.06 | -947.60 [-8305.82, 6410.62] 0.80 | -15472.27 [-27396.37, -3548.17] 0.01 | -2398.04 [-9282.11, 4486.03] 0.49 | -11986.56 [-24124.91, 151.79] 0.05 | -1798.75 [-8857.65, 5260.15] 0.61 |
| ICV |  | 44163.75 [38019.80, 50307.71] <0.01 |  | 43616.41 [37319.36, 49913.46] <0.01 |  | 44342.04 [38140.85, 50543.23] <0.01 |  | 43835.33 [37469.18, 50201.48] <0.01 |  | 44153.16 [37974.79, 50331.52] <0.01 |  | 43619.29 [37286.11, 49952.48] <0.01 |
| IQ |  |  | 940.91 [76.13, 1805.68] 0.03 | 207.79 [-295.23, 710.80] 0.41 |  |  | 943.27 [62.65, 1823.89] 0.04 | 189.71 [-321.02, 700.43] 0.46 |  |  | 939.69 [65.22, 1814.17] 0.04 | 204.18 [-304.41, 712.76] 0.43 |
| Age |  |  |  |  | -660.65 [-13793.57, 12472.26] 0.92 | -3140.44 [-10426.10, 4145.23] 0.39 | 743.22 [-12205.19, 13691.62] 0.91 | -2829.76 [-10183.22, 4523.71] 0.45 | -910.14 [-8294.85, 6474.57] 0.81 | -456.79 [-4567.34, 3653.76] 0.83 | -97.44 [-7380.82, 7185.93] 0.98 | -285.69 [-4427.29, 3855.91] 0.89 |
| STiP-5.1 total × Age |  |  |  |  | -222.33 [-9874.36, 9429.70] 0.96 | 2393.25 [-2967.77, 7754.27] 0.38 | -746.39 [-10226.57, 8733.78] 0.88 | 2257.96 [-3130.15, 7646.07] 0.41 |  |  |  |  |
| Num.Obs. | 93 | 93 | 93 | 93 | 93 | 93 | 93 | 93 | 93 | 93 | 93 | 93 |
| R2 | 0.068 | 0.715 | 0.114 | 0.717 | 0.069 | 0.717 | 0.114 | 0.719 | 0.069 | 0.715 | 0.114 | 0.717 |
| Shown are parameter estimates with 95% confidence intervals  *Notess:* STiP-5.1=Semi-structured Interview for Personality Functioning DSM-5; GMV=grey matter volume; ICV=Estimated Intracranial Volume; IQ=Intelligence Quotient; Model A: STiP-5.1 as predictor, no covariates; Model B: STiP5.1 as predictor, ICV as covariate; Model C: STiP-5.1 as predictor, IQ as covariate; Model D: STiP-5.1 as predictor, ICV and IQ as covariates; Model E: STiP-5.1 as predictor, Age as covariate with STiP-5.1 × Age interaction; Model F: STiP-5.1 as predictor, ICV and Age as covariates with STiP-5.1 × Age interaction; Model G: STiP-5.1 as predictor, IQ and Age as covariate with STiP-5.1 × Age interaction; Model H: STiP-5.1 as predictor, ICV, IQ and Age as covariates with STiP-5.1 × Age interaction; Model I: STiP-5.1 as predictor, Age as covariate; Model J: STiP-5.1 as predictor, ICV and Age as covariates; Model K: STiP-5.1 as predictor, IQ and Age as covariates; Model L: STiP-5.1 as predictor, ICV, IQ and Age as covariates. | | | | | | | | | | | | |

**Table S3*.*** *Regression analysis results of STiP-5.1 Identity on total GMV (N=93)*

| Predictors | Estimates, [95% CI] p value |
| --- | --- |
| (Intercept) | 658635.17 [648864.22, 668406.12] <0.01 |
| STiP-5.1 Identity | -1418.54 [-6067.31, 3230.23] 0.55 |
| Age | -322.35 [-4465.57, 3820.86] 0.88 |
| ICV | 43520.35 [37168.05, 49872.65] <0.01 |
| IQ | 213.21 [-287.20, 713.62] 0.40 |
| Num.Obs. | 93 |
| R2 | 0.717 |
| Shown are parameter estimates with 95% confidence intervals *Notess:* STiP-5.1=Semi-structured Interview for Personality Functioning DSM-5; GMV=grey matter volume; ICV=Estimated Intracranial Volume; IQ=Intelligence Quotient | |

**Table S4.** *Regression analysis results of STiP-5.1 Self-direction on total GMV (N=93)*

| Predictors | Estimates, [95% CI] p value |
| --- | --- |
| (Intercept) | 659598.54 [650220.98, 668976.10] <0.01 |
| STiP-5.1 Self-direction | -3014.07 [-9585.18, 3557.05] 0.36 |
| Age | -326.14 [-4437.50, 3785.21] 0.88 |
| ICV | 43253.99 [36893.79, 49614.19] <0.01 |
| IQ | 188.26 [-316.14, 692.66] 0.46 |
| Num.Obs. | 93 |
| R2 | 0.719 |
| Shown are parameter estimates with 95% confidence intervals *Notes:* STiP-5.1=Semi-structured Interview for Personality Functioning DSM-5; GMV=grey matter volume; ICV=Estimated Intracranial Volume; IQ=Intelligence Quotient | |

**Table S5.** *Regression analysis results of STiP-5.1 Empathy on total GMV (N=93)*

| Predictors | Estimates, [95% CI] p value |
| --- | --- |
| (Intercept) | 658488.69 [649432.94, 667544.45] <0.01 |
| STiP-5.1 Empathy | -2918.51 [-11898.16, 6061.15] 0.52 |
| Age | -305.04 [-4435.89, 3825.82] 0.88 |
| ICV | 43694.85 [37455.36, 49934.33] <0.01 |
| IQ | 168.32 [-365.09, 701.74] 0.53 |
| Num.Obs. | 93 |
| R2 | 0.717 |
| Shown are parameter estimates with 95% confidence intervals *Notes:* STiP-5.1=Semi-structured Interview for Personality Functioning DSM-5; GMV=grey matter volume; ICV=Estimated Intracranial Volume; IQ=Intelligence Quotient | |

**Table S6.** *Regression analysis results of STiP-5.1Intimacy on total GMV (N=93)*

| Predictors | Estimates, [95% CI] p value |
| --- | --- |
| (Intercept) | 655372.16 [647201.93, 663542.39] <0.01 |
| STiP-5.1 Intimacy | 983.81 [-5098.36, 7065.98] 0.75 |
| Age | -79.14 [-4211.13, 4052.86] 0.97 |
| ICV | 44076.01 [37834.89, 50317.14] <0.01 |
| IQ | 243.33 [-259.46, 746.12] 0.34 |
| Num.Obs. | 93 |
| R2 | 0.716 |
| Shown are parameter estimates with 95% confidence intervals *Notes:* STiP-5.1=Semi-structured Interview for Personality Functioning DSM-5; GMV=grey matter volume; ICV=Estimated Intracranial Volume; IQ=Intelligence Quotient | |

**Table S7.** *Regression analysis results of STiP-5.1 Identity on individual brain region GMV (N=93)*

|  | **(Intercept)** | **STiP-5.1 Identity** | **Age** | **ICV** | **IQ** | **PvalCor** | |
| --- | --- | --- | --- | --- | --- | --- | --- |
| L.BSTS | 2505.98 [2360.99, 2650.97], <0.01 | -14.11 [-83.10, 54.87], 0.69 | -16.82 [-78.30, 44.66], 0.59 | 259.49 [165.23, 353.76], <0.01 | -4.15 [-11.57, 3.28], 0.27 | | 1.00 |
| L.cACC | 1838.02 [1675.47, 2000.58], <0.01 | 15.83 [-61.51, 93.18], 0.69 | 15.31 [-53.62, 84.24], 0.66 | 179.64 [73.96, 285.33], <0.01 | 0.13 [-8.19, 8.46], 0.98 | | 1.00 |
| L.cMFG | 6989.32 [6636.75, 7341.88], <0.01 | -114.95 [-282.69, 52.79], 0.18 | -178.18 [-327.68, -28.68], 0.02 | 233.72 [4.51, 462.92], 0.05 | -1.30 [-19.36, 16.75], 0.89 | | 1.00 |
| L.CUN | 2936.14 [2750.69, 3121.60], <0.01 | -57.67 [-145.91, 30.57], 0.20 | 2.35 [-76.29, 80.99], 0.95 | 216.99 [96.42, 337.56], <0.01 | 0.45 [-9.05, 9.95], 0.93 | | 1.00 |
| L.ENT | 2038.92 [1884.52, 2193.33], <0.01 | -60.44 [-133.90, 13.02], 0.11 | 0.39 [-65.09, 65.86], 0.99 | 58.37 [-42.02, 158.75], 0.25 | 0.35 [-7.55, 8.26], 0.93 | | 1.00 |
| L.FUS | 9540.77 [9216.50, 9865.03], <0.01 | -91.29 [-245.57, 62.98], 0.24 | 89.65 [-47.85, 227.14], 0.20 | 733.89 [523.08, 944.70], <0.01 | 12.07 [-4.54, 28.68], 0.15 | | 1.00 |
| L.IPL | 12297.86 [11785.77, 12809.95], <0.01 | 50.06 [-193.58, 293.70], 0.68 | 15.85 [-201.29, 233.00], 0.88 | 1023.25 [690.33, 1356.17], <0.01 | 3.38 [-22.85, 29.60], 0.80 | | 1.00 |
| L.ITG | 11501.06 [10957.29, 12044.84], <0.01 | -159.76 [-418.48, 98.95], 0.22 | -55.60 [-286.18, 174.98], 0.63 | 792.37 [438.85, 1145.89], <0.01 | 2.65 [-25.19, 30.50], 0.85 | | 1.00 |
| L.iCC | 2598.00 [2508.22, 2687.77], <0.01 | -5.72 [-48.43, 36.99], 0.79 | -20.79 [-58.86, 17.28], 0.28 | 177.97 [119.61, 236.33], <0.01 | 3.03 [-1.57, 7.63], 0.19 | | 1.00 |
| L.LOG | 11614.63 [11183.36, 12045.91], <0.01 | -23.86 [-229.04, 181.33], 0.82 | 21.17 [-161.70, 204.05], 0.82 | 701.85 [421.47, 982.24], <0.01 | -5.87 [-27.96, 16.22], 0.60 | | 1.00 |
| L.LOF | 7961.89 [7663.18, 8260.59], <0.01 | -30.36 [-172.48, 111.76], 0.67 | 23.45 [-103.21, 150.12], 0.71 | 491.33 [297.13, 685.52], <0.01 | 16.49 [1.19, 31.79], 0.03 | | 1.00 |
| L.LING | 6092.95 [5799.53, 6386.37], <0.01 | -102.91 [-242.51, 36.69], 0.15 | -80.50 [-204.92, 43.91], 0.20 | 327.01 [136.25, 517.76], <0.01 | 2.19 [-12.84, 17.22], 0.77 | | 1.00 |
| L.MOF | 5661.71 [5439.28, 5884.13], <0.01 | 17.66 [-88.16, 123.48], 0.74 | -42.08 [-136.39, 52.23], 0.38 | 454.66 [310.06, 599.26], <0.01 | 5.53 [-5.86, 16.92], 0.34 | | 1.00 |
| L.MTG | 11334.14 [10853.64, 11814.64], <0.01 | 20.59 [-208.02, 249.20], 0.86 | -106.61 [-310.36, 97.14], 0.30 | 914.31 [601.92, 1226.69], <0.01 | -13.98 [-38.59, 10.63], 0.26 | | 1.00 |
| L.PARH | 2127.15 [2035.05, 2219.26], <0.01 | -10.63 [-54.45, 33.19], 0.63 | -11.78 [-50.84, 27.28], 0.55 | 52.88 [-7.00, 112.76], 0.08 | 6.11 [1.39, 10.83], 0.01 | | 1.00 |
| L.paraC | 3460.63 [3320.99, 3600.26], <0.01 | 11.28 [-55.16, 77.72], 0.74 | 7.41 [-51.80, 66.62], 0.80 | 215.38 [124.60, 306.16], <0.01 | 3.67 [-3.48, 10.82], 0.31 | | 1.00 |
| L.pOPER | 4951.93 [4730.73, 5173.12], <0.01 | 63.75 [-41.49, 168.99], 0.23 | -110.47 [-204.26, -16.68], 0.02 | 399.40 [255.60, 543.20], <0.01 | 2.35 [-8.98, 13.68], 0.68 | | 1.00 |
| L.pORB | 2682.53 [2543.19, 2821.88], <0.01 | -12.72 [-79.02, 53.58], 0.70 | 12.44 [-46.65, 71.52], 0.68 | 223.13 [132.54, 313.73], <0.01 | 4.46 [-2.67, 11.60], 0.22 | | 1.00 |
| L.pTRI | 4081.78 [3876.06, 4287.51], <0.01 | -10.52 [-108.40, 87.36], 0.83 | -3.78 [-91.02, 83.45], 0.93 | 390.51 [256.76, 524.26], <0.01 | 5.41 [-5.13, 15.95], 0.31 | | 1.00 |
| L.periCAL | 1866.46 [1729.84, 2003.08], <0.01 | -38.77 [-103.77, 26.23], 0.24 | 1.70 [-56.24, 59.63], 0.95 | 116.30 [27.48, 205.11], 0.01 | 0.20 [-6.80, 7.20], 0.95 | | 1.00 |
| L.postC | 9245.46 [8875.22, 9615.70], <0.01 | 15.04 [-161.11, 191.19], 0.87 | 106.71 [-50.28, 263.70], 0.18 | 753.94 [513.24, 994.64], <0.01 | 2.12 [-16.84, 21.08], 0.82 | | 1.00 |
| L.PCC | 3209.76 [3064.82, 3354.71], <0.01 | -15.57 [-84.53, 53.39], 0.65 | 29.43 [-32.03, 90.89], 0.34 | 214.58 [120.35, 308.81], <0.01 | 6.11 [-1.32, 13.53], 0.11 | | 1.00 |
| L.preC | 13305.98 [12909.87, 13702.10], <0.01 | -59.15 [-247.61, 129.31], 0.53 | -75.92 [-243.89, 92.05], 0.37 | 770.93 [513.41, 1028.45], <0.01 | 1.91 [-18.37, 22.20], 0.85 | | 1.00 |
| L.PCUN | 9611.13 [9294.15, 9928.11], <0.01 | 23.47 [-127.34, 174.28], 0.76 | 101.03 [-33.38, 235.44], 0.14 | 782.86 [576.78, 988.93], <0.01 | 9.58 [-6.65, 25.82], 0.24 | | 1.00 |
| L.rACC | 2840.39 [2681.75, 2999.03], <0.01 | -74.75 [-150.23, 0.73], 0.05 | 56.60 [-10.67, 123.86], 0.10 | 208.65 [105.51, 311.78], <0.01 | 5.00 [-3.13, 13.12], 0.22 | | 0.95 |
| L.rMFG | 17445.45 [16908.97, 17981.92], <0.01 | -69.35 [-324.60, 185.89], 0.59 | -106.75 [-334.24, 120.73], 0.35 | 1623.77 [1275.00, 1972.55], <0.01 | -1.62 [-29.10, 25.85], 0.91 | | 1.00 |
| L.SFG | 24849.10 [24144.25, 25553.95], <0.01 | 66.33 [-269.02, 401.68], 0.70 | 6.60 [-292.28, 305.49], 0.97 | 1871.11 [1412.87, 2329.35], <0.01 | 24.47 [-11.63, 60.56], 0.18 | | 1.00 |
| L.SPL | 13044.75 [12510.22, 13579.29], <0.01 | 14.07 [-240.25, 268.38], 0.91 | 132.34 [-94.33, 359.00], 0.25 | 935.26 [587.74, 1282.77], <0.01 | 0.94 [-26.44, 28.31], 0.95 | | 1.00 |
| L.STG | 12590.01 [12166.24, 13013.79], <0.01 | -57.29 [-258.92, 144.33], 0.57 | 23.96 [-155.74, 203.66], 0.79 | 877.84 [602.34, 1153.35], <0.01 | -3.57 [-25.27, 18.13], 0.74 | | 1.00 |
| L.SMAR | 11679.49 [11099.14, 12259.83], <0.01 | 91.33 [-184.79, 367.44], 0.51 | -4.26 [-250.35, 241.82], 0.97 | 1217.85 [840.56, 1595.15], <0.01 | 9.90 [-19.82, 39.62], 0.51 | | 1.00 |
| L.FP | 1117.13 [1062.42, 1171.84], <0.01 | 6.70 [-19.33, 32.74], 0.61 | -19.86 [-43.06, 3.35], 0.09 | 23.63 [-11.94, 59.20], 0.19 | 0.86 [-1.95, 3.66], 0.55 | | 1.00 |
| L.TP | 2449.62 [2271.23, 2628.01], <0.01 | -32.84 [-117.71, 52.04], 0.44 | 35.37 [-40.27, 111.02], 0.36 | 34.23 [-81.75, 150.21], 0.56 | 2.51 [-6.62, 11.65], 0.59 | | 1.00 |
| L.TT | 1082.42 [1021.24, 1143.61], <0.01 | -8.37 [-37.48, 20.74], 0.57 | 9.03 [-16.91, 34.98], 0.49 | 60.82 [21.04, 100.60], <0.01 | -0.69 [-3.83, 2.44], 0.66 | | 1.00 |
| L.INS | 7248.51 [7037.04, 7459.97], <0.01 | -35.77 [-136.38, 64.84], 0.48 | 30.00 [-59.66, 119.67], 0.51 | 385.73 [248.25, 523.20], <0.01 | 6.13 [-4.70, 16.96], 0.26 | | 1.00 |
| R.BSTS | 2376.85 [2261.44, 2492.25], <0.01 | -14.37 [-69.28, 40.54], 0.60 | -15.78 [-64.72, 33.15], 0.52 | 173.18 [98.15, 248.20], <0.01 | -0.58 [-6.49, 5.33], 0.85 | | 1.00 |
| R.cACC | 2037.78 [1883.84, 2191.71], <0.01 | 17.06 [-56.18, 90.30], 0.64 | -12.74 [-78.01, 52.54], 0.70 | 238.78 [138.71, 338.86], <0.01 | 1.29 [-6.60, 9.17], 0.75 | | 1.00 |
| R.cMFG | 6913.88 [6588.28, 7239.48], <0.01 | -179.02 [-333.93, -24.11], 0.02 | -38.45 [-176.51, 99.62], 0.58 | 478.39 [266.71, 690.07], <0.01 | 12.80 [-3.88, 29.47], 0.13 | | 0.76 |
| R.CUN | 3212.28 [3016.78, 3407.77], <0.01 | -47.91 [-140.93, 45.10], 0.31 | -18.76 [-101.65, 64.14], 0.65 | 164.53 [37.43, 291.62], 0.01 | -3.77 [-13.78, 6.25], 0.46 | | 1.00 |
| R.ENT | 1890.09 [1758.56, 2021.62], <0.01 | -37.03 [-99.61, 25.54], 0.24 | -28.66 [-84.44, 27.11], 0.31 | 82.16 [-3.35, 167.67], 0.06 | 2.05 [-4.69, 8.78], 0.55 | | 1.00 |
| R.FUS | 9165.33 [8840.42, 9490.24], <0.01 | -115.02 [-269.60, 39.57], 0.14 | -32.05 [-169.82, 105.72], 0.64 | 816.47 [605.24, 1027.70], <0.01 | -2.16 [-18.80, 14.48], 0.80 | | 1.00 |
| R.IPL | 14732.81 [14167.39, 15298.23], <0.01 | 224.17 [-44.84, 493.19], 0.10 | 90.07 [-149.69, 329.82], 0.46 | 1254.79 [887.20, 1622.38], <0.01 | 25.34 [-3.61, 54.30], 0.09 | | 1.00 |
| R.ITG | 11145.55 [10706.21, 11584.90], <0.01 | 1.69 [-207.34, 210.72], 0.99 | 47.03 [-139.27, 233.33], 0.62 | 981.05 [695.42, 1266.67], <0.01 | 17.68 [-4.82, 40.18], 0.12 | | 1.00 |
| R.iCC | 2493.79 [2383.01, 2604.57], <0.01 | -7.90 [-60.60, 44.81], 0.77 | 30.33 [-16.64, 77.30], 0.20 | 164.42 [92.40, 236.44], <0.01 | 2.25 [-3.42, 7.93], 0.43 | | 1.00 |
| R.LOG | 11586.01 [11123.73, 12048.28], <0.01 | 118.31 [-101.63, 338.26], 0.29 | -90.49 [-286.51, 105.54], 0.36 | 569.81 [269.28, 870.35], <0.01 | -2.09 [-25.77, 21.58], 0.86 | | 1.00 |
| R.LOF | 7859.05 [7547.20, 8170.90], <0.01 | -41.74 [-190.11, 106.63], 0.58 | -47.61 [-179.85, 84.62], 0.48 | 626.90 [424.16, 829.64], <0.01 | 6.46 [-9.51, 22.44], 0.42 | | 1.00 |
| R.LING | 6665.29 [6341.50, 6989.07], <0.01 | -122.99 [-277.04, 31.06], 0.12 | -87.58 [-224.87, 49.72], 0.21 | 302.73 [92.24, 513.23], <0.01 | -13.47 [-30.05, 3.12], 0.11 | | 1.00 |
| R.MOF | 5847.39 [5683.12, 6011.66], <0.01 | -21.09 [-99.25, 57.06], 0.59 | -27.74 [-97.39, 41.92], 0.43 | 452.01 [345.22, 558.81], <0.01 | 5.86 [-2.55, 14.27], 0.17 | | 1.00 |
| R.MTG | 12760.77 [12320.01, 13201.54], <0.01 | -150.38 [-360.09, 59.32], 0.16 | -104.37 [-291.26, 82.53], 0.27 | 798.69 [512.14, 1085.24], <0.01 | -3.06 [-25.64, 19.51], 0.79 | | 1.00 |
| R.PARH | 2039.36 [1956.45, 2122.28], <0.01 | -29.38 [-68.83, 10.07], 0.14 | 8.89 [-26.27, 44.05], 0.62 | 82.56 [28.65, 136.47], <0.01 | -1.15 [-5.40, 3.09], 0.59 | | 1.00 |
| R.paraC | 3747.44 [3584.08, 3910.79], <0.01 | 50.09 [-27.63, 127.81], 0.20 | 21.56 [-47.71, 90.83], 0.54 | 226.36 [120.16, 332.56], <0.01 | -1.92 [-10.28, 6.45], 0.65 | | 1.00 |
| R.pOPER | 4234.36 [4043.83, 4424.88], <0.01 | -22.11 [-112.75, 68.54], 0.63 | -86.20 [-166.99, -5.41], 0.04 | 379.24 [255.38, 503.11], <0.01 | -1.31 [-11.07, 8.45], 0.79 | | 1.00 |
| R.pORB | 3124.53 [2957.30, 3291.76], <0.01 | -23.92 [-103.48, 55.64], 0.55 | -41.00 [-111.91, 29.91], 0.25 | 245.81 [137.09, 354.52], <0.01 | 2.33 [-6.24, 10.89], 0.59 | | 1.00 |
| R.pTRI | 4486.22 [4244.84, 4727.59], <0.01 | 31.50 [-83.34, 146.34], 0.59 | -44.85 [-147.20, 57.50], 0.39 | 438.49 [281.57, 595.41], <0.01 | -10.49 [-22.86, 1.87], 0.10 | | 1.00 |
| R.periCAL | 1991.07 [1840.68, 2141.45], <0.01 | -10.52 [-82.07, 61.03], 0.77 | 10.79 [-52.98, 74.56], 0.74 | 123.36 [25.59, 221.13], 0.01 | -0.23 [-7.94, 7.47], 0.95 | | 1.00 |
| R.postC | 9037.76 [8686.60, 9388.91], <0.01 | -71.08 [-238.15, 95.99], 0.40 | 107.58 [-41.32, 256.48], 0.15 | 797.07 [568.77, 1025.36], <0.01 | 3.11 [-14.88, 21.09], 0.73 | | 1.00 |
| R.PCC | 3375.58 [3240.26, 3510.89], <0.01 | -48.11 [-112.48, 16.27], 0.14 | -37.69 [-95.07, 19.68], 0.20 | 276.11 [188.14, 364.08], <0.01 | 0.35 [-6.58, 7.28], 0.92 | | 1.00 |
| R.preC | 13024.20 [12650.35, 13398.04], <0.01 | -94.37 [-272.23, 83.50], 0.29 | 73.34 [-85.18, 231.86], 0.36 | 591.34 [348.30, 834.39], <0.01 | 10.93 [-8.21, 30.08], 0.26 | | 1.00 |
| R.PCUN | 9843.13 [9509.71, 10176.54], <0.01 | 113.26 [-45.37, 271.89], 0.16 | 77.53 [-63.85, 218.91], 0.28 | 852.49 [635.73, 1069.25], <0.01 | 16.77 [-0.30, 33.85], 0.05 | | 1.00 |
| R.rACC | 2130.58 [1990.65, 2270.50], <0.01 | 9.01 [-57.57, 75.58], 0.79 | -2.44 [-61.77, 56.90], 0.94 | 229.73 [138.76, 320.70], <0.01 | 2.79 [-4.38, 9.95], 0.44 | | 1.00 |
| R.rMFG | 17562.19 [16995.35, 18129.03], <0.01 | -178.80 [-448.48, 90.89], 0.19 | -221.72 [-462.08, 18.64], 0.07 | 1637.29 [1268.78, 2005.80], <0.01 | -16.80 [-45.83, 12.23], 0.25 | | 1.00 |
| R.SFG | 23748.17 [23039.05, 24457.29], <0.01 | 106.68 [-230.70, 444.06], 0.53 | 41.20 [-259.49, 341.89], 0.79 | 1350.51 [889.50, 1811.52], <0.01 | 29.16 [-7.16, 65.47], 0.11 | | 1.00 |
| R.SPL | 12778.69 [12245.27, 13312.11], <0.01 | 13.33 [-240.46, 267.11], 0.92 | 87.16 [-139.03, 313.35], 0.45 | 992.91 [646.12, 1339.70], <0.01 | 11.98 [-15.34, 39.30], 0.39 | | 1.00 |
| R.STG | 12056.02 [11624.70, 12487.34], <0.01 | -72.82 [-278.03, 132.39], 0.48 | -83.20 [-266.10, 99.69], 0.37 | 885.54 [605.13, 1165.95], <0.01 | -6.20 [-28.29, 15.89], 0.58 | | 1.00 |
| R.SMAR | 10663.86 [10245.39, 11082.34], <0.01 | -88.72 [-287.81, 110.38], 0.38 | 18.59 [-158.86, 196.03], 0.84 | 872.60 [600.55, 1144.66], <0.01 | -0.68 [-22.12, 20.75], 0.95 | | 1.00 |
| R.FP | 1364.97 [1294.22, 1435.73], <0.01 | -9.42 [-43.08, 24.25], 0.58 | -43.33 [-73.34, -13.33], <0.01 | 40.34 [-5.66, 86.34], 0.08 | 0.58 [-3.04, 4.21], 0.75 | | 1.00 |
| R.TP | 2375.79 [2239.22, 2512.36], <0.01 | 7.99 [-56.99, 72.96], 0.81 | 6.37 [-51.54, 64.28], 0.83 | 54.95 [-33.84, 143.73], 0.22 | 5.45 [-1.55, 12.44], 0.13 | | 1.00 |
| R.TT | 897.70 [852.08, 943.31], <0.01 | -19.49 [-41.19, 2.22], 0.08 | -6.18 [-25.53, 13.16], 0.53 | 68.26 [38.60, 97.92], <0.01 | -2.17 [-4.51, 0.17], 0.07 | | 0.99 |
| R.INS | 7346.78 [7102.15, 7591.40], <0.01 | -104.89 [-221.27, 11.50], 0.08 | 22.09 [-81.64, 125.82], 0.67 | 451.98 [292.94, 611.01], <0.01 | 4.08 [-8.44, 16.61], 0.52 | | 0.99 |
| L.THAL | 7988.42 [7746.40, 8230.44], <0.01 | -19.35 [-134.49, 95.80], 0.74 | 13.04 [-89.58, 115.67], 0.80 | 637.14 [479.80, 794.48], <0.01 | 10.87 [-1.52, 23.26], 0.08 | | 1.00 |
| R.THAL | 7492.49 [7268.47, 7716.52], <0.01 | -39.82 [-146.40, 66.77], 0.46 | -28.19 [-123.19, 66.80], 0.56 | 518.91 [373.27, 664.55], <0.01 | 7.40 [-4.08, 18.87], 0.20 | | 1.00 |
| L.PUT | 4075.86 [3882.72, 4269.00], <0.01 | 92.15 [0.26, 184.04], 0.05 | -5.52 [-87.42, 76.38], 0.89 | 266.73 [141.17, 392.30], <0.01 | -3.51 [-13.40, 6.38], 0.48 | | 0.94 |
| R.PUT | 4171.09 [3965.95, 4376.22], <0.01 | 103.82 [6.22, 201.42], 0.04 | 13.71 [-73.27, 100.70], 0.75 | 255.11 [121.74, 388.47], <0.01 | -4.27 [-14.78, 6.24], 0.42 | | 0.89 |
| L.PALL | 1816.24 [1724.85, 1907.63], <0.01 | 3.65 [-39.83, 47.13], 0.87 | 21.03 [-17.72, 59.79], 0.28 | 144.05 [84.63, 203.47], <0.01 | -0.08 [-4.76, 4.60], 0.97 | | 1.00 |
| R.PALL | 1696.61 [1614.36, 1778.86], <0.01 | 40.36 [1.23, 79.50], 0.04 | -8.02 [-42.90, 26.86], 0.65 | 130.51 [77.04, 183.98], <0.01 | -0.96 [-5.18, 3.25], 0.65 | | 0.92 |
| L.CAUD | 3426.53 [3299.68, 3553.38], <0.01 | -45.87 [-106.22, 14.48], 0.13 | -50.38 [-104.17, 3.41], 0.07 | 273.89 [191.42, 356.36], <0.01 | -3.67 [-10.16, 2.83], 0.27 | | 1.00 |
| R.CAUD | 3405.32 [3281.42, 3529.23], <0.01 | -14.42 [-73.37, 44.53], 0.63 | -51.76 [-104.31, 0.78], 0.05 | 279.43 [198.88, 359.99], <0.01 | -2.90 [-9.24, 3.45], 0.37 | | 1.00 |
| L.HIPP | 4048.86 [3948.37, 4149.35], <0.01 | -26.00 [-73.81, 21.81], 0.28 | 7.10 [-35.51, 49.71], 0.74 | 236.95 [171.62, 302.28], <0.01 | 4.96 [-0.19, 10.11], 0.06 | | 1.00 |
| R.HIPP | 3967.47 [3864.04, 4070.90], <0.01 | -3.32 [-52.53, 45.89], 0.89 | 15.32 [-28.54, 59.18], 0.49 | 217.85 [150.61, 285.10], <0.01 | -0.61 [-5.91, 4.69], 0.82 | | 1.00 |
| L.AMYG | 1532.63 [1476.79, 1588.46], <0.01 | -14.57 [-41.14, 11.99], 0.28 | 16.50 [-7.18, 40.17], 0.17 | 84.30 [48.00, 120.60], <0.01 | 0.47 [-2.39, 3.33], 0.74 | | 1.00 |
| R.AMYG | 1635.96 [1583.69, 1688.22], <0.01 | -8.31 [-33.18, 16.56], 0.51 | -2.72 [-24.89, 19.44], 0.81 | 107.26 [73.28, 141.24], <0.01 | 1.83 [-0.85, 4.50], 0.18 | | 1.00 |
| L.AC | 475.63 [428.81, 522.45], <0.01 | -4.66 [-26.93, 17.62], 0.68 | 10.57 [-9.28, 30.43], 0.29 | 39.57 [9.13, 70.01], 0.01 | 2.70 [0.30, 5.10], 0.03 | | 1.00 |
| R.AC | 532.35 [502.45, 562.26], <0.01 | -5.58 [-19.81, 8.65], 0.44 | 5.92 [-6.76, 18.60], 0.36 | 36.18 [16.74, 55.62], <0.01 | 0.51 [-1.02, 2.04], 0.51 | | 1.00 |
| Shown are parameter estimates with 95% confidence intervals | | | | | | | |
| PvalCor = Multiple comparison corrected p-values *Notes:* STiP-5.1=Semi-structured Interview for Personality Functioning DSM-5; GMV=grey matter volume; ICV=Estimated Intracranial Volume; IQ=Intelligence Quotient | | | | | | | |

**Table S8.** *Regression analysis results of STiP-5.1 Self-direction on individual brain region GMV (N=93)*

|  | **(Intercept)** | **STiP-5.1 Self-direction** | **Age** | **ICV** | **IQ** | **PvalCor** |
| --- | --- | --- | --- | --- | --- | --- |
| L.BSTS | 2525.35 [2386.19, 2664.50], <0.01 | -38.85 [-136.36, 58.66], 0.43 | -17.38 [-78.39, 43.63], 0.57 | 254.77 [160.39, 349.15], <0.01 | -4.52 [-12.01, 2.96], 0.23 | 1.00 |
| L.cACC | 1869.33 [1712.77, 2025.89], <0.01 | -4.48 [-114.19, 105.22], 0.94 | 13.11 [-55.54, 81.75], 0.71 | 173.72 [67.53, 279.91], <0.01 | -0.14 [-8.56, 8.28], 0.97 | 1.00 |
| L.cMFG | 6926.30 [6585.30, 7267.30], <0.01 | -116.34 [-355.29, 122.61], 0.34 | -170.94 [-320.44, -21.43], 0.03 | 241.98 [10.70, 473.26], 0.04 | -1.49 [-19.83, 16.85], 0.87 | 1.00 |
| L.CUN | 2916.84 [2737.91, 3095.76], <0.01 | -69.53 [-194.91, 55.85], 0.27 | 5.32 [-73.12, 83.77], 0.89 | 218.53 [97.18, 339.89], <0.01 | 0.20 [-9.43, 9.82], 0.97 | 1.00 |
| L.ENT | 1997.22 [1847.26, 2147.17], <0.01 | -53.40 [-158.47, 51.68], 0.32 | 4.66 [-61.09, 70.40], 0.89 | 64.53 [-37.18, 166.23], 0.21 | 0.37 [-7.70, 8.43], 0.93 | 1.00 |
| L.FUS | 9498.80 [9185.79, 9811.82], <0.01 | -99.73 [-319.07, 119.60], 0.37 | 94.97 [-42.27, 232.20], 0.17 | 738.74 [526.45, 951.04], <0.01 | 11.82 [-5.02, 28.66], 0.17 | 1.00 |
| L.IPL | 12436.49 [11943.50, 12929.49], <0.01 | -50.13 [-395.58, 295.33], 0.77 | 6.75 [-209.40, 222.89], 0.95 | 996.13 [661.76, 1330.49], <0.01 | 2.01 [-24.51, 28.53], 0.88 | 1.00 |
| L.ITG | 11376.74 [10850.47, 11903.01], <0.01 | -128.40 [-497.17, 240.38], 0.49 | -43.57 [-274.30, 187.16], 0.71 | 811.63 [454.69, 1168.57], <0.01 | 2.88 [-25.43, 31.19], 0.84 | 1.00 |
| L.iCC | 2609.38 [2523.15, 2695.61], <0.01 | -18.95 [-79.38, 41.47], 0.53 | -21.21 [-59.01, 16.60], 0.27 | 175.31 [116.83, 233.79], <0.01 | 2.83 [-1.81, 7.47], 0.23 | 1.00 |
| L.LOG | 11659.84 [11245.38, 12074.31], <0.01 | -76.98 [-367.41, 213.44], 0.60 | 19.56 [-162.16, 201.27], 0.83 | 691.24 [410.13, 972.34], <0.01 | -6.67 [-28.96, 15.62], 0.55 | 1.00 |
| L.LOF | 7916.07 [7628.35, 8203.80], <0.01 | -4.28 [-205.90, 197.33], 0.97 | 26.93 [-99.22, 153.07], 0.67 | 499.68 [304.54, 694.83], <0.01 | 16.82 [1.35, 32.30], 0.03 | 1.00 |
| L.LING | 6095.26 [5813.48, 6377.03], <0.01 | -157.39 [-354.84, 40.06], 0.12 | -77.16 [-200.70, 46.37], 0.22 | 321.98 [130.87, 513.09], <0.01 | 1.26 [-13.89, 16.42], 0.87 | 1.00 |
| L.MOF | 5641.49 [5427.77, 5855.22], <0.01 | 44.97 [-104.79, 194.74], 0.55 | -41.59 [-135.30, 52.11], 0.38 | 459.72 [314.76, 604.67], <0.01 | 5.95 [-5.55, 17.44], 0.31 | 1.00 |
| L.MTG | 11340.41 [10878.03, 11802.79], <0.01 | 25.39 [-298.61, 349.40], 0.88 | -107.64 [-310.36, 95.08], 0.29 | 913.89 [600.28, 1227.49], <0.01 | -13.88 [-38.75, 10.99], 0.27 | 1.00 |
| L.PARH | 2113.09 [2024.35, 2201.83], <0.01 | -3.30 [-65.48, 58.88], 0.92 | -10.67 [-49.57, 28.24], 0.59 | 55.39 [-4.80, 115.57], 0.07 | 6.20 [1.43, 10.97], 0.01 | 1.00 |
| L.paraC | 3486.02 [3351.57, 3620.46], <0.01 | -5.99 [-100.20, 88.21], 0.90 | 5.67 [-53.27, 64.61], 0.85 | 210.50 [119.32, 301.69], <0.01 | 3.44 [-3.79, 10.67], 0.35 | 1.00 |
| L.pOPER | 4936.61 [4724.61, 5148.60], <0.01 | 110.10 [-38.45, 258.64], 0.14 | -111.80 [-204.74, -18.85], 0.02 | 405.45 [261.67, 549.23], <0.01 | 3.10 [-8.30, 14.51], 0.59 | 1.00 |
| L.pORB | 2660.61 [2526.42, 2794.81], <0.01 | 0.67 [-93.36, 94.71], 0.99 | 14.04 [-44.80, 72.87], 0.64 | 227.21 [136.19, 318.23], <0.01 | 4.64 [-2.58, 11.86], 0.21 | 1.00 |
| L.pTRI | 4057.14 [3859.14, 4255.14], <0.01 | 6.46 [-132.29, 145.20], 0.93 | -2.11 [-88.92, 84.70], 0.96 | 395.26 [260.97, 529.55], <0.01 | 5.64 [-5.01, 16.29], 0.30 | 1.00 |
| L.periCAL | 1858.46 [1726.87, 1990.06], <0.01 | -51.26 [-143.47, 40.96], 0.27 | 3.43 [-54.27, 61.12], 0.91 | 116.28 [27.02, 205.53], 0.01 | -0.03 [-7.11, 7.04], 0.99 | 1.00 |
| L.postC | 9302.92 [8946.71, 9659.13], <0.01 | -29.40 [-279.00, 220.21], 0.82 | 103.13 [-53.04, 259.30], 0.19 | 742.44 [500.85, 984.04], <0.01 | 1.50 [-17.66, 20.66], 0.88 | 1.00 |
| L.PCC | 3185.99 [3046.36, 3325.62], <0.01 | -1.95 [-99.79, 95.89], 0.97 | 31.23 [-29.99, 92.44], 0.31 | 218.92 [124.22, 313.63], <0.01 | 6.28 [-1.23, 13.79], 0.10 | 1.00 |
| L.preC | 13398.53 [13020.13, 13776.93], <0.01 | -173.16 [-438.32, 91.99], 0.20 | -78.88 [-244.78, 87.02], 0.35 | 748.74 [492.10, 1005.39], <0.01 | 0.19 [-20.16, 20.54], 0.99 | 1.00 |
| L.PCUN | 9693.10 [9388.14, 9998.05], <0.01 | -38.88 [-252.57, 174.81], 0.72 | 95.85 [-37.85, 229.55], 0.16 | 766.55 [559.72, 973.38], <0.01 | 8.72 [-7.68, 25.12], 0.29 | 1.00 |
| L.rACC | 2772.72 [2617.53, 2927.92], <0.01 | -51.46 [-160.21, 57.29], 0.35 | 62.73 [-5.31, 130.78], 0.07 | 219.67 [114.41, 324.93], <0.01 | 5.22 [-3.12, 13.57], 0.22 | 1.00 |
| L.rMFG | 17460.30 [16944.46, 17976.15], <0.01 | -118.13 [-479.60, 243.34], 0.52 | -105.21 [-331.37, 120.95], 0.36 | 1617.57 [1267.71, 1967.44], <0.01 | -2.42 [-30.17, 25.32], 0.86 | 1.00 |
| L.SFG | 24968.14 [24289.30, 25646.97], <0.01 | -7.81 [-483.49, 467.87], 0.97 | -2.00 [-299.62, 295.62], 0.99 | 1848.85 [1388.44, 2309.26], <0.01 | 23.49 [-13.02, 60.01], 0.20 | 1.00 |
| L.SPL | 13142.12 [12628.13, 13656.11], <0.01 | -67.04 [-427.21, 293.13], 0.71 | 126.65 [-98.70, 352.00], 0.27 | 915.28 [566.67, 1263.88], <0.01 | -0.21 [-27.86, 27.44], 0.99 | 1.00 |
| L.STG | 12530.93 [12122.53, 12939.32], <0.01 | -32.90 [-319.08, 253.27], 0.82 | 29.05 [-150.00, 208.10], 0.75 | 887.82 [610.83, 1164.81], <0.01 | -3.30 [-25.27, 18.67], 0.77 | 1.00 |
| L.SMAR | 11763.96 [11204.45, 12323.46], <0.01 | 61.25 [-330.81, 453.30], 0.76 | -11.86 [-257.16, 233.44], 0.92 | 1204.01 [824.53, 1583.48], <0.01 | 9.60 [-20.50, 39.69], 0.53 | 1.00 |
| L.FP | 1113.97 [1061.39, 1166.55], <0.01 | 12.98 [-23.86, 49.83], 0.49 | -19.91 [-42.96, 3.14], 0.09 | 24.60 [-11.07, 60.26], 0.17 | 0.96 [-1.87, 3.78], 0.50 | 1.00 |
| L.TP | 2501.49 [2331.75, 2671.23], <0.01 | -96.58 [-215.52, 22.36], 0.11 | 33.70 [-40.72, 108.12], 0.37 | 21.81 [-93.32, 136.93], 0.71 | 1.55 [-7.58, 10.68], 0.74 | 1.00 |
| L.TT | 1087.84 [1029.10, 1146.58], <0.01 | -17.54 [-58.70, 23.62], 0.40 | 9.03 [-16.73, 34.78], 0.49 | 59.31 [19.46, 99.15], <0.01 | -0.84 [-4.00, 2.32], 0.60 | 1.00 |
| L.INS | 7273.52 [7070.79, 7476.26], <0.01 | -76.67 [-218.73, 65.40], 0.29 | 29.87 [-59.01, 118.76], 0.51 | 378.86 [241.35, 516.36], <0.01 | 5.49 [-5.42, 16.39], 0.32 | 1.00 |
| R.BSTS | 2391.87 [2281.17, 2502.58], <0.01 | -35.31 [-112.88, 42.27], 0.37 | -16.10 [-64.64, 32.43], 0.51 | 169.37 [94.28, 244.45], <0.01 | -0.90 [-6.86, 5.05], 0.76 | 1.00 |
| R.cACC | 2085.46 [1937.25, 2233.67], <0.01 | -17.48 [-121.34, 86.37], 0.74 | -15.86 [-80.84, 49.12], 0.63 | 229.45 [128.92, 329.97], <0.01 | 0.82 [-7.16, 8.79], 0.84 | 1.00 |
| R.cMFG | 6852.24 [6536.25, 7168.23], <0.01 | -214.28 [-435.70, 7.14], 0.06 | -29.12 [-167.66, 109.41], 0.68 | 483.54 [269.23, 697.85], <0.01 | 12.04 [-4.96, 29.03], 0.16 | 0.97 |
| R.CUN | 3218.77 [3031.02, 3406.52], <0.01 | -78.19 [-209.75, 53.37], 0.24 | -17.49 [-99.80, 64.82], 0.67 | 161.04 [33.70, 288.38], 0.01 | -4.27 [-14.37, 5.83], 0.40 | 1.00 |
| R.ENT | 1893.57 [1767.27, 2019.88], <0.01 | -59.05 [-147.55, 29.46], 0.19 | -27.60 [-82.98, 27.77], 0.32 | 79.79 [-5.87, 165.45], 0.07 | 1.68 [-5.12, 8.47], 0.63 | 1.00 |
| R.FUS | 9106.84 [8792.45, 9421.24], <0.01 | -120.55 [-340.86, 99.76], 0.28 | -25.05 [-162.89, 112.79], 0.72 | 823.77 [610.54, 1037.01], <0.01 | -2.40 [-19.31, 14.51], 0.78 | 1.00 |
| R.IPL | 14924.39 [14374.15, 15474.63], <0.01 | 164.63 [-220.94, 550.20], 0.40 | 72.26 [-168.98, 313.50], 0.55 | 1224.14 [850.94, 1597.33], <0.01 | 24.81 [-4.79, 54.40], 0.10 | 1.00 |
| R.ITG | 11211.66 [10789.26, 11634.07], <0.01 | -57.38 [-353.37, 238.61], 0.70 | 43.43 [-141.76, 228.63], 0.64 | 967.13 [680.64, 1253.62], <0.01 | 16.84 [-5.88, 39.56], 0.14 | 1.00 |
| R.iCC | 2499.37 [2392.84, 2605.90], <0.01 | -16.97 [-91.62, 57.67], 0.65 | 30.30 [-16.41, 77.00], 0.20 | 162.89 [90.64, 235.14], <0.01 | 2.11 [-3.62, 7.84], 0.47 | 1.00 |
| R.LOG | 11656.11 [11209.76, 12102.47], <0.01 | 115.00 [-197.78, 427.77], 0.47 | -98.22 [-293.92, 97.47], 0.32 | 560.20 [257.46, 862.93], <0.01 | -1.97 [-25.98, 22.04], 0.87 | 1.00 |
| R.LOF | 7807.78 [7507.21, 8108.35], <0.01 | -16.51 [-227.13, 194.11], 0.88 | -43.46 [-175.24, 88.32], 0.51 | 635.91 [432.05, 839.77], <0.01 | 6.77 [-9.40, 22.93], 0.41 | 1.00 |
| R.LING | 6657.10 [6345.74, 6968.46], <0.01 | -178.19 [-396.37, 39.99], 0.11 | -83.00 [-219.51, 53.51], 0.23 | 299.04 [87.87, 510.22], <0.01 | -14.43 [-31.18, 2.31], 0.09 | 1.00 |
| R.MOF | 5862.22 [5704.49, 6019.95], <0.01 | -45.28 [-155.80, 65.25], 0.42 | -27.82 [-96.97, 41.33], 0.43 | 447.94 [340.96, 554.92], <0.01 | 5.48 [-3.00, 13.97], 0.20 | 1.00 |
| R.MTG | 12807.19 [12385.97, 13228.40], <0.01 | -269.02 [-564.17, 26.14], 0.07 | -101.79 [-286.46, 82.88], 0.28 | 782.24 [496.56, 1067.92], <0.01 | -4.98 [-27.64, 17.67], 0.66 | 0.99 |
| R.PARH | 2009.41 [1928.80, 2090.01], <0.01 | -17.18 [-73.66, 39.31], 0.55 | 11.48 [-23.86, 46.82], 0.52 | 87.60 [32.93, 142.27], <0.01 | -1.02 [-5.36, 3.31], 0.64 | 1.00 |
| R.paraC | 3745.56 [3588.64, 3902.48], <0.01 | 77.29 [-32.66, 187.25], 0.17 | 19.97 [-48.82, 88.77], 0.57 | 228.96 [122.54, 335.39], <0.01 | -1.46 [-9.90, 6.98], 0.73 | 1.00 |
| R.pOPER | 4261.51 [4078.76, 4444.25], <0.01 | -57.97 [-186.03, 70.08], 0.37 | -86.91 [-167.03, -6.79], 0.03 | 372.52 [248.58, 496.46], <0.01 | -1.85 [-11.68, 7.97], 0.71 | 1.00 |
| R.pORB | 3093.74 [2932.51, 3254.96], <0.01 | -8.18 [-121.16, 104.79], 0.89 | -38.55 [-109.23, 32.14], 0.28 | 251.27 [141.92, 360.61], <0.01 | 2.52 [-6.15, 11.19], 0.57 | 1.00 |
| R.pTRI | 4543.85 [4311.20, 4776.50], <0.01 | -4.71 [-167.73, 158.32], 0.95 | -49.00 [-151.00, 53.00], 0.34 | 427.68 [269.89, 585.47], <0.01 | -10.97 [-23.48, 1.54], 0.08 | 1.00 |
| R.periCAL | 2009.47 [1865.03, 2153.92], <0.01 | -32.56 [-133.78, 68.66], 0.52 | 10.16 [-53.17, 73.49], 0.75 | 119.00 [21.03, 216.97], 0.02 | -0.57 [-8.34, 7.20], 0.89 | 1.00 |
| R.postC | 9011.64 [8673.31, 9349.96], <0.01 | -83.59 [-320.66, 153.49], 0.49 | 111.37 [-36.96, 259.70], 0.14 | 799.46 [570.00, 1028.93], <0.01 | 2.83 [-15.37, 21.03], 0.76 | 1.00 |
| R.PCC | 3381.27 [3251.56, 3510.98], <0.01 | -77.76 [-168.65, 13.13], 0.09 | -36.38 [-93.25, 20.49], 0.21 | 272.78 [184.81, 360.76], <0.01 | -0.15 [-7.12, 6.83], 0.97 | 1.00 |
| R.preC | 13032.42 [12673.27, 13391.57], <0.01 | -149.87 [-401.53, 101.80], 0.24 | 76.08 [-81.38, 233.54], 0.34 | 585.44 [341.86, 829.03], <0.01 | 10.00 [-9.32, 29.32], 0.31 | 1.00 |
| R.PCUN | 9984.52 [9660.30, 10308.74], <0.01 | 42.74 [-184.45, 269.93], 0.71 | 66.15 [-76.00, 208.30], 0.36 | 827.57 [607.67, 1047.47], <0.01 | 15.92 [-1.52, 33.36], 0.07 | 1.00 |
| R.rACC | 2169.57 [2035.04, 2304.11], <0.01 | -21.76 [-116.04, 72.51], 0.65 | -4.83 [-63.81, 54.16], 0.87 | 221.88 [130.63, 313.13], <0.01 | 2.36 [-4.88, 9.59], 0.52 | 1.00 |
| R.rMFG | 17553.48 [17008.41, 18098.55], <0.01 | -261.93 [-643.88, 120.01], 0.18 | -215.23 [-454.20, 23.74], 0.08 | 1631.25 [1261.57, 2000.94], <0.01 | -18.25 [-47.57, 11.07], 0.22 | 1.00 |
| R.SFG | 23662.07 [22982.02, 24342.12], <0.01 | 239.04 [-237.49, 715.57], 0.32 | 42.21 [-255.94, 340.36], 0.78 | 1373.43 [912.20, 1834.67], <0.01 | 31.21 [-5.37, 67.79], 0.09 | 1.00 |
| R.SPL | 12831.95 [12318.70, 13345.20], <0.01 | -28.17 [-387.82, 331.47], 0.88 | 83.86 [-141.16, 308.88], 0.46 | 982.23 [634.13, 1330.33], <0.01 | 11.41 [-16.20, 39.01], 0.41 | 1.00 |
| R.STG | 12063.27 [11648.55, 12477.99], <0.01 | -116.46 [-407.07, 174.14], 0.43 | -81.14 [-262.96, 100.68], 0.38 | 880.79 [599.52, 1162.07], <0.01 | -6.93 [-29.24, 15.37], 0.54 | 1.00 |
| R.SMAR | 10634.10 [10230.93, 11037.27], <0.01 | -106.90 [-389.41, 175.61], 0.45 | 23.17 [-153.59, 199.93], 0.80 | 874.99 [601.54, 1148.44], <0.01 | -1.07 [-22.76, 20.61], 0.92 | 1.00 |
| R.FP | 1381.70 [1314.08, 1449.32], <0.01 | -29.38 [-76.76, 18.01], 0.22 | -43.91 [-73.56, -14.26], <0.01 | 36.38 [-9.48, 82.25], 0.12 | 0.28 [-3.36, 3.92], 0.88 | 1.00 |
| R.TP | 2401.57 [2270.15, 2532.98], <0.01 | -11.32 [-103.40, 80.77], 0.81 | 4.73 [-52.89, 62.34], 0.87 | 49.85 [-39.28, 138.97], 0.27 | 5.18 [-1.89, 12.25], 0.15 | 1.00 |
| R.TT | 887.41 [843.15, 931.68], <0.01 | -20.09 [-51.10, 10.93], 0.20 | -4.98 [-24.39, 14.43], 0.61 | 69.58 [39.55, 99.60], <0.01 | -2.21 [-4.59, 0.17], 0.07 | 1.00 |
| R.INS | 7311.67 [7075.10, 7548.24], <0.01 | -126.46 [-292.24, 39.31], 0.13 | 27.50 [-76.22, 131.22], 0.60 | 454.78 [294.33, 615.24], <0.01 | 3.62 [-9.10, 16.35], 0.57 | 1.00 |
| L.THAL | 8010.63 [7778.08, 8243.18], <0.01 | -49.33 [-212.28, 113.63], 0.55 | 12.50 [-89.45, 114.46], 0.81 | 631.59 [473.86, 789.31], <0.01 | 10.41 [-2.10, 22.92], 0.10 | 1.00 |
| R.THAL | 7503.20 [7287.98, 7718.41], <0.01 | -69.79 [-220.59, 81.01], 0.36 | -27.42 [-121.78, 66.93], 0.57 | 514.89 [368.93, 660.86], <0.01 | 6.91 [-4.67, 18.49], 0.24 | 1.00 |
| L.PUT | 4094.47 [3908.10, 4280.85], <0.01 | 122.19 [-8.41, 252.79], 0.07 | -9.62 [-91.33, 72.09], 0.82 | 266.86 [140.45, 393.26], <0.01 | -2.95 [-12.97, 7.08], 0.56 | 0.98 |
| R.PUT | 4179.48 [3982.22, 4376.74], <0.01 | 149.06 [10.84, 287.29], 0.03 | 9.77 [-76.72, 96.25], 0.82 | 257.91 [124.12, 391.70], <0.01 | -3.47 [-14.08, 7.14], 0.52 | 0.87 |
| L.PALL | 1815.04 [1727.11, 1902.97], <0.01 | 6.60 [-55.02, 68.21], 0.83 | 20.97 [-17.58, 59.53], 0.28 | 144.46 [84.83, 204.10], <0.01 | -0.04 [-4.77, 4.69], 0.99 | 1.00 |
| R.PALL | 1700.76 [1621.61, 1779.91], <0.01 | 57.15 [1.69, 112.62], 0.04 | -9.60 [-44.30, 25.10], 0.58 | 131.41 [77.73, 185.09], <0.01 | -0.66 [-4.92, 3.59], 0.76 | 0.92 |
| L.CAUD | 3419.82 [3297.67, 3541.97], <0.01 | -63.15 [-148.74, 22.44], 0.15 | -48.48 [-102.03, 5.08], 0.08 | 273.29 [190.44, 356.13], <0.01 | -3.98 [-10.55, 2.59], 0.23 | 1.00 |
| R.CAUD | 3404.38 [3285.16, 3523.60], <0.01 | -20.90 [-104.44, 62.64], 0.62 | -51.23 [-103.50, 1.04], 0.05 | 279.00 [198.14, 359.86], <0.01 | -3.01 [-9.43, 3.40], 0.35 | 1.00 |
| L.HIPP | 4042.88 [3946.08, 4139.67], <0.01 | -33.81 [-101.64, 34.02], 0.32 | 8.30 [-34.14, 50.73], 0.70 | 237.07 [171.42, 302.72], <0.01 | 4.81 [-0.40, 10.02], 0.07 | 1.00 |
| R.HIPP | 3962.71 [3863.17, 4062.25], <0.01 | -0.69 [-70.44, 69.06], 0.98 | 15.68 [-27.96, 59.33], 0.48 | 218.71 [151.20, 286.22], <0.01 | -0.58 [-5.93, 4.78], 0.83 | 1.00 |
| L.AMYG | 1528.92 [1475.12, 1582.72], <0.01 | -18.63 [-56.33, 19.06], 0.33 | 17.19 [-6.40, 40.77], 0.15 | 84.44 [47.96, 120.93], <0.01 | 0.39 [-2.50, 3.29], 0.79 | 1.00 |
| R.AMYG | 1646.44 [1596.46, 1696.41], <0.01 | -22.04 [-57.06, 12.98], 0.21 | -3.00 [-24.91, 18.91], 0.79 | 104.68 [70.78, 138.57], <0.01 | 1.62 [-1.07, 4.31], 0.23 | 1.00 |
| L.AC | 478.42 [433.42, 523.43], <0.01 | -9.56 [-41.10, 21.98], 0.55 | 10.58 [-9.15, 30.31], 0.29 | 38.77 [8.25, 69.30], 0.01 | 2.63 [0.20, 5.05], 0.03 | 1.00 |
| R.AC | 531.40 [502.62, 560.19], <0.01 | -7.56 [-27.73, 12.61], 0.46 | 6.16 [-6.46, 18.77], 0.34 | 36.14 [16.61, 55.66], <0.01 | 0.47 [-1.08, 2.02], 0.55 | 1.00 |
| Shown are parameter estimates with 95% confidence intervals | | | | | | |
| PvalCor = Multiple comparison corrected p-values *Notes:* STiP-5.1=Semi-structured Interview for Personality Functioning DSM-5; GMV=grey matter volume; ICV=Estimated Intracranial Volume; IQ=Intelligence Quotient | | | | | | |

**Table S9.** *Regression analysis results of STiP-5.1 Empathy on individual brain region GMV (N=93)*

|  | **(Intercept)** | **STiP-5.1 Empathy** | **Age** | **ICV** | **IQ** | **PvalCor** |
| --- | --- | --- | --- | --- | --- | --- |
| L.BSTS | 2513.97 [2379.72, 2648.22], <0.01 | -41.49 [-174.61, 91.64], 0.54 | -17.32 [-78.56, 43.92], 0.58 | 260.11 [167.61, 352.61], <0.01 | -4.86 [-12.77, 3.05], 0.23 | 1.00 |
| L.cACC | 1813.60 [1663.43, 1963.76], <0.01 | 66.92 [-81.98, 215.82], 0.37 | 16.97 [-51.53, 85.46], 0.62 | 180.79 [77.32, 284.25], <0.01 | 1.38 [-7.47, 10.22], 0.76 | 1.00 |
| L.cMFG | 7082.18 [6761.74, 7402.62], <0.01 | -374.49 [-692.23, -56.74], 0.02 | -184.19 [-330.36, -38.02], 0.01 | 235.44 [14.65, 456.22], 0.04 | -7.93 [-26.81, 10.94], 0.41 | 0.73 |
| L.CUN | 2889.56 [2716.55, 3062.57], <0.01 | -65.12 [-236.68, 106.44], 0.45 | 5.93 [-72.99, 84.85], 0.88 | 228.90 [109.69, 348.11], <0.01 | -0.22 [-10.41, 9.98], 0.97 | 1.00 |
| L.ENT | 2007.32 [1863.32, 2151.32], <0.01 | -90.93 [-233.72, 51.86], 0.21 | 2.92 [-62.76, 68.61], 0.93 | 68.81 [-30.41, 168.02], 0.17 | -0.83 [-9.32, 7.65], 0.85 | 1.00 |
| L.FUS | 9379.56 [9076.60, 9682.51], <0.01 | 12.17 [-288.24, 312.57], 0.94 | 101.51 [-36.69, 239.70], 0.15 | 763.11 [554.38, 971.85], <0.01 | 13.52 [-4.33, 31.36], 0.14 | 1.00 |
| L.IPL | 12575.04 [12102.99, 13047.09], <0.01 | -255.41 [-723.49, 212.67], 0.28 | -4.02 [-219.35, 211.31], 0.97 | 984.84 [659.60, 1310.09], <0.01 | -2.81 [-30.61, 25.00], 0.84 | 1.00 |
| L.ITG | 11512.85 [11010.50, 12015.21], <0.01 | -365.97 [-864.11, 132.17], 0.15 | -55.66 [-284.81, 173.50], 0.63 | 808.67 [462.54, 1154.80], <0.01 | -3.21 [-32.80, 26.38], 0.83 | 1.00 |
| L.iCC | 2595.81 [2512.58, 2679.04], <0.01 | -9.67 [-92.20, 72.86], 0.82 | -20.61 [-58.57, 17.36], 0.28 | 178.86 [121.52, 236.21], <0.01 | 2.89 [-2.01, 7.80], 0.24 | 1.00 |
| L.LOG | 11485.56 [11086.41, 11884.71], <0.01 | 117.75 [-278.05, 513.54], 0.56 | 30.43 [-151.65, 212.50], 0.74 | 719.80 [444.79, 994.82], <0.01 | -3.01 [-26.52, 20.50], 0.80 | 1.00 |
| L.LOF | 7928.35 [7651.18, 8205.52], <0.01 | -22.41 [-297.24, 252.43], 0.87 | 25.98 [-100.45, 152.41], 0.68 | 498.67 [307.70, 689.64], <0.01 | 16.40 [0.07, 32.73], 0.05 | 1.00 |
| L.LING | 5989.71 [5715.06, 6264.35], <0.01 | -89.69 [-362.03, 182.64], 0.51 | -72.69 [-197.97, 52.59], 0.25 | 350.65 [161.41, 539.88], <0.01 | 1.58 [-14.60, 17.76], 0.85 | 1.00 |
| L.MOF | 5570.95 [5367.41, 5774.48], <0.01 | 158.32 [-43.50, 360.15], 0.12 | -35.74 [-128.58, 57.10], 0.45 | 463.47 [323.23, 603.70], <0.01 | 8.73 [-3.26, 20.72], 0.15 | 1.00 |
| L.MTG | 11505.89 [11062.04, 11949.74], <0.01 | -181.13 [-621.25, 258.99], 0.42 | -118.87 [-321.34, 83.60], 0.25 | 891.66 [585.84, 1197.48], <0.01 | -18.18 [-44.32, 7.97], 0.17 | 1.00 |
| L.PARH | 2120.94 [2035.50, 2206.38], <0.01 | -15.14 [-99.86, 69.59], 0.72 | -11.29 [-50.26, 27.69], 0.57 | 54.79 [-4.07, 113.66], 0.07 | 5.92 [0.89, 10.95], 0.02 | 1.00 |
| L.paraC | 3467.12 [3337.63, 3596.62], <0.01 | 16.18 [-112.22, 144.58], 0.80 | 6.89 [-52.18, 65.96], 0.82 | 213.36 [124.14, 302.58], <0.01 | 3.88 [-3.75, 11.50], 0.32 | 1.00 |
| L.pOPER | 4924.83 [4721.52, 5128.14], <0.01 | 175.53 [-26.07, 377.14], 0.09 | -108.86 [-201.61, -16.12], 0.02 | 395.55 [255.47, 535.64], <0.01 | 5.33 [-6.65, 17.30], 0.38 | 0.99 |
| L.pORB | 2735.97 [2608.39, 2863.54], <0.01 | -98.31 [-224.81, 28.19], 0.13 | 8.71 [-49.48, 66.91], 0.77 | 218.21 [130.31, 306.11], <0.01 | 2.50 [-5.02, 10.01], 0.51 | 1.00 |
| L.pTRI | 4025.83 [3835.37, 4216.30], <0.01 | 50.64 [-138.22, 239.50], 0.60 | 0.23 [-86.65, 87.11], 1.00 | 398.31 [267.08, 529.54], <0.01 | 6.64 [-4.58, 17.86], 0.24 | 1.00 |
| L.periCAL | 1862.76 [1736.25, 1989.26], <0.01 | -80.16 [-205.60, 45.29], 0.21 | 2.15 [-55.56, 59.86], 0.94 | 121.03 [33.86, 208.19], <0.01 | -1.03 [-8.49, 6.42], 0.78 | 1.00 |
| L.postC | 9211.73 [8868.84, 9554.62], <0.01 | 77.43 [-262.58, 417.44], 0.65 | 109.02 [-47.39, 265.44], 0.17 | 756.27 [520.02, 992.53], <0.01 | 3.61 [-16.59, 23.80], 0.72 | 1.00 |
| L.PCC | 3175.12 [3040.62, 3309.63], <0.01 | 11.49 [-121.88, 144.86], 0.86 | 31.96 [-29.40, 93.31], 0.30 | 220.41 [127.74, 313.09], <0.01 | 6.56 [-1.36, 14.48], 0.10 | 1.00 |
| L.preC | 13263.55 [12895.85, 13631.25], <0.01 | -73.83 [-438.44, 290.78], 0.69 | -72.63 [-240.35, 95.10], 0.39 | 782.51 [529.17, 1035.86], <0.01 | 1.08 [-20.58, 22.74], 0.92 | 1.00 |
| L.PCUN | 9649.18 [9355.16, 9943.21], <0.01 | 1.34 [-290.21, 292.90], 0.99 | 98.22 [-35.90, 232.34], 0.15 | 775.74 [573.16, 978.33], <0.01 | 9.31 [-8.01, 26.63], 0.29 | 1.00 |
| L.rACC | 2801.99 [2653.68, 2950.29], <0.01 | -113.36 [-260.42, 33.70], 0.13 | 59.68 [-7.97, 127.33], 0.08 | 221.48 [119.30, 323.67], <0.01 | 3.51 [-5.23, 12.24], 0.43 | 1.00 |
| L.rMFG | 17538.27 [17043.56, 18032.98], <0.01 | -274.44 [-764.99, 216.12], 0.27 | -112.99 [-338.65, 112.68], 0.32 | 1620.45 [1279.59, 1961.31], <0.01 | -6.67 [-35.81, 22.47], 0.65 | 1.00 |
| L.SFG | 25020.76 [24366.97, 25674.55], <0.01 | -80.69 [-728.98, 567.61], 0.81 | -5.87 [-304.11, 292.36], 0.97 | 1843.41 [1392.95, 2293.88], <0.01 | 21.86 [-16.65, 60.37], 0.26 | 1.00 |
| L.SPL | 13145.69 [12650.59, 13640.79], <0.01 | -102.15 [-593.09, 388.79], 0.68 | 125.12 [-100.72, 350.96], 0.27 | 921.73 [580.60, 1262.86], <0.01 | -1.46 [-30.62, 27.70], 0.92 | 1.00 |
| L.STG | 12499.81 [12106.23, 12893.38], <0.01 | -6.82 [-397.09, 383.45], 0.97 | 30.63 [-148.91, 210.16], 0.74 | 894.89 [623.71, 1166.06], <0.01 | -2.98 [-26.16, 20.21], 0.80 | 1.00 |
| L.SMAR | 11624.57 [11088.39, 12160.76], <0.01 | 272.67 [-259.00, 804.35], 0.31 | -0.82 [-245.41, 243.76], 0.99 | 1214.25 [844.81, 1583.68], <0.01 | 14.63 [-16.96, 46.21], 0.36 | 1.00 |
| L.FP | 1122.39 [1071.62, 1173.16], <0.01 | 7.78 [-42.57, 58.12], 0.76 | -20.26 [-43.42, 2.90], 0.09 | 22.27 [-12.72, 57.25], 0.21 | 0.94 [-2.05, 3.93], 0.53 | 1.00 |
| L.TP | 2409.55 [2243.66, 2575.43], <0.01 | -19.23 [-183.72, 145.26], 0.82 | 38.37 [-37.30, 114.04], 0.32 | 42.62 [-71.68, 156.92], 0.46 | 2.52 [-7.25, 12.29], 0.61 | 1.00 |
| L.TT | 1068.04 [1011.21, 1124.86], <0.01 | 0.59 [-55.75, 56.94], 0.98 | 10.09 [-15.83, 36.02], 0.44 | 63.45 [24.30, 102.61], <0.01 | -0.57 [-3.92, 2.77], 0.73 | 1.00 |
| L.INS | 7255.11 [7059.40, 7450.82], <0.01 | -87.17 [-281.24, 106.90], 0.37 | 29.71 [-59.56, 118.99], 0.51 | 388.91 [254.06, 523.75], <0.01 | 4.70 [-6.83, 16.23], 0.42 | 1.00 |
| R.BSTS | 2354.52 [2247.37, 2461.68], <0.01 | -2.11 [-108.36, 104.14], 0.97 | -14.13 [-63.01, 34.75], 0.57 | 177.42 [103.59, 251.24], <0.01 | -0.44 [-6.75, 5.87], 0.89 | 1.00 |
| R.cACC | 2097.36 [1954.75, 2239.98], <0.01 | -41.10 [-182.51, 100.32], 0.57 | -17.04 [-82.09, 48.02], 0.60 | 229.83 [131.57, 328.09], <0.01 | 0.18 [-8.22, 8.58], 0.97 | 1.00 |
| R.cMFG | 6688.07 [6377.97, 6998.17], <0.01 | -95.14 [-402.64, 212.35], 0.54 | -21.58 [-163.04, 119.87], 0.76 | 524.99 [311.33, 738.65], <0.01 | 13.05 [-5.21, 31.32], 0.16 | 1.00 |
| R.CUN | 3188.86 [3007.24, 3370.48], <0.01 | -74.25 [-254.34, 105.85], 0.41 | -16.86 [-99.71, 65.98], 0.69 | 172.61 [47.47, 297.75], <0.01 | -4.76 [-15.45, 5.94], 0.38 | 1.00 |
| R.ENT | 1845.54 [1722.74, 1968.34], <0.01 | -22.53 [-144.30, 99.23], 0.71 | -25.33 [-81.34, 30.69], 0.37 | 91.54 [6.93, 176.15], 0.03 | 2.04 [-5.20, 9.27], 0.58 | 1.00 |
| R.FUS | 9002.53 [8697.71, 9307.36], <0.01 | -37.78 [-340.04, 264.48], 0.80 | -19.96 [-159.01, 119.08], 0.78 | 848.51 [638.49, 1058.54], <0.01 | -1.49 [-19.44, 16.47], 0.87 | 1.00 |
| R.IPL | 14969.15 [14438.26, 15500.04], <0.01 | 180.31 [-346.12, 706.73], 0.50 | 72.23 [-169.94, 314.40], 0.55 | 1201.94 [836.15, 1567.72], <0.01 | 26.35 [-4.92, 57.62], 0.10 | 1.00 |
| R.ITG | 11238.67 [10832.17, 11645.18], <0.01 | -118.99 [-522.08, 284.10], 0.56 | 40.43 [-145.00, 225.86], 0.67 | 969.82 [689.73, 1249.91], <0.01 | 15.08 [-8.86, 39.03], 0.21 | 1.00 |
| R.iCC | 2501.61 [2399.03, 2604.19], <0.01 | -27.62 [-129.34, 74.10], 0.59 | 29.82 [-16.98, 76.61], 0.21 | 164.37 [93.69, 235.05], <0.01 | 1.76 [-4.29, 7.80], 0.57 | 1.00 |
| R.LOG | 11400.83 [10983.13, 11818.54], <0.01 | 503.50 [89.30, 917.70], 0.02 | -77.95 [-268.49, 112.59], 0.42 | 578.66 [290.86, 866.47], <0.01 | 7.29 [-17.32, 31.89], 0.56 | 0.66 |
| R.LOF | 7825.18 [7535.74, 8114.63], <0.01 | -46.94 [-333.95, 240.07], 0.75 | -45.01 [-177.04, 87.02], 0.50 | 635.54 [436.11, 834.97], <0.01 | 5.99 [-11.06, 23.04], 0.49 | 1.00 |
| R.LING | 6686.06 [6388.32, 6983.80], <0.01 | -297.15 [-592.38, -1.91], 0.05 | -88.45 [-224.26, 47.37], 0.20 | 313.89 [108.75, 519.04], <0.01 | -18.31 [-35.85, -0.78], 0.04 | 0.94 |
| R.MOF | 5796.11 [5643.64, 5948.57], <0.01 | 21.30 [-129.88, 172.49], 0.78 | -24.00 [-93.55, 45.55], 0.49 | 460.43 [355.38, 565.48], <0.01 | 6.60 [-2.39, 15.58], 0.15 | 1.00 |
| R.MTG | 12638.84 [12227.12, 13050.56], <0.01 | -169.19 [-577.45, 239.07], 0.41 | -95.00 [-282.80, 92.81], 0.32 | 829.80 [546.13, 1113.48], <0.01 | -4.78 [-29.03, 19.47], 0.70 | 1.00 |
| R.PARH | 1998.77 [1920.98, 2076.55], <0.01 | -10.95 [-88.08, 66.18], 0.78 | 11.90 [-23.58, 47.39], 0.51 | 90.63 [37.03, 144.22], <0.01 | -1.01 [-5.59, 3.57], 0.66 | 1.00 |
| R.paraC | 3772.15 [3620.20, 3924.10], <0.01 | 77.31 [-73.37, 227.98], 0.31 | 19.56 [-49.75, 88.88], 0.58 | 217.88 [113.18, 322.57], <0.01 | -0.89 [-9.84, 8.06], 0.84 | 1.00 |
| R.pOPER | 4209.04 [4032.20, 4385.87], <0.01 | -15.13 [-190.48, 160.22], 0.86 | -84.30 [-164.97, -3.64], 0.04 | 384.69 [262.85, 506.53], <0.01 | -1.35 [-11.77, 9.07], 0.80 | 1.00 |
| R.pORB | 3147.75 [2993.42, 3302.08], <0.01 | -83.06 [-236.09, 69.97], 0.28 | -42.53 [-112.93, 27.87], 0.23 | 245.70 [139.37, 352.04], <0.01 | 0.84 [-8.25, 9.93], 0.86 | 1.00 |
| R.pTRI | 4599.24 [4375.74, 4822.74], <0.01 | -79.82 [-301.44, 141.80], 0.48 | -53.01 [-154.96, 48.94], 0.30 | 421.60 [267.61, 575.59], <0.01 | -12.63 [-25.80, 0.53], 0.06 | 1.00 |
| R.periCAL | 2011.90 [1872.83, 2150.97], <0.01 | -50.52 [-188.42, 87.38], 0.47 | 9.37 [-54.07, 72.80], 0.77 | 122.06 [26.24, 217.88], 0.01 | -1.19 [-9.38, 7.00], 0.77 | 1.00 |
| R.postC | 9032.68 [8707.38, 9357.99], <0.01 | -149.22 [-471.80, 173.35], 0.36 | 108.29 [-40.10, 256.68], 0.15 | 805.54 [581.41, 1029.68], <0.01 | 0.80 [-18.37, 19.96], 0.93 | 1.00 |
| R.PCC | 3368.42 [3243.08, 3493.75], <0.01 | -96.09 [-220.37, 28.19], 0.13 | -36.95 [-94.12, 20.22], 0.20 | 282.29 [195.93, 368.64], <0.01 | -1.11 [-8.49, 6.27], 0.77 | 1.00 |
| R.preC | 12954.14 [12606.24, 13302.03], <0.01 | -114.68 [-459.65, 230.29], 0.51 | 78.76 [-79.93, 237.46], 0.33 | 610.10 [370.40, 849.80], <0.01 | 9.67 [-10.82, 30.16], 0.35 | 1.00 |
| R.PCUN | 9886.72 [9576.79, 10196.66], <0.01 | 190.98 [-116.35, 498.32], 0.22 | 73.89 [-67.49, 215.27], 0.30 | 834.78 [621.23, 1048.33], <0.01 | 19.45 [1.19, 37.70], 0.04 | 1.00 |
| R.rACC | 2209.10 [2080.57, 2337.64], <0.01 | -83.71 [-211.17, 43.74], 0.20 | -8.04 [-66.67, 50.59], 0.79 | 219.42 [130.86, 307.99], <0.01 | 0.86 [-6.72, 8.43], 0.82 | 1.00 |
| R.rMFG | 17643.24 [17123.40, 18163.07], <0.01 | -498.97 [-1014.44, 16.49], 0.06 | -226.58 [-463.71, 10.55], 0.06 | 1647.49 [1289.32, 2005.66], <0.01 | -25.30 [-55.92, 5.32], 0.10 | 0.96 |
| R.SFG | 23736.56 [23079.85, 24393.27], <0.01 | 249.29 [-401.90, 900.49], 0.45 | 41.50 [-258.07, 341.06], 0.78 | 1340.07 [887.59, 1792.55], <0.01 | 33.18 [-5.50, 71.86], 0.09 | 1.00 |
| R.SPL | 12801.11 [12306.56, 13295.65], <0.01 | -0.30 [-490.69, 490.09], 1.00 | 85.51 [-140.08, 311.10], 0.45 | 988.78 [648.03, 1329.53], <0.01 | 11.80 [-17.33, 40.93], 0.42 | 1.00 |
| R.STG | 12031.65 [11631.59, 12431.72], <0.01 | -127.62 [-524.31, 269.08], 0.52 | -81.12 [-263.61, 101.37], 0.38 | 896.49 [620.85, 1172.14], <0.01 | -8.03 [-31.59, 15.54], 0.50 | 1.00 |
| R.SMAR | 10571.67 [10182.31, 10961.03], <0.01 | -73.11 [-459.20, 312.98], 0.71 | 25.55 [-152.06, 203.16], 0.78 | 893.36 [625.09, 1161.63], <0.01 | -1.12 [-24.06, 21.81], 0.92 | 1.00 |
| R.FP | 1366.58 [1301.05, 1432.11], <0.01 | -22.77 [-87.75, 42.21], 0.49 | -43.40 [-73.29, -13.51], <0.01 | 41.19 [-3.96, 86.34], 0.07 | 0.21 [-3.65, 4.07], 0.91 | 1.00 |
| R.TP | 2395.86 [2269.22, 2522.49], <0.01 | -8.93 [-134.50, 116.65], 0.89 | 4.91 [-52.85, 62.68], 0.87 | 51.68 [-35.57, 138.94], 0.24 | 5.15 [-2.31, 12.61], 0.17 | 1.00 |
| R.TT | 880.54 [837.71, 923.38], <0.01 | -20.14 [-62.62, 22.33], 0.35 | -4.87 [-24.41, 14.66], 0.62 | 72.45 [42.94, 101.96], <0.01 | -2.35 [-4.88, 0.17], 0.07 | 1.00 |
| R.INS | 7279.57 [7050.68, 7508.47], <0.01 | -141.52 [-368.49, 85.45], 0.22 | 27.36 [-77.05, 131.77], 0.60 | 471.56 [313.86, 629.27], <0.01 | 2.38 [-11.11, 15.86], 0.73 | 1.00 |
| L.THAL | 8006.79 [7782.73, 8230.84], <0.01 | -66.63 [-288.81, 155.54], 0.55 | 11.84 [-90.37, 114.04], 0.82 | 637.10 [482.73, 791.48], <0.01 | 9.68 [-3.52, 22.87], 0.15 | 1.00 |
| R.THAL | 7468.75 [7260.76, 7676.74], <0.01 | -56.05 [-262.29, 150.20], 0.59 | -26.32 [-121.19, 68.56], 0.58 | 526.14 [382.83, 669.44], <0.01 | 6.70 [-5.55, 18.95], 0.28 | 1.00 |
| L.PUT | 4196.57 [4013.75, 4379.39], <0.01 | 43.08 [-138.21, 224.36], 0.64 | -14.52 [-97.91, 68.88], 0.73 | 242.21 [116.25, 368.18], <0.01 | -3.77 [-14.54, 7.00], 0.49 | 1.00 |
| R.PUT | 4287.43 [4093.14, 4481.73], <0.01 | 74.42 [-118.24, 267.08], 0.44 | 4.96 [-83.66, 93.59], 0.91 | 229.81 [95.94, 363.68], <0.01 | -4.00 [-15.45, 7.44], 0.49 | 1.00 |
| L.PALL | 1845.56 [1761.08, 1930.05], <0.01 | -30.63 [-114.40, 53.15], 0.47 | 18.94 [-19.60, 57.48], 0.33 | 140.17 [81.96, 198.38], <0.01 | -0.79 [-5.77, 4.18], 0.75 | 1.00 |
| R.PALL | 1750.35 [1672.39, 1828.31], <0.01 | 17.74 [-59.57, 95.04], 0.65 | -12.02 [-47.58, 23.54], 0.50 | 119.67 [65.95, 173.38], <0.01 | -1.10 [-5.69, 3.49], 0.63 | 1.00 |
| L.CAUD | 3411.72 [3293.88, 3529.56], <0.01 | -81.11 [-197.96, 35.74], 0.17 | -49.11 [-102.86, 4.65], 0.07 | 280.73 [199.53, 361.92], <0.01 | -4.83 [-11.77, 2.11], 0.17 | 1.00 |
| R.CAUD | 3382.17 [3267.14, 3497.19], <0.01 | -1.11 [-115.17, 112.95], 0.98 | -50.05 [-102.52, 2.41], 0.06 | 283.78 [204.52, 363.03], <0.01 | -2.74 [-9.51, 4.04], 0.42 | 1.00 |
| L.HIPP | 4003.94 [3910.16, 4097.71], <0.01 | 2.17 [-90.82, 95.15], 0.96 | 10.41 [-32.37, 53.18], 0.63 | 245.16 [180.54, 309.77], <0.01 | 5.34 [-0.18, 10.87], 0.06 | 1.00 |
| R.HIPP | 3972.19 [3876.34, 4068.05], <0.01 | -13.50 [-108.55, 81.55], 0.78 | 15.00 [-28.73, 58.73], 0.50 | 217.66 [151.61, 283.71], <0.01 | -0.86 [-6.51, 4.78], 0.76 | 1.00 |
| L.AMYG | 1520.93 [1468.93, 1572.92], <0.01 | -16.55 [-68.11, 35.01], 0.53 | 17.40 [-6.32, 41.11], 0.15 | 87.30 [51.48, 123.13], <0.01 | 0.30 [-2.76, 3.36], 0.85 | 1.00 |
| R.AMYG | 1630.90 [1582.39, 1679.41], <0.01 | -11.57 [-59.67, 36.53], 0.63 | -2.33 [-24.45, 19.80], 0.84 | 108.78 [75.36, 142.20], <0.01 | 1.68 [-1.17, 4.54], 0.24 | 1.00 |
| L.AC | 469.95 [426.51, 513.40], <0.01 | -2.73 [-45.81, 40.35], 0.90 | 11.00 [-8.82, 30.82], 0.27 | 40.76 [10.82, 70.69], <0.01 | 2.70 [0.14, 5.26], 0.04 | 1.00 |
| R.AC | 534.38 [506.75, 562.02], <0.01 | -14.92 [-42.32, 12.48], 0.28 | 5.80 [-6.81, 18.41], 0.36 | 36.56 [17.52, 55.60], <0.01 | 0.26 [-1.37, 1.89], 0.75 | 1.00 |
| Shown are parameter estimates with 95% confidence intervals | | | | | | |
| PvalCor = Multiple comparison corrected p-values *Notes:* STiP-5.1=Semi-structured Interview for Personality Functioning DSM-5; GMV=grey matter volume; ICV=Estimated Intracranial Volume; IQ=Intelligence Quotient | | | | | | |

**Table S10.** *Regression analysis results of STiP-5.1 Intimacy on individual brain region GMV (N=93)*

|  | **(Intercept)** | **STiP-5.1 Intimacy** | **Age** | **ICV** | **IQ** | **PvalCor** | |
| --- | --- | --- | --- | --- | --- | --- | --- |
| L.BSTS | 2499.67 [2378.62, 2620.73], <0.01 | -18.75 [-108.87, 71.36], 0.68 | -16.41 [-77.63, 44.82], 0.60 | 261.58 [169.11, 354.05], <0.01 | -4.19 [-11.64, 3.26], 0.27 | | 1.00 |
| L.cACC | 1869.69 [1733.85, 2005.53], <0.01 | -5.79 [-106.91, 95.33], 0.91 | 12.96 [-55.74, 81.66], 0.71 | 174.07 [70.30, 277.83], <0.01 | -0.14 [-8.50, 8.22], 0.97 | | 1.00 |
| L.cMFG | 6943.59 [6649.59, 7237.58], <0.01 | -158.91 [-377.77, 59.95], 0.15 | -175.22 [-323.91, -26.54], 0.02 | 249.99 [25.41, 474.57], 0.03 | -1.71 [-19.80, 16.38], 0.85 | | 1.00 |
| L.CUN | 2834.63 [2678.31, 2990.94], <0.01 | 6.02 [-110.34, 122.38], 0.92 | 9.85 [-69.20, 88.90], 0.80 | 235.49 [116.08, 354.89], <0.01 | 1.27 [-8.35, 10.89], 0.79 | | 1.00 |
| L.ENT | 1983.55 [1853.45, 2113.66], <0.01 | -49.37 [-146.22, 47.48], 0.31 | 4.34 [-61.46, 70.14], 0.90 | 71.04 [-28.34, 170.43], 0.16 | 0.55 [-7.46, 8.55], 0.89 | | 1.00 |
| L.FUS | 9464.56 [9192.72, 9736.40], <0.01 | -82.69 [-285.05, 119.68], 0.42 | 95.05 [-42.43, 232.53], 0.17 | 752.06 [544.41, 959.71], <0.01 | 12.27 [-4.46, 29.00], 0.15 | | 1.00 |
| L.IPL | 12324.74 [11897.14, 12752.35], <0.01 | 61.61 [-256.71, 379.93], 0.70 | 14.03 [-202.23, 230.29], 0.90 | 1015.25 [688.61, 1341.89], <0.01 | 3.47 [-22.85, 29.78], 0.79 | | 1.00 |
| L.ITG | 11366.61 [10910.56, 11822.66], <0.01 | -143.51 [-483.01, 195.99], 0.40 | -46.06 [-276.71, 184.58], 0.69 | 824.31 [475.94, 1172.68], <0.01 | 3.01 [-25.05, 31.08], 0.83 | | 1.00 |
| L.iCC | 2602.14 [2527.27, 2677.00], <0.01 | -14.91 [-70.64, 40.82], 0.60 | -21.13 [-58.99, 16.73], 0.27 | 177.94 [120.75, 235.12], <0.01 | 2.93 [-1.68, 7.53], 0.21 | | 1.00 |
| L.LOG | 11417.90 [11061.02, 11774.77], <0.01 | 171.35 [-94.31, 437.02], 0.20 | 36.13 [-144.35, 216.62], 0.69 | 729.85 [457.24, 1002.46], <0.01 | -3.52 [-25.48, 18.45], 0.75 | | 1.00 |
| L.LOF | 7955.42 [7706.14, 8204.70], <0.01 | -48.10 [-233.67, 137.47], 0.61 | 23.80 [-102.27, 149.87], 0.71 | 494.89 [304.47, 685.31], <0.01 | 16.31 [0.97, 31.65], 0.04 | | 1.00 |
| L.LING | 5925.28 [5677.34, 6173.23], <0.01 | -3.98 [-188.56, 180.60], 0.97 | -68.15 [-193.55, 57.25], 0.28 | 358.24 [168.83, 547.64], <0.01 | 3.48 [-11.78, 18.73], 0.65 | | 1.00 |
| L.MOF | 5643.52 [5458.28, 5828.75], <0.01 | 51.93 [-85.96, 189.82], 0.46 | -40.60 [-134.28, 53.08], 0.39 | 455.48 [313.98, 596.97], <0.01 | 5.92 [-5.48, 17.32], 0.30 | | 1.00 |
| L.MTG | 11362.90 [10961.65, 11764.15], <0.01 | 6.02 [-292.69, 304.72], 0.97 | -108.71 [-311.64, 94.21], 0.29 | 908.68 [602.18, 1215.19], <0.01 | -14.18 [-38.87, 10.52], 0.26 | | 1.00 |
| L.PARH | 2136.30 [2059.75, 2212.85], <0.01 | -29.30 [-86.28, 27.69], 0.31 | -12.53 [-51.24, 26.18], 0.52 | 52.63 [-5.85, 111.10], 0.08 | 5.90 [1.19, 10.61], 0.01 | | 1.00 |
| L.paraC | 3463.44 [3346.88, 3579.99], <0.01 | 17.43 [-69.34, 104.19], 0.69 | 7.25 [-51.70, 66.19], 0.81 | 214.00 [124.96, 303.04], <0.01 | 3.73 [-3.44, 10.91], 0.30 | | 1.00 |
| L.pOPER | 4992.74 [4807.65, 5177.82], <0.01 | 71.27 [-66.51, 209.06], 0.31 | -113.29 [-206.90, -19.69], 0.02 | 388.35 [246.96, 529.73], <0.01 | 2.37 [-9.02, 13.76], 0.68 | | 1.00 |
| L.pORB | 2686.14 [2569.96, 2802.33], <0.01 | -27.05 [-113.55, 59.44], 0.54 | 12.10 [-46.66, 70.86], 0.68 | 223.79 [135.04, 312.55], <0.01 | 4.30 [-2.85, 11.45], 0.23 | | 1.00 |
| L.pTRI | 4081.22 [3909.49, 4252.95], <0.01 | -18.50 [-146.34, 109.34], 0.77 | -3.79 [-90.64, 83.06], 0.93 | 391.52 [260.34, 522.70], <0.01 | 5.32 [-5.24, 15.89], 0.32 | | 1.00 |
| L.periCAL | 1783.67 [1668.84, 1898.51], <0.01 | 19.91 [-65.57, 105.40], 0.64 | 7.85 [-50.22, 65.93], 0.79 | 130.64 [42.92, 218.36], <0.01 | 0.94 [-6.13, 8.01], 0.79 | | 1.00 |
| L.postC | 9163.20 [8855.83, 9470.57], <0.01 | 117.08 [-111.73, 345.90], 0.31 | 113.08 [-42.37, 268.53], 0.15 | 763.41 [528.62, 998.21], <0.01 | 3.33 [-15.59, 22.24], 0.73 | | 1.00 |
| L.PCC | 3210.47 [3089.60, 3331.34], <0.01 | -29.05 [-119.04, 60.93], 0.52 | 29.30 [-31.83, 90.43], 0.34 | 215.88 [123.55, 308.21], <0.01 | 5.96 [-1.48, 13.40], 0.11 | | 1.00 |
| L.preC | 13257.99 [12926.90, 13589.07], <0.01 | -55.08 [-301.55, 191.39], 0.66 | -72.52 [-239.97, 94.92], 0.39 | 782.52 [529.61, 1035.43], <0.01 | 2.02 [-18.35, 22.40], 0.84 | | 1.00 |
| L.PCUN | 9527.64 [9265.59, 9789.69], <0.01 | 133.75 [-61.33, 328.83], 0.18 | 107.54 [-24.99, 240.07], 0.11 | 791.74 [591.56, 991.92], <0.01 | 10.88 [-5.25, 27.00], 0.18 | | 1.00 |
| L.rACC | 2811.81 [2679.79, 2943.83], <0.01 | -104.61 [-202.89, -6.32], 0.04 | 58.43 [-8.34, 125.20], 0.09 | 219.08 [118.23, 319.93], <0.01 | 4.72 [-3.41, 12.84], 0.25 | | 0.89 |
| L.rMFG | 17426.64 [16978.99, 17874.30], <0.01 | -105.47 [-438.72, 227.78], 0.53 | -105.64 [-332.04, 120.75], 0.36 | 1632.43 [1290.48, 1974.39], <0.01 | -1.98 [-29.53, 25.56], 0.89 | | 1.00 |
| L.SFG | 24917.25 [24328.38, 25506.11], <0.01 | 46.13 [-392.24, 484.51], 0.83 | 1.70 [-296.11, 299.51], 0.99 | 1856.23 [1406.40, 2306.06], <0.01 | 24.16 [-12.08, 60.40], 0.19 | | 1.00 |
| L.SPL | 13083.55 [12637.25, 13529.85], <0.01 | -16.79 [-349.03, 315.45], 0.92 | 129.43 [-96.28, 355.14], 0.26 | 928.90 [587.98, 1269.82], <0.01 | 0.55 [-26.91, 28.02], 0.97 | | 1.00 |
| L.STG | 12497.82 [12143.36, 12852.28], <0.01 | -3.48 [-267.35, 260.39], 0.98 | 30.75 [-148.51, 210.01], 0.73 | 895.08 [624.31, 1165.85], <0.01 | -2.87 [-24.68, 18.94], 0.79 | | 1.00 |
| L.SMAR | 11690.47 [11206.71, 12174.22], <0.01 | 153.92 [-206.20, 514.05], 0.40 | -4.67 [-249.32, 239.98], 0.97 | 1208.26 [838.72, 1577.79], <0.01 | 10.55 [-19.22, 40.32], 0.48 | | 1.00 |
| L.FP | 1113.99 [1068.46, 1159.52], <0.01 | 15.61 [-18.29, 49.50], 0.36 | -19.58 [-42.61, 3.45], 0.09 | 23.45 [-11.34, 58.23], 0.18 | 0.96 [-1.85, 3.76], 0.50 | | 1.00 |
| L.TP | 2413.68 [2264.35, 2563.01], <0.01 | -20.44 [-131.60, 90.73], 0.72 | 37.97 [-37.55, 113.49], 0.32 | 41.89 [-72.18, 155.96], 0.47 | 2.69 [-6.50, 11.88], 0.56 | | 1.00 |
| L.TT | 1068.84 [1017.66, 1120.02], <0.01 | -0.38 [-38.48, 37.71], 0.98 | 10.04 [-15.85, 35.92], 0.44 | 63.35 [24.26, 102.45], <0.01 | -0.59 [-3.74, 2.56], 0.71 | | 1.00 |
| L.INS | 7168.26 [6991.32, 7345.20], <0.01 | 22.58 [-109.14, 154.30], 0.73 | 35.98 [-53.50, 125.47], 0.43 | 399.47 [264.31, 534.63], <0.01 | 6.86 [-4.03, 17.75], 0.21 | | 1.00 |
| R.BSTS | 2342.46 [2246.01, 2438.90], <0.01 | 11.42 [-60.37, 83.22], 0.75 | -13.22 [-61.99, 35.56], 0.59 | 178.98 [105.31, 252.66], <0.01 | -0.26 [-6.19, 5.68], 0.93 | | 1.00 |
| R.cACC | 2063.64 [1934.96, 2192.32], <0.01 | 2.77 [-93.02, 98.56], 0.95 | -14.63 [-79.71, 50.44], 0.66 | 233.86 [135.56, 332.16], <0.01 | 1.10 [-6.82, 9.02], 0.78 | | 1.00 |
| R.cMFG | 6779.30 [6504.07, 7054.54], <0.01 | -178.36 [-383.25, 26.54], 0.09 | -28.99 [-168.19, 110.21], 0.68 | 512.06 [301.81, 722.31], <0.01 | 12.99 [-3.95, 29.92], 0.13 | | 0.99 |
| R.CUN | 3078.86 [2915.51, 3242.20], <0.01 | 58.55 [-63.04, 180.15], 0.34 | -8.76 [-91.37, 73.85], 0.83 | 186.35 [61.57, 311.12], <0.01 | -2.45 [-12.50, 7.61], 0.63 | | 1.00 |
| R.ENT | 1827.85 [1717.18, 1938.53], <0.01 | 0.64 [-81.75, 83.03], 0.99 | -24.07 [-80.05, 31.90], 0.40 | 93.65 [9.10, 178.19], 0.03 | 2.53 [-4.28, 9.34], 0.46 | | 1.00 |
| R.FUS | 9071.77 [8798.84, 9344.70], <0.01 | -106.84 [-310.02, 96.34], 0.30 | -25.43 [-163.47, 112.60], 0.72 | 839.04 [630.55, 1047.53], <0.01 | -1.94 [-18.74, 14.85], 0.82 | | 1.00 |
| R.IPL | 14991.75 [14513.68, 15469.82], <0.01 | 124.67 [-231.22, 480.56], 0.49 | 71.30 [-170.48, 313.07], 0.56 | 1200.73 [835.54, 1565.92], <0.01 | 23.93 [-5.49, 53.35], 0.11 | | 1.00 |
| R.ITG | 11119.99 [10753.28, 11486.71], <0.01 | 30.96 [-242.03, 303.95], 0.82 | 49.00 [-136.47, 234.46], 0.60 | 984.26 [704.13, 1264.38], <0.01 | 18.03 [-4.54, 40.60], 0.12 | | 1.00 |
| R.iCC | 2456.95 [2364.70, 2549.19], <0.01 | 25.86 [-42.81, 94.53], 0.46 | 33.12 [-13.54, 79.77], 0.16 | 169.97 [99.51, 240.44], <0.01 | 2.66 [-3.01, 8.34], 0.35 | | 1.00 |
| R.LOG | 11510.32 [11131.19, 11889.45], <0.01 | 297.53 [15.29, 579.76], 0.04 | -84.12 [-275.86, 107.61], 0.39 | 569.21 [279.60, 858.82], <0.01 | -0.07 [-23.40, 23.26], 1.00 | | 0.90 |
| R.LOF | 7869.08 [7609.42, 8128.74], <0.01 | -86.78 [-280.08, 106.52], 0.37 | -48.58 [-179.90, 82.74], 0.46 | 629.31 [430.95, 827.66], <0.01 | 5.97 [-10.01, 21.95], 0.46 | | 1.00 |
| R.LING | 6505.50 [6231.68, 6779.31], <0.01 | -49.06 [-252.90, 154.78], 0.63 | -75.92 [-214.40, 62.56], 0.28 | 334.72 [125.56, 543.89], <0.01 | -12.46 [-29.31, 4.39], 0.15 | | 1.00 |
| R.MOF | 5827.34 [5690.05, 5964.64], <0.01 | -16.45 [-118.65, 85.76], 0.75 | -26.30 [-95.74, 43.13], 0.45 | 456.53 [351.65, 561.41], <0.01 | 5.94 [-2.51, 14.39], 0.17 | | 1.00 |
| R.MTG | 12562.50 [12190.62, 12934.37], <0.01 | -56.82 [-333.65, 220.02], 0.68 | -89.89 [-277.96, 98.18], 0.34 | 838.18 [554.11, 1122.25], <0.01 | -1.79 [-24.68, 21.09], 0.88 | | 1.00 |
| R.PARH | 2016.25 [1946.62, 2085.87], <0.01 | -28.14 [-79.97, 23.69], 0.28 | 10.52 [-24.70, 45.73], 0.55 | 88.22 [35.03, 141.41], <0.01 | -1.11 [-5.40, 3.17], 0.61 | | 1.00 |
| R.paraC | 3759.74 [3623.87, 3895.61], <0.01 | 77.57 [-23.58, 178.71], 0.13 | 20.86 [-47.86, 89.57], 0.55 | 220.27 [116.48, 324.06], <0.01 | -1.64 [-10.00, 6.72], 0.70 | | 1.00 |
| R.pOPER | 4215.30 [4056.11, 4374.50], <0.01 | -19.37 [-137.88, 99.14], 0.75 | -84.85 [-165.36, -4.34], 0.04 | 383.72 [262.11, 505.32], <0.01 | -1.25 [-11.05, 8.54], 0.80 | | 1.00 |
| R.pORB | 3144.71 [3006.05, 3283.37], <0.01 | -65.48 [-168.70, 37.74], 0.21 | -42.66 [-112.79, 27.46], 0.23 | 245.29 [139.37, 351.21], <0.01 | 1.85 [-6.68, 10.39], 0.67 | | 1.00 |
| R.pTRI | 4504.02 [4302.43, 4705.60], <0.01 | 37.80 [-112.26, 187.87], 0.62 | -46.06 [-148.01, 55.88], 0.37 | 433.34 [279.35, 587.32], <0.01 | -10.45 [-22.86, 1.95], 0.10 | | 1.00 |
| R.periCAL | 1918.06 [1793.62, 2042.50], <0.01 | 60.56 [-32.07, 153.20], 0.20 | 16.33 [-46.60, 79.27], 0.61 | 133.90 [38.84, 228.95], <0.01 | 0.63 [-7.03, 8.28], 0.87 | | 1.00 |
| R.postC | 8964.01 [8669.95, 9258.06], <0.01 | -48.64 [-267.54, 170.26], 0.66 | 112.89 [-35.82, 261.61], 0.13 | 813.11 [588.49, 1037.73], <0.01 | 3.45 [-14.65, 21.54], 0.71 | | 1.00 |
| R.PCC | 3380.58 [3269.29, 3491.87], <0.01 | -92.85 [-175.70, -10.01], 0.03 | -38.30 [-94.59, 17.98], 0.18 | 279.74 [194.73, 364.75], <0.01 | -0.14 [-6.99, 6.71], 0.97 | | 0.82 |
| R.preC | 12970.04 [12657.58, 13282.50], <0.01 | -112.33 [-344.94, 120.28], 0.34 | 77.04 [-80.98, 235.07], 0.34 | 606.89 [368.20, 845.57], <0.01 | 10.81 [-8.41, 30.04], 0.27 | | 1.00 |
| R.PCUN | 9770.59 [9500.97, 10040.20], <0.01 | 284.91 [84.20, 485.62], <0.01 | 83.63 [-52.73, 219.98], 0.23 | 851.92 [645.97, 1057.88], <0.01 | 18.71 [2.12, 35.30], 0.03 | | 0.33 |
| R.rACC | 2172.26 [2055.68, 2288.84], <0.01 | -29.13 [-115.91, 57.66], 0.51 | -5.59 [-64.55, 53.37], 0.85 | 223.45 [134.39, 312.50], <0.01 | 2.32 [-4.85, 9.50], 0.52 | | 1.00 |
| R.rMFG | 17463.96 [16990.08, 17937.84], <0.01 | -217.61 [-570.38, 135.16], 0.22 | -215.04 [-454.70, 24.62], 0.08 | 1666.17 [1304.18, 2028.16], <0.01 | -17.08 [-46.25, 12.08], 0.25 | | 1.00 |
| R.SFG | 23662.08 [23074.37, 24249.79], <0.01 | 287.74 [-149.77, 725.25], 0.19 | 48.30 [-248.93, 345.53], 0.75 | 1352.31 [903.37, 1801.25], <0.01 | 31.21 [-4.96, 67.38], 0.09 | | 1.00 |
| R.SPL | 12697.83 [12253.59, 13142.08], <0.01 | 112.44 [-218.26, 443.15], 0.50 | 93.42 [-131.25, 318.09], 0.41 | 1002.35 [663.00, 1341.71], <0.01 | 13.15 [-14.19, 40.49], 0.34 | | 1.00 |
| R.STG | 11957.50 [11596.43, 12318.56], <0.01 | -24.77 [-293.56, 244.02], 0.86 | -76.00 [-258.61, 106.60], 0.41 | 904.99 [629.18, 1180.81], <0.01 | -5.56 [-27.78, 16.66], 0.62 | | 1.00 |
| R.SMAR | 10577.16 [10226.73, 10927.60], <0.01 | -66.55 [-327.42, 194.32], 0.61 | 24.81 [-152.42, 202.03], 0.78 | 891.92 [624.23, 1159.61], <0.01 | -0.33 [-21.90, 21.24], 0.98 | | 1.00 |
| R.FP | 1349.27 [1290.09, 1408.44], <0.01 | 0.03 [-44.02, 44.09], 1.00 | -42.18 [-72.10, -12.25], <0.01 | 43.24 [-1.96, 88.45], 0.06 | 0.70 [-2.94, 4.35], 0.70 | | 1.00 |
| R.TP | 2350.62 [2237.18, 2464.05], <0.01 | 41.97 [-42.47, 126.42], 0.33 | 8.34 [-49.03, 65.71], 0.77 | 57.54 [-29.11, 144.20], 0.19 | 5.85 [-1.14, 12.83], 0.10 | | 1.00 |
| R.TT | 860.87 [822.12, 899.61], <0.01 | 4.79 [-24.05, 33.63], 0.74 | -3.46 [-23.05, 16.14], 0.73 | 74.84 [45.25, 104.44], <0.01 | -1.86 [-4.24, 0.52], 0.12 | | 1.00 |
| R.INS | 7192.34 [6984.50, 7400.18], <0.01 | -22.02 [-176.74, 132.70], 0.78 | 33.42 [-71.69, 138.53], 0.53 | 481.65 [322.88, 640.41], <0.01 | 5.18 [-7.61, 17.97], 0.42 | | 1.00 |
| L.THAL | 8008.97 [7807.44, 8210.49], <0.01 | -57.56 [-207.59, 92.46], 0.45 | 11.38 [-90.55, 113.30], 0.82 | 636.16 [482.22, 790.11], <0.01 | 10.43 [-1.97, 22.83], 0.10 | | 1.00 |
| R.THAL | 7461.07 [7273.76, 7648.39], <0.01 | -38.05 [-177.49, 101.40], 0.59 | -25.97 [-120.71, 68.76], 0.59 | 526.60 [383.51, 669.69], <0.01 | 7.46 [-4.07, 18.99], 0.20 | | 1.00 |
| L.PUT | 4113.79 [3952.87, 4274.70], <0.01 | 126.02 [6.23, 245.80], 0.04 | -7.99 [-89.36, 73.39], 0.85 | 253.52 [130.60, 376.44], <0.01 | -3.20 [-13.10, 6.71], 0.52 | | 0.90 |
| R.PUT | 4206.05 [4035.79, 4376.32], <0.01 | 150.44 [23.69, 277.19], 0.02 | 11.53 [-74.58, 97.64], 0.79 | 241.24 [111.18, 371.31], <0.01 | -3.82 [-14.30, 6.66], 0.47 | | 0.72 |
| L.PALL | 1837.11 [1760.94, 1913.29], <0.01 | -16.15 [-72.86, 40.56], 0.57 | 19.45 [-19.08, 57.98], 0.32 | 140.98 [82.79, 199.17], <0.01 | -0.32 [-5.01, 4.36], 0.89 | | 1.00 |
| R.PALL | 1730.68 [1661.14, 1800.22], <0.01 | 36.15 [-15.62, 87.91], 0.17 | -10.44 [-45.61, 24.73], 0.56 | 122.43 [69.31, 175.55], <0.01 | -1.05 [-5.33, 3.23], 0.63 | | 1.00 |
| L.CAUD | 3361.04 [3253.82, 3468.26], <0.01 | -11.87 [-91.68, 67.95], 0.77 | -45.58 [-99.81, 8.64], 0.10 | 286.60 [204.69, 368.50], <0.01 | -3.21 [-9.81, 3.38], 0.34 | | 1.00 |
| R.CAUD | 3368.95 [3265.43, 3472.47], <0.01 | 13.50 [-63.56, 90.57], 0.73 | -49.05 [-101.40, 3.31], 0.07 | 285.50 [206.42, 364.58], <0.01 | -2.55 [-8.92, 3.82], 0.43 | | 1.00 |
| L.HIPP | 4040.27 [3956.50, 4124.04], <0.01 | -37.85 [-100.21, 24.50], 0.23 | 7.63 [-34.73, 50.00], 0.72 | 240.40 [176.41, 304.39], <0.01 | 4.84 [-0.31, 10.00], 0.07 | | 1.00 |
| R.HIPP | 3949.81 [3863.52, 4036.09], <0.01 | 13.25 [-50.98, 77.49], 0.68 | 16.66 [-26.98, 60.29], 0.45 | 220.47 [154.56, 286.38], <0.01 | -0.41 [-5.72, 4.90], 0.88 | | 1.00 |
| L.AMYG | 1526.10 [1479.49, 1572.70], <0.01 | -19.35 [-54.05, 15.35], 0.27 | 16.93 [-6.64, 40.50], 0.16 | 86.46 [50.86, 122.06], <0.01 | 0.43 [-2.44, 3.30], 0.77 | | 1.00 |
| R.AMYG | 1631.98 [1588.34, 1675.62], <0.01 | -10.75 [-43.24, 21.73], 0.51 | -2.46 [-24.53, 19.61], 0.83 | 108.53 [75.19, 141.86], <0.01 | 1.81 [-0.88, 4.49], 0.18 | | 1.00 |
| L.AC | 494.44 [456.19, 532.69], <0.01 | -28.99 [-57.46, -0.51], 0.05 | 9.11 [-10.23, 28.46], 0.35 | 37.51 [8.29, 66.73], 0.01 | 2.42 [0.06, 4.77], 0.04 | | 0.94 |
| R.AC | 543.28 [519.02, 567.53], <0.01 | -22.06 [-40.11, -4.00], 0.02 | 5.05 [-7.21, 17.32], 0.42 | 35.24 [16.72, 53.77], <0.01 | 0.32 [-1.17, 1.81], 0.67 | | 0.66 |
| Shown are parameter estimates with 95% confidence intervals | | | | | | | |
| PvalCor = Multiple comparison corrected p-values | | | | | | | |

*Notes:* STiP-5.1=Semi-structured Interview for Personality Functioning DSM-5; GMV=grey matter volume; ICV=Estimated Intracranial Volume; IQ=Intelligence Quotient

**Table S11.** *Regression analysis results of STiP-5.1 total scores on selected ROIs GMV (N=93)*

|  | **(Intercept)** | **STiP-5.1 total** | **Age** | **ICV** | **IQ** | **PvalCor** | |
| --- | --- | --- | --- | --- | --- | --- | --- |
| L.AMYG | 1533.70 [1477.45, 1589.95], <0.01 | -22.81 [-63.10, 17.49], 0.26 | 16.54 [-7.10, 40.19], 0.17 | 84.50 [48.35, 120.66], <0.01 | 0.31 [-2.59, 3.22], 0.83 | | 0.91 |
| R.AMYG | 1640.47 [1587.89, 1693.05], <0.01 | -16.51 [-54.18, 21.15], 0.39 | -2.97 [-25.06, 19.13], 0.79 | 106.72 [72.92, 140.51], <0.01 | 1.68 [-1.03, 4.40], 0.22 | | 0.98 |
| L.HIPP | 4046.78 [3945.42, 4148.14], <0.01 | -37.09 [-109.70, 35.51], 0.31 | 7.46 [-35.14, 50.06], 0.73 | 237.99 [172.84, 303.13], <0.01 | 4.73 [-0.50, 9.96], 0.08 | | 0.95 |
| R.HIPP | 3961.64 [3857.38, 4065.90], <0.01 | 0.28 [-74.40, 74.97], 0.99 | 15.75 [-28.07, 59.57], 0.48 | 218.93 [151.92, 285.93], <0.01 | -0.56 [-5.95, 4.82], 0.84 | | 1.00 |
| Left_ACC | 4685.14 [4408.69, 4961.60], <0.01 | -94.35 [-292.39, 103.68], 0.35 | 71.93 [-44.26, 188.12], 0.22 | 388.71 [211.03, 566.38], <0.01 | 4.45 [-9.82, 18.72], 0.54 | | 0.97 |
| Right_ACC | 4238.82 [3975.90, 4501.75], <0.01 | -24.38 [-212.72, 163.96], 0.80 | -20.23 [-130.74, 90.27], 0.72 | 455.90 [286.92, 624.88], <0.01 | 3.37 [-10.20, 16.94], 0.62 | | 1.00 |
| Left_OFC | 16269.15 [15749.80, 16788.50], <0.01 | -4.82 [-376.84, 367.21], 0.98 | -3.44 [-221.71, 214.84], 0.98 | 1176.05 [842.27, 1509.83], <0.01 | 26.74 [-0.06, 53.54], 0.05 | | 1.00 |
| Right_OFC | 16846.13 [16311.84, 17380.42], <0.01 | -143.67 [-526.40, 239.06], 0.46 | -116.68 [-341.24, 107.87], 0.30 | 1324.44 [981.06, 1667.82], <0.01 | 13.59 [-13.99, 41.16], 0.33 | | 0.99 |
| Left_DLPFC | 49446.81 [48264.98, 50628.64], <0.01 | -323.49 [-1170.07, 523.08], 0.45 | -288.57 [-785.28, 208.13], 0.25 | 3704.13 [2944.59, 4463.68], <0.01 | 18.14 [-42.86, 79.13], 0.56 | | 0.99 |
| Right_DLPFC | 48141.89 [46988.07, 49295.71], <0.01 | -302.24 [-1128.76, 524.27], 0.47 | -211.24 [-696.17, 273.69], 0.39 | 3486.76 [2745.22, 4228.30], <0.01 | 23.80 [-35.75, 83.35], 0.43 | | 0.99 |
| Shown are parameter estimates with 95% confidence intervals | | | | | | | |
| PvalCor = Multiple comparison corrected p-values for group difference parameter *Notes:* STiP-5.1=Semi-structured Interview for Personality Functioning DSM-5; ROIs=regions of interest; GMV=grey matter volume; ICV=Estimated Intracranial Volume; IQ=Intelligence Quotient | | | | | | | |

**Table S12.** *Regression analysis results of STiP-5.1 Identity on selected ROIs GMV (N=93)*

|  | **(Intercept)** | **STiP-5.1 Identity** | **Age** | **ICV** | **IQ** | **PvalCor** | |
| --- | --- | --- | --- | --- | --- | --- | --- |
| L.AMYG | 1532.63 [1476.79, 1588.46], <0.01 | -14.57 [-41.14, 11.99], 0.28 | 16.50 [-7.18, 40.17], 0.17 | 84.30 [48.00, 120.60], <0.01 | 0.47 [-2.39, 3.33], 0.74 | | 0.92 |
| R.AMYG | 1635.96 [1583.69, 1688.22], <0.01 | -8.31 [-33.18, 16.56], 0.51 | -2.72 [-24.89, 19.44], 0.81 | 107.26 [73.28, 141.24], <0.01 | 1.83 [-0.85, 4.50], 0.18 | | 1.00 |
| L.HIPP | 4048.86 [3948.37, 4149.35], <0.01 | -26.00 [-73.81, 21.81], 0.28 | 7.10 [-35.51, 49.71], 0.74 | 236.95 [171.62, 302.28], <0.01 | 4.96 [-0.19, 10.11], 0.06 | | 0.92 |
| R.HIPP | 3967.47 [3864.04, 4070.90], <0.01 | -3.32 [-52.53, 45.89], 0.89 | 15.32 [-28.54, 59.18], 0.49 | 217.85 [150.61, 285.10], <0.01 | -0.61 [-5.91, 4.69], 0.82 | | 1.00 |
| Left_ACC | 4678.42 [4403.98, 4952.85], <0.01 | -58.92 [-189.49, 71.65], 0.37 | 71.91 [-44.46, 188.28], 0.22 | 388.29 [209.88, 566.71], <0.01 | 5.13 [-8.93, 19.18], 0.47 | | 0.97 |
| Right_ACC | 4168.35 [3907.64, 4429.06], <0.01 | 26.06 [-97.97, 150.10], 0.68 | -15.17 [-125.72, 95.38], 0.79 | 468.51 [299.02, 638.01], <0.01 | 4.08 [-9.28, 17.43], 0.55 | | 1.00 |
| Left_OFC | 16306.12 [15790.96, 16821.29], <0.01 | -25.42 [-270.52, 219.68], 0.84 | -6.19 [-224.64, 212.25], 0.96 | 1169.12 [834.21, 1504.04], <0.01 | 26.48 [0.10, 52.87], 0.05 | | 1.00 |
| Right_OFC | 16830.97 [16300.59, 17361.35], <0.01 | -86.76 [-339.10, 165.58], 0.50 | -116.35 [-341.25, 108.55], 0.31 | 1324.72 [979.91, 1669.53], <0.01 | 14.65 [-12.51, 41.81], 0.29 | | 1.00 |
| Left_DLPFC | 49283.87 [48108.63, 50459.10], <0.01 | -117.97 [-677.12, 441.18], 0.68 | -278.33 [-776.67, 220.01], 0.27 | 3728.60 [2964.55, 4492.65], <0.01 | 21.54 [-38.65, 81.73], 0.48 | | 1.00 |
| Right_DLPFC | 48224.24 [47081.48, 49367.00], <0.01 | -251.14 [-794.84, 292.55], 0.36 | -218.97 [-703.54, 265.60], 0.37 | 3466.19 [2723.26, 4209.12], <0.01 | 25.15 [-33.37, 83.68], 0.40 | | 0.97 |
| Shown are parameter estimates with 95% confidence intervals | | | | | | | |
| PvalCor = Multiple comparison corrected p-values *Notes:* STiP-5.1=Semi-structured Interview for Personality Functioning DSM-5; ROIs=regions of interest; GMV=grey matter volume; ICV=Estimated Intracranial Volume; IQ=Intelligence Quotient | | | | | | | |

**Table S13.** *Regression analysis results for Self-direction predicting selected ROIs GMV (N=93)*

|  | **(Intercept)** | **STiP-5.1 Self-direction** | **Age** | **ICV** | **IQ** | **PvalCor** | |
| --- | --- | --- | --- | --- | --- | --- | --- |
| L.AMYG | 1528.92 [1475.12, 1582.72], <0.01 | -18.63 [-56.33, 19.06], 0.33 | 17.19 [-6.40, 40.77], 0.15 | 84.44 [47.96, 120.93], <0.01 | 0.39 [-2.50, 3.29], 0.79 | | 0.95 |
| R.AMYG | 1646.44 [1596.46, 1696.41], <0.01 | -22.04 [-57.06, 12.98], 0.21 | -3.00 [-24.91, 18.91], 0.79 | 104.68 [70.78, 138.57], <0.01 | 1.62 [-1.07, 4.31], 0.23 | | 0.84 |
| L.HIPP | 4042.88 [3946.08, 4139.67], <0.01 | -33.81 [-101.64, 34.02], 0.32 | 8.30 [-34.14, 50.73], 0.70 | 237.07 [171.42, 302.72], <0.01 | 4.81 [-0.40, 10.02], 0.07 | | 0.95 |
| R.HIPP | 3962.71 [3863.17, 4062.25], <0.01 | -0.69 [-70.44, 69.06], 0.98 | 15.68 [-27.96, 59.33], 0.48 | 218.71 [151.20, 286.22], <0.01 | -0.58 [-5.93, 4.78], 0.83 | | 1.00 |
| Left_ACC | 4642.05 [4377.31, 4906.79], <0.01 | -55.94 [-241.46, 129.57], 0.55 | 75.84 [-40.23, 191.91], 0.20 | 393.39 [213.83, 572.95], <0.01 | 5.09 [-9.15, 19.33], 0.48 | | 1.00 |
| Right_ACC | 4255.03 [4004.20, 4505.87], <0.01 | -39.24 [-215.01, 136.52], 0.66 | -20.69 [-130.66, 89.29], 0.71 | 451.32 [281.20, 621.45], <0.01 | 3.17 [-10.32, 16.67], 0.64 | | 1.00 |
| Left_OFC | 16218.18 [15722.50, 16713.86], <0.01 | 41.36 [-305.98, 388.69], 0.81 | -0.63 [-217.95, 216.69], 1.00 | 1186.61 [850.42, 1522.80], <0.01 | 27.41 [0.75, 54.07], 0.04 | | 1.00 |
| Right_OFC | 16763.73 [16252.46, 17275.00], <0.01 | -69.97 [-428.23, 288.29], 0.70 | -109.83 [-333.99, 114.32], 0.33 | 1335.12 [988.36, 1681.88], <0.01 | 14.77 [-12.73, 42.27], 0.29 | | 1.00 |
| Left_DLPFC | 49354.74 [48225.11, 50484.37], <0.01 | -242.28 [-1033.85, 549.28], 0.54 | -278.15 [-773.41, 217.11], 0.27 | 3708.40 [2942.25, 4474.56], <0.01 | 19.58 [-41.18, 80.35], 0.52 | | 1.00 |
| Right_DLPFC | 48067.79 [46965.25, 49170.34], <0.01 | -237.17 [-1009.76, 535.41], 0.54 | -202.14 [-685.52, 281.24], 0.41 | 3488.22 [2740.44, 4236.01], <0.01 | 25.00 [-34.31, 84.30], 0.40 | | 1.00 |
| Shown are parameter estimates with 95% confidence intervals | | | | | | | |
| PvalCor = Multiple comparison corrected p-values *Notes:* STiP-5.1=Semi-structured Interview for Personality Functioning DSM-5; ROIs=regions of interest; GMV=grey matter volume; ICV=Estimated Intracranial Volume; IQ=Intelligence Quotient | | | | | | | |

**Table S14.** *Regression analysis results of STiP-5.1 Empathy on selected ROIs GMV (N=93)*

|  | **(Intercept)** | **STiP-5.1 Empathy** | **Age** | **ICV** | **IQ** | **PvalCor** | |
| --- | --- | --- | --- | --- | --- | --- | --- |
| L.AMYG | 1520.93 [1468.93, 1572.92], <0.01 | -16.55 [-68.11, 35.01], 0.53 | 17.40 [-6.32, 41.11], 0.15 | 87.30 [51.48, 123.13], <0.01 | 0.30 [-2.76, 3.36], 0.85 | | 1.00 |
| R.AMYG | 1630.90 [1582.39, 1679.41], <0.01 | -11.57 [-59.67, 36.53], 0.63 | -2.33 [-24.45, 19.80], 0.84 | 108.78 [75.36, 142.20], <0.01 | 1.68 [-1.17, 4.54], 0.24 | | 1.00 |
| L.HIPP | 4003.94 [3910.16, 4097.71], <0.01 | 2.17 [-90.82, 95.15], 0.96 | 10.41 [-32.37, 53.18], 0.63 | 245.16 [180.54, 309.77], <0.01 | 5.34 [-0.18, 10.87], 0.06 | | 1.00 |
| R.HIPP | 3972.19 [3876.34, 4068.05], <0.01 | -13.50 [-108.55, 81.55], 0.78 | 15.00 [-28.73, 58.73], 0.50 | 217.66 [151.61, 283.71], <0.01 | -0.86 [-6.51, 4.78], 0.76 | | 1.00 |
| Left_ACC | 4615.58 [4360.19, 4870.97], <0.01 | -46.44 [-299.68, 206.80], 0.72 | 76.65 [-39.85, 193.14], 0.19 | 402.27 [226.31, 578.23], <0.01 | 4.88 [-10.16, 19.93], 0.52 | | 1.00 |
| Right_ACC | 4306.47 [4066.01, 4546.93], <0.01 | -124.81 [-363.25, 113.63], 0.30 | -25.08 [-134.76, 84.61], 0.65 | 449.25 [283.57, 614.93], <0.01 | 1.03 [-13.13, 15.20], 0.89 | | 0.94 |
| Left_OFC | 16235.26 [15757.62, 16712.90], <0.01 | 37.61 [-436.02, 511.23], 0.87 | -1.05 [-218.93, 216.83], 0.99 | 1180.34 [851.25, 1509.44], <0.01 | 27.63 [-0.51, 55.76], 0.05 | | 1.00 |
| Right_OFC | 16769.04 [16276.59, 17261.49], <0.01 | -108.70 [-597.01, 379.61], 0.66 | -111.54 [-336.18, 113.09], 0.33 | 1341.67 [1002.37, 1680.97], <0.01 | 13.42 [-15.59, 42.43], 0.36 | | 1.00 |
| Left_DLPFC | 49641.21 [48561.80, 50720.62], <0.01 | -729.61 [-1799.95, 340.73], 0.18 | -303.05 [-795.44, 189.33], 0.22 | 3699.30 [2955.58, 4443.02], <0.01 | 7.25 [-56.33, 70.83], 0.82 | | 0.78 |
| Right_DLPFC | 48067.87 [47005.95, 49129.78], <0.01 | -344.82 [-1397.82, 708.17], 0.52 | -206.67 [-691.07, 277.73], 0.40 | 3512.55 [2780.88, 4244.22], <0.01 | 20.93 [-41.62, 83.48], 0.51 | | 1.00 |
| Shown are parameter estimates with 95% confidence intervals | | | | | | | |
| PvalCor = Multiple comparison corrected p-values *Notes:* STiP-5.1=Semi-structured Interview for Personality Functioning DSM-5; ROIs=regions of interest; GMV=grey matter volume; ICV=Estimated Intracranial Volume; IQ=Intelligence Quotient | | | | | | | |

**Table S15.** *Regression analysis results for Intimacy predicting selected ROIs GMV (N=93)*

|  | **(Intercept)** | **STiP-5.1 Intimacy** | **Age** | **ICV** | **IQ** | **PvalCor** | |
| --- | --- | --- | --- | --- | --- | --- | --- |
| L.AMYG | 1526.10 [1479.49, 1572.70], <0.01 | -19.35 [-54.05, 15.35], 0.27 | 16.93 [-6.64, 40.50], 0.16 | 86.46 [50.86, 122.06], <0.01 | 0.43 [-2.44, 3.30], 0.77 | | 0.92 |
| R.AMYG | 1631.98 [1588.34, 1675.62], <0.01 | -10.75 [-43.24, 21.73], 0.51 | -2.46 [-24.53, 19.61], 0.83 | 108.53 [75.19, 141.86], <0.01 | 1.81 [-0.88, 4.49], 0.18 | | 1.00 |
| L.HIPP | 4040.27 [3956.50, 4124.04], <0.01 | -37.85 [-100.21, 24.50], 0.23 | 7.63 [-34.73, 50.00], 0.72 | 240.40 [176.41, 304.39], <0.01 | 4.84 [-0.31, 10.00], 0.07 | | 0.87 |
| R.HIPP | 3949.81 [3863.52, 4036.09], <0.01 | 13.25 [-50.98, 77.49], 0.68 | 16.66 [-26.98, 60.29], 0.45 | 220.47 [154.56, 286.38], <0.01 | -0.41 [-5.72, 4.90], 0.88 | | 1.00 |
| Left_ACC | 4681.50 [4453.48, 4909.53], <0.01 | -110.40 [-280.14, 59.35], 0.20 | 71.39 [-43.93, 186.71], 0.22 | 393.15 [218.96, 567.33], <0.01 | 4.57 [-9.46, 18.60], 0.52 | | 0.83 |
| Right_ACC | 4235.90 [4018.14, 4453.66], <0.01 | -26.36 [-188.46, 135.75], 0.75 | -20.22 [-130.35, 89.91], 0.72 | 457.31 [290.96, 623.65], <0.01 | 3.42 [-9.98, 16.82], 0.61 | | 1.00 |
| Left_OFC | 16285.08 [15854.91, 16715.26], <0.01 | -23.22 [-343.46, 297.01], 0.89 | -4.70 [-222.26, 212.85], 0.97 | 1174.16 [845.55, 1502.76], <0.01 | 26.54 [0.06, 53.01], 0.05 | | 1.00 |
| Right_OFC | 16841.14 [16399.74, 17282.53], <0.01 | -168.70 [-497.29, 159.89], 0.31 | -117.54 [-340.77, 105.69], 0.30 | 1331.12 [993.95, 1668.30], <0.01 | 13.76 [-13.40, 40.92], 0.32 | | 0.95 |
| Left_DLPFC | 49287.47 [48307.23, 50267.72], <0.01 | -218.25 [-947.98, 511.48], 0.55 | -279.17 [-774.92, 216.58], 0.27 | 3738.65 [2989.86, 4487.45], <0.01 | 20.46 [-39.86, 80.79], 0.50 | | 1.00 |
| Right_DLPFC | 47905.34 [46947.17, 48863.52], <0.01 | -108.23 [-821.53, 605.07], 0.76 | -195.74 [-680.32, 288.85], 0.42 | 3530.54 [2798.60, 4262.48], <0.01 | 27.11 [-31.85, 86.08], 0.36 | | 1.00 |
| Shown are parameter estimates with 95% confidence intervals | | | | | | | |
| PvalCor = Multiple comparison corrected p-values *Notes:* STiP-5.1=Semi-structured Interview for Personality Functioning DSM-5; ROIs=regions of interest; GMV=grey matter volume; ICV=Estimated Intracranial Volume; IQ=Intelligence Quotient | | | | | | | |

**Table S16.** *Regression analysis results of BPD criteria on total GMV (N=93)*

| Predictors | Estimates, [95% CI] p value |  |
| --- | --- | --- |
| (Intercept) | 659699.15 [650525.56, 668872.75] <0.01 |  |
| BPD criteria | -1036.65 [-3150.88, 1077.58] 0.33 |  |
| Age | -4.52 [-4105.28, 4096.24] 1.00 |  |
| ICV | 43416.93 [37150.79, 49683.07] <0.01 |  |
| IQ | 174.48 [-334.17, 683.14] 0.50 |  |
| Num.Obs. | 93 |  |
| R2 | 0.719 |  |
| Shown are parameter estimates with 95% confidence intervals *Notes:* BPD=Borderline Personality Disorder; GMV=grey matter volume; ICV=Estimated Intracranial Volume; IQ=Intelligence Quotient | |  |

**Table S17.** *Regression analysis results of BPD criteria on individual brain region GMV (N=93)*

|  | **(Intercept)** | **BPD criteria** | **Age** | **ICV** | **IQ** | **PvalCor** |
| --- | --- | --- | --- | --- | --- | --- |
| L.BSTS | 2578.23 [2444.14, 2712.31], <0.01 | -28.97 [-59.88, 1.93], 0.07 | -11.07 [-71.01, 48.87], 0.71 | 248.73 [157.14, 340.32], <0.01 | -5.56 [-12.99, 1.88], 0.14 | 0.98 |
| L.cACC | 1872.72 [1719.47, 2025.97], <0.01 | -2.52 [-37.84, 32.80], 0.89 | 13.72 [-54.78, 82.22], 0.69 | 173.45 [68.77, 278.13], <0.01 | -0.21 [-8.71, 8.28], 0.96 | 1.00 |
| L.cMFG | 6914.73 [6580.73, 7248.73], <0.01 | -35.34 [-112.31, 41.64], 0.36 | -159.17 [-308.47, -9.86], 0.04 | 250.71 [22.57, 478.86], 0.03 | -1.76 [-20.28, 16.76], 0.85 | 1.00 |
| L.CUN | 2873.24 [2697.12, 3049.36], <0.01 | -10.02 [-50.61, 30.57], 0.63 | 10.82 [-67.91, 89.55], 0.79 | 229.54 [109.24, 349.84], <0.01 | 0.64 [-9.12, 10.41], 0.90 | 1.00 |
| L.ENT | 1987.33 [1840.33, 2134.34], <0.01 | -14.84 [-48.72, 19.04], 0.39 | 9.86 [-55.85, 75.58], 0.77 | 69.25 [-31.16, 169.67], 0.17 | 0.32 [-7.83, 8.47], 0.94 | 1.00 |
| L.FUS | 9545.77 [9241.05, 9850.48], <0.01 | -47.51 [-117.73, 22.72], 0.18 | 107.44 [-28.77, 243.65], 0.12 | 737.25 [529.11, 945.39], <0.01 | 10.64 [-6.26, 27.53], 0.21 | 1.00 |
| L.IPL | 12384.47 [11901.65, 12867.29], <0.01 | -0.99 [-112.27, 110.28], 0.99 | 9.84 [-205.99, 225.67], 0.93 | 1007.31 [677.51, 1337.11], <0.01 | 2.68 [-24.10, 29.45], 0.84 | 1.00 |
| L.ITG | 11248.92 [10732.37, 11765.48], <0.01 | -4.18 [-123.23, 114.87], 0.94 | -35.41 [-266.32, 195.50], 0.76 | 839.42 [486.58, 1192.26], <0.01 | 4.49 [-24.15, 33.14], 0.76 | 1.00 |
| L.iCC | 2612.82 [2528.50, 2697.14], <0.01 | -7.37 [-26.80, 12.07], 0.45 | -19.07 [-56.76, 18.63], 0.32 | 175.89 [118.29, 233.49], <0.01 | 2.70 [-1.98, 7.37], 0.25 | 1.00 |
| L.LOG | 11425.08 [11020.86, 11829.30], <0.01 | 45.35 [-47.81, 138.51], 0.34 | 17.82 [-162.88, 198.51], 0.85 | 732.85 [456.74, 1008.96], <0.01 | -3.06 [-25.48, 19.35], 0.79 | 1.00 |
| L.LOF | 7961.50 [7680.19, 8242.81], <0.01 | -15.18 [-80.01, 49.66], 0.64 | 29.28 [-96.47, 155.04], 0.64 | 492.77 [300.61, 684.92], <0.01 | 16.05 [0.45, 31.65], 0.04 | 1.00 |
| L.LING | 6070.98 [5794.35, 6347.61], <0.01 | -45.19 [-108.95, 18.56], 0.16 | -61.61 [-185.26, 62.05], 0.32 | 335.15 [146.19, 524.11], <0.01 | 1.03 [-14.30, 16.37], 0.89 | 1.00 |
| L.MOF | 5680.28 [5470.66, 5889.89], <0.01 | 3.28 [-45.03, 51.59], 0.89 | -44.70 [-138.41, 49.00], 0.35 | 450.93 [307.75, 594.11], <0.01 | 5.48 [-6.14, 17.10], 0.35 | 1.00 |
| L.MTG | 11432.65 [10980.31, 11884.98], <0.01 | -19.44 [-123.69, 84.81], 0.71 | -106.44 [-308.64, 95.76], 0.30 | 897.82 [588.85, 1206.80], <0.01 | -15.32 [-40.40, 9.76], 0.23 | 1.00 |
| L.PARH | 2126.25 [2039.50, 2213.00], <0.01 | -5.08 [-25.08, 14.91], 0.61 | -9.77 [-48.55, 29.01], 0.62 | 53.51 [-5.75, 112.76], 0.08 | 5.97 [1.16, 10.78], 0.02 | 1.00 |
| L.paraC | 3511.38 [3380.06, 3642.69], <0.01 | -9.68 [-39.94, 20.59], 0.53 | 7.36 [-51.34, 66.06], 0.80 | 206.86 [117.16, 296.55], <0.01 | 2.99 [-4.29, 10.27], 0.42 | 1.00 |
| L.pOPER | 5039.59 [4829.59, 5249.59], <0.01 | 5.59 [-42.81, 53.99], 0.82 | -119.07 [-212.94, -25.20], 0.01 | 382.67 [239.23, 526.11], <0.01 | 1.83 [-9.81, 13.47], 0.76 | 1.00 |
| L.pORB | 2669.48 [2538.13, 2800.83], <0.01 | -2.46 [-32.73, 27.81], 0.87 | 14.34 [-44.38, 73.05], 0.63 | 225.77 [136.05, 315.49], <0.01 | 4.49 [-2.79, 11.78], 0.22 | 1.00 |
| L.pTRI | 4085.97 [3892.23, 4279.71], <0.01 | -6.57 [-51.22, 38.08], 0.77 | -1.58 [-88.19, 85.02], 0.97 | 390.33 [257.99, 522.66], <0.01 | 5.18 [-5.56, 15.93], 0.34 | 1.00 |
| L.periCAL | 1803.17 [1673.45, 1932.88], <0.01 | -0.38 [-30.27, 29.52], 0.98 | 6.51 [-51.48, 64.49], 0.82 | 128.04 [39.44, 216.65], <0.01 | 0.68 [-6.51, 7.87], 0.85 | 1.00 |
| L.postC | 9323.12 [8974.63, 9671.62], <0.01 | -15.93 [-96.25, 64.39], 0.69 | 107.07 [-48.71, 262.86], 0.18 | 741.00 [502.96, 979.05], <0.01 | 1.05 [-18.27, 20.37], 0.91 | 1.00 |
| L.PCC | 3241.29 [3105.54, 3377.04], <0.01 | -17.39 [-48.67, 13.90], 0.27 | 33.75 [-26.93, 94.43], 0.27 | 210.31 [117.59, 303.04], <0.01 | 5.35 [-2.17, 12.88], 0.16 | 1.00 |
| L.preC | 13296.09 [12922.96, 13669.22], <0.01 | -26.81 [-112.80, 59.19], 0.54 | -64.94 [-231.74, 101.85], 0.44 | 775.18 [520.31, 1030.05], <0.01 | 1.20 [-19.49, 21.89], 0.91 | 1.00 |
| L.PCUN | 9686.60 [9388.03, 9985.17], <0.01 | -11.01 [-79.83, 57.80], 0.75 | 99.67 [-33.79, 233.14], 0.14 | 769.88 [565.94, 973.82], <0.01 | 8.67 [-7.88, 25.23], 0.30 | 1.00 |
| L.rACC | 2823.05 [2673.30, 2972.80], <0.01 | -32.41 [-66.92, 2.10], 0.07 | 70.27 [3.32, 137.21], 0.04 | 214.78 [112.49, 317.07], <0.01 | 4.18 [-4.12, 12.48], 0.32 | 0.98 |
| L.rMFG | 17411.90 [16906.24, 17917.56], <0.01 | -24.79 [-141.33, 91.75], 0.67 | -94.80 [-320.84, 131.23], 0.41 | 1632.22 [1286.82, 1977.61], <0.01 | -2.09 [-30.13, 25.95], 0.88 | 1.00 |
| L.SFG | 25064.23 [24400.35, 25728.11], <0.01 | -31.69 [-184.69, 121.32], 0.68 | 2.85 [-293.91, 299.62], 0.98 | 1834.15 [1380.68, 2287.62], <0.01 | 21.86 [-14.95, 58.67], 0.24 | 1.00 |
| L.SPL | 13088.19 [12584.68, 13591.70], <0.01 | -6.06 [-122.10, 109.98], 0.92 | 131.45 [-93.63, 356.53], 0.25 | 927.76 [583.84, 1271.69], <0.01 | 0.42 [-27.50, 28.34], 0.98 | 1.00 |
| L.STG | 12705.87 [12310.31, 13101.43], <0.01 | -63.93 [-155.09, 27.24], 0.17 | 39.85 [-136.97, 216.68], 0.66 | 862.17 [591.98, 1132.36], <0.01 | -6.35 [-28.28, 15.58], 0.57 | 1.00 |
| L.SMAR | 11940.19 [11393.03, 12487.36], <0.01 | -32.89 [-158.99, 93.22], 0.61 | -10.92 [-255.51, 233.67], 0.93 | 1172.56 [798.82, 1546.31], <0.01 | 6.90 [-23.43, 37.24], 0.65 | 1.00 |
| L.FP | 1112.50 [1061.07, 1163.93], <0.01 | 4.78 [-7.07, 16.63], 0.43 | -21.34 [-44.33, 1.65], 0.07 | 24.06 [-11.07, 59.19], 0.18 | 1.03 [-1.82, 3.88], 0.47 | 1.00 |
| L.TP | 2421.74 [2253.31, 2590.18], <0.01 | -8.11 [-46.93, 30.71], 0.68 | 40.53 [-34.77, 115.82], 0.29 | 40.12 [-74.93, 155.17], 0.49 | 2.49 [-6.85, 11.83], 0.60 | 1.00 |
| L.TT | 1100.10 [1043.03, 1157.16], <0.01 | -9.56 [-22.72, 3.59], 0.15 | 11.39 [-14.12, 36.90], 0.38 | 58.41 [19.43, 97.39], <0.01 | -1.11 [-4.28, 2.05], 0.49 | 1.00 |
| L.INS | 7282.47 [7084.42, 7480.52], <0.01 | -28.30 [-73.95, 17.34], 0.22 | 38.32 [-50.21, 126.85], 0.39 | 381.99 [246.71, 517.27], <0.01 | 5.03 [-5.95, 16.01], 0.37 | 1.00 |
| R.BSTS | 2389.31 [2280.91, 2497.71], <0.01 | -11.01 [-35.99, 13.97], 0.38 | -12.49 [-60.95, 35.97], 0.61 | 171.86 [97.82, 245.91], <0.01 | -1.00 [-7.01, 5.01], 0.74 | 1.00 |
| R.cACC | 2062.65 [1917.48, 2207.83], <0.01 | 1.07 [-32.39, 34.52], 0.95 | -14.98 [-79.87, 49.92], 0.65 | 234.08 [134.92, 333.25], <0.01 | 1.13 [-6.92, 9.18], 0.78 | 1.00 |
| R.cMFG | 6912.16 [6607.35, 7216.96], <0.01 | -89.67 [-159.92, -19.42], 0.01 | -4.04 [-140.30, 132.21], 0.95 | 486.80 [278.60, 695.00], <0.01 | 10.18 [-6.72, 27.08], 0.23 | 0.55 |
| R.CUN | 3125.18 [2939.95, 3310.41], <0.01 | 2.22 [-40.47, 44.91], 0.92 | -13.18 [-95.98, 69.62], 0.75 | 180.45 [53.93, 306.97], <0.01 | -3.02 [-13.29, 7.25], 0.56 | 1.00 |
| R.ENT | 1905.60 [1782.60, 2028.61], <0.01 | -23.35 [-51.70, 5.00], 0.11 | -20.88 [-75.87, 34.11], 0.45 | 81.39 [-2.63, 165.41], 0.06 | 1.24 [-5.58, 8.06], 0.72 | 1.00 |
| R.FUS | 9212.53 [8909.90, 9515.16], <0.01 | -72.23 [-141.98, -2.48], 0.04 | -7.92 [-143.20, 127.36], 0.91 | 814.25 [607.53, 1020.96], <0.01 | -4.65 [-21.43, 12.13], 0.58 | 0.92 |
| R.IPL | 14949.76 [14410.68, 15488.84], <0.01 | 47.28 [-76.96, 171.52], 0.45 | 55.99 [-184.99, 296.97], 0.65 | 1210.37 [842.14, 1578.59], <0.01 | 25.04 [-4.85, 54.93], 0.10 | 1.00 |
| R.ITG | 11064.74 [10651.56, 11477.93], <0.01 | 25.31 [-69.92, 120.53], 0.60 | 43.31 [-141.39, 228.02], 0.64 | 993.72 [711.49, 1275.95], <0.01 | 19.05 [-3.86, 41.96], 0.10 | 1.00 |
| R.iCC | 2457.98 [2353.77, 2562.19], <0.01 | 6.86 [-17.16, 30.88], 0.57 | 30.35 [-16.23, 76.93], 0.20 | 170.43 [99.25, 241.61], <0.01 | 2.73 [-3.05, 8.51], 0.35 | 1.00 |
| R.LOG | 11452.11 [11023.62, 11880.60], <0.01 | 100.13 [1.37, 198.88], 0.05 | -118.89 [-310.43, 72.65], 0.22 | 585.57 [292.88, 878.25], <0.01 | 1.89 [-21.87, 25.65], 0.87 | 0.94 |
| R.LOF | 7904.88 [7612.37, 8197.40], <0.01 | -34.90 [-102.32, 32.52], 0.31 | -37.65 [-168.41, 93.11], 0.57 | 621.56 [421.76, 821.37], <0.01 | 5.08 [-11.14, 21.30], 0.54 | 1.00 |
| R.LING | 6566.85 [6258.95, 6874.75], <0.01 | -32.17 [-103.13, 38.79], 0.37 | -68.02 [-205.66, 69.62], 0.33 | 323.86 [113.54, 534.17], <0.01 | -13.64 [-30.72, 3.43], 0.12 | 1.00 |
| R.MOF | 5870.21 [5716.06, 6024.35], <0.01 | -17.53 [-53.06, 17.99], 0.33 | -22.72 [-91.62, 46.19], 0.51 | 449.37 [344.08, 554.66], <0.01 | 5.17 [-3.38, 13.71], 0.23 | 1.00 |
| R.MTG | 12689.86 [12272.90, 13106.83], <0.01 | -54.30 [-150.40, 41.80], 0.26 | -78.38 [-264.77, 108.01], 0.41 | 816.71 [531.90, 1101.53], <0.01 | -4.11 [-27.23, 19.01], 0.72 | 1.00 |
| R.PARH | 2026.15 [1947.70, 2104.59], <0.01 | -10.80 [-28.88, 7.28], 0.24 | 13.99 [-21.08, 49.05], 0.43 | 85.98 [32.40, 139.56], <0.01 | -1.37 [-5.72, 2.98], 0.53 | 1.00 |
| R.paraC | 3810.23 [3655.03, 3965.42], <0.01 | 6.23 [-29.54, 42.00], 0.73 | 14.55 [-54.83, 83.92], 0.68 | 214.17 [108.16, 320.18], <0.01 | -2.22 [-10.83, 6.38], 0.61 | 1.00 |
| R.pOPER | 4232.00 [4052.55, 4411.45], <0.01 | -10.42 [-51.78, 30.93], 0.62 | -82.04 [-162.26, -1.83], 0.05 | 380.62 [258.04, 503.19], <0.01 | -1.60 [-11.55, 8.35], 0.75 | 1.00 |
| R.pORB | 3116.59 [2959.00, 3274.18], <0.01 | -9.65 [-45.97, 26.67], 0.60 | -36.73 [-107.17, 33.72], 0.30 | 248.15 [140.50, 355.79], <0.01 | 2.10 [-6.63, 10.84], 0.63 | 1.00 |
| R.pTRI | 4516.22 [4288.56, 4743.88], <0.01 | 6.79 [-45.68, 59.26], 0.80 | -49.66 [-151.43, 52.11], 0.33 | 432.32 [276.82, 587.83], <0.01 | -10.53 [-23.15, 2.10], 0.10 | 1.00 |
| R.periCAL | 1949.55 [1807.98, 2091.12], <0.01 | 7.27 [-25.36, 39.89], 0.66 | 11.07 [-52.21, 74.36], 0.73 | 130.39 [33.69, 227.09], <0.01 | 0.30 [-7.55, 8.15], 0.94 | 1.00 |
| R.postC | 9085.75 [8756.88, 9414.62], <0.01 | -50.33 [-126.13, 25.46], 0.19 | 123.28 [-23.73, 270.30], 0.10 | 792.73 [568.09, 1017.37], <0.01 | 1.26 [-16.98, 19.49], 0.89 | 1.00 |
| R.PCC | 3402.36 [3276.78, 3527.93], <0.01 | -32.34 [-61.28, -3.40], 0.03 | -27.30 [-83.44, 28.83], 0.34 | 274.07 [188.29, 359.84], <0.01 | -0.81 [-7.77, 6.15], 0.82 | 0.83 |
| R.preC | 12887.13 [12532.82, 13241.45], <0.01 | -6.06 [-87.72, 75.60], 0.88 | 85.77 [-72.62, 244.15], 0.28 | 617.26 [375.24, 859.28], <0.01 | 11.82 [-7.82, 31.47], 0.23 | 1.00 |
| R.PCUN | 9953.12 [9636.24, 10269.99], <0.01 | 23.77 [-49.26, 96.80], 0.52 | 60.33 [-81.32, 201.98], 0.40 | 829.99 [613.54, 1046.44], <0.01 | 16.62 [-0.95, 34.19], 0.06 | 1.00 |
| R.rACC | 2196.47 [2065.37, 2327.56], <0.01 | -15.40 [-45.62, 14.81], 0.31 | -1.41 [-60.01, 57.20], 0.96 | 218.92 [129.38, 308.47], <0.01 | 1.82 [-5.45, 9.09], 0.62 | 1.00 |
| R.rMFG | 17421.95 [16884.56, 17959.34], <0.01 | -47.64 [-171.49, 76.21], 0.45 | -193.16 [-433.39, 47.06], 0.11 | 1667.55 [1300.48, 2034.62], <0.01 | -17.11 [-46.91, 12.69], 0.26 | 1.00 |
| R.SFG | 23830.42 [23161.49, 24499.34], <0.01 | 28.85 [-125.31, 183.02], 0.71 | 24.10 [-274.92, 323.12], 0.87 | 1332.69 [875.77, 1789.60], <0.01 | 29.36 [-7.73, 66.45], 0.12 | 1.00 |
| R.SPL | 12906.85 [12405.23, 13408.48], <0.01 | -32.07 [-147.68, 83.54], 0.58 | 89.97 [-134.26, 314.21], 0.43 | 972.08 [629.44, 1314.72], <0.01 | 10.04 [-17.77, 37.86], 0.47 | 1.00 |
| R.STG | 12109.89 [11705.39, 12514.40], <0.01 | -52.99 [-146.21, 40.24], 0.26 | -66.92 [-247.74, 113.90], 0.46 | 880.35 [604.04, 1156.65], <0.01 | -8.18 [-30.61, 14.25], 0.47 | 1.00 |
| R.SMAR | 10710.12 [10317.88, 11102.36], <0.01 | -58.69 [-149.09, 31.71], 0.20 | 37.61 [-137.72, 212.95], 0.67 | 869.34 [601.41, 1137.26], <0.01 | -2.77 [-24.52, 18.98], 0.80 | 1.00 |
| R.FP | 1347.06 [1280.30, 1413.82], <0.01 | 0.68 [-14.71, 16.06], 0.93 | -42.27 [-72.11, -12.43], <0.01 | 43.59 [-2.01, 89.19], 0.06 | 0.74 [-2.96, 4.44], 0.69 | 1.00 |
| R.TP | 2380.02 [2251.36, 2508.68], <0.01 | 2.74 [-26.91, 32.40], 0.85 | 5.01 [-52.50, 62.53], 0.86 | 53.92 [-33.97, 141.80], 0.23 | 5.49 [-1.64, 12.63], 0.13 | 1.00 |
| R.TT | 880.05 [836.51, 923.60], <0.01 | -4.48 [-14.51, 5.56], 0.38 | -3.17 [-22.64, 16.29], 0.75 | 71.93 [42.19, 101.67], <0.01 | -2.16 [-4.58, 0.25], 0.08 | 1.00 |
| R.INS | 7304.02 [7072.32, 7535.73], <0.01 | -39.90 [-93.30, 13.50], 0.14 | 40.50 [-63.08, 144.07], 0.44 | 463.49 [305.22, 621.76], <0.01 | 3.24 [-9.60, 16.09], 0.62 | 1.00 |
| L.THAL | 8066.51 [7840.47, 8292.55], <0.01 | -33.38 [-85.47, 18.72], 0.21 | 20.04 [-81.00, 121.09], 0.69 | 625.70 [471.30, 780.10], <0.01 | 9.28 [-3.25, 21.82], 0.14 | 1.00 |
| R.THAL | 7545.44 [7336.38, 7754.51], <0.01 | -36.08 [-84.27, 12.10], 0.14 | -18.30 [-111.76, 75.15], 0.70 | 512.37 [369.56, 655.17], <0.01 | 5.93 [-5.67, 17.52], 0.31 | 1.00 |
| L.PUT | 4180.07 [3994.59, 4365.56], <0.01 | 14.89 [-27.86, 57.63], 0.49 | -18.90 [-101.81, 64.02], 0.65 | 246.10 [119.40, 372.80], <0.01 | -3.88 [-14.17, 6.40], 0.46 | 1.00 |
| R.PUT | 4277.31 [4080.11, 4474.51], <0.01 | 20.16 [-25.29, 65.61], 0.38 | -1.83 [-89.98, 86.33], 0.97 | 233.63 [98.92, 368.33], <0.01 | -4.51 [-15.44, 6.43], 0.42 | 1.00 |
| L.PALL | 1870.76 [1785.73, 1955.80], <0.01 | -14.66 [-34.26, 4.94], 0.14 | 22.62 [-15.40, 60.63], 0.24 | 135.28 [77.19, 193.37], <0.01 | -0.94 [-5.65, 3.78], 0.69 | 1.00 |
| R.PALL | 1748.42 [1669.23, 1827.61], <0.01 | 4.66 [-13.59, 22.91], 0.61 | -13.62 [-49.02, 21.78], 0.45 | 120.50 [66.41, 174.59], <0.01 | -1.23 [-5.62, 3.16], 0.58 | 1.00 |
| L.CAUD | 3410.58 [3290.73, 3530.43], <0.01 | -18.28 [-45.91, 9.34], 0.19 | -42.22 [-95.79, 11.36], 0.12 | 278.49 [196.63, 360.36], <0.01 | -4.08 [-10.73, 2.57], 0.23 | 1.00 |
| R.CAUD | 3385.33 [3268.46, 3502.20], <0.01 | -1.21 [-28.15, 25.72], 0.93 | -49.83 [-102.07, 2.41], 0.06 | 283.24 [203.42, 363.07], <0.01 | -2.78 [-9.26, 3.70], 0.40 | 1.00 |
| L.HIPP | 4062.67 [3968.72, 4156.62], <0.01 | -17.28 [-38.93, 4.38], 0.12 | 12.69 [-29.31, 54.68], 0.55 | 235.95 [171.78, 300.13], <0.01 | 4.34 [-0.86, 9.55], 0.10 | 1.00 |
| R.HIPP | 3972.36 [3874.96, 4069.76], <0.01 | -3.15 [-25.60, 19.30], 0.78 | 16.16 [-27.38, 59.70], 0.46 | 217.23 [150.70, 283.76], <0.01 | -0.74 [-6.14, 4.66], 0.79 | 1.00 |
| L.AMYG | 1539.77 [1487.54, 1591.99], <0.01 | -9.50 [-21.54, 2.53], 0.12 | 19.60 [-3.74, 42.95], 0.10 | 83.84 [48.16, 119.51], <0.01 | 0.14 [-2.76, 3.03], 0.93 | 1.00 |
| R.AMYG | 1660.63 [1612.46, 1708.81], <0.01 | -11.65 [-22.76, -0.55], 0.04 | -0.09 [-21.62, 21.45], 0.99 | 103.74 [70.84, 136.65], <0.01 | 1.29 [-1.38, 3.96], 0.34 | 0.91 |
| L.AC | 480.93 [436.94, 524.93], <0.01 | -3.95 [-14.09, 6.19], 0.44 | 11.69 [-7.97, 31.36], 0.24 | 38.94 [8.89, 69.00], 0.01 | 2.55 [0.11, 4.98], 0.04 | 1.00 |
| R.AC | 536.80 [508.79, 564.80], <0.01 | -4.16 [-10.61, 2.30], 0.20 | 7.18 [-5.34, 19.70], 0.26 | 35.73 [16.60, 54.86], <0.01 | 0.35 [-1.20, 1.91], 0.65 | 1.00 |
| Shown are parameter estimates with 95% confidence intervals | | | | | | |
| PvalCor = Multiple comparison corrected p-values for the bpd regression parameter | | | | | | |

*Notes:* BPD=Borderline Personality Disorder; GMV=grey matter volume; ICV=Estimated Intracranial Volume; IQ=Intelligence Quotient

**Table S18.** *Regression analysis results of BPD criteria on selected ROIs GMV (N=93)*

|  | **(Intercept)** | **BPD criteria** | **Age** | **ICV** | **IQ** | **PvalCor** | |
| --- | --- | --- | --- | --- | --- | --- | --- |
| L.AMYG | 1539.77 [1487.54, 1591.99], <0.01 | -9.50 [-21.54, 2.53], 0.12 | 19.60 [-3.74, 42.95], 0.10 | 83.84 [48.16, 119.51], <0.01 | 0.14 [-2.76, 3.03], 0.93 | | 0.64 |
| R.AMYG | 1660.63 [1612.46, 1708.81], <0.01 | -11.65 [-22.76, -0.55], 0.04 | -0.09 [-21.62, 21.45], 0.99 | 103.74 [70.84, 136.65], <0.01 | 1.29 [-1.38, 3.96], 0.34 | | 0.28 |
| L.HIPP | 4062.67 [3968.72, 4156.62], <0.01 | -17.28 [-38.93, 4.38], 0.12 | 12.69 [-29.31, 54.68], 0.55 | 235.95 [171.78, 300.13], <0.01 | 4.34 [-0.86, 9.55], 0.10 | | 0.63 |
| R.HIPP | 3972.36 [3874.96, 4069.76], <0.01 | -3.15 [-25.60, 19.30], 0.78 | 16.16 [-27.38, 59.70], 0.46 | 217.23 [150.70, 283.76], <0.01 | -0.74 [-6.14, 4.66], 0.79 | | 1.00 |
| Left_ACC | 4695.77 [4438.08, 4953.46], <0.01 | -34.93 [-94.32, 24.46], 0.25 | 83.98 [-31.21, 199.18], 0.15 | 388.23 [212.21, 564.25], <0.01 | 3.97 [-10.32, 18.25], 0.58 | | 0.90 |
| Right_ACC | 4259.12 [4013.66, 4504.58], <0.01 | -14.34 [-70.91, 42.23], 0.62 | -16.38 [-126.11, 93.34], 0.77 | 453.01 [285.34, 620.67], <0.01 | 2.95 [-10.66, 16.56], 0.67 | | 1.00 |
| Left_OFC | 16311.25 [15826.06, 16796.45], <0.01 | -14.36 [-126.18, 97.46], 0.80 | -1.08 [-217.97, 215.81], 0.99 | 1169.47 [838.05, 1500.89], <0.01 | 26.02 [-0.88, 52.93], 0.06 | | 1.00 |
| Right_OFC | 16891.68 [16394.03, 17389.33], <0.01 | -62.08 [-176.78, 52.61], 0.29 | -97.10 [-319.56, 125.36], 0.39 | 1319.08 [979.15, 1659.01], <0.01 | 12.36 [-15.24, 39.95], 0.38 | | 0.93 |
| Left_DLPFC | 49390.86 [48285.96, 50495.76], <0.01 | -91.81 [-346.46, 162.83], 0.48 | -251.12 [-745.03, 242.79], 0.32 | 3717.08 [2962.36, 4471.79], <0.01 | 18.01 [-43.25, 79.27], 0.56 | | 0.99 |
| Right_DLPFC | 48164.53 [47087.57, 49241.48], <0.01 | -108.45 [-356.66, 139.75], 0.39 | -173.11 [-654.53, 308.31], 0.48 | 3487.03 [2751.40, 4222.66], <0.01 | 22.43 [-37.28, 82.15], 0.46 | | 0.98 |
| Shown are parameter estimates with 95% confidence intervals | | | | | | | |
| PvalCor = Multiple comparison corrected p-values for group difference parameter *Notes:* BPD=Borderline Personality Disorder; GMV=grey matter volume; ICV=Estimated Intracranial Volume; IQ=Intelligence Quotient | | | | | | | |

**References for Supplementary Materials**

1. Beck AT, Steer RA, Brown G (1996): Beck depression inventory–II. *Psychol Assess*.

2. Osman A, Kopper BA, Barrios F, Gutierrez PM, Bagge CL (2004): Reliability and validity of the Beck depression inventory--II with adolescent psychiatric inpatients. *Psychol Assess* 16: 120–132.

3. Kühner C, Bürger C, Keller F, Hautzinger M (2007): Reliabilität und Validität des revidierten Beck-Depressionsinventars (BDI-II). *Nervenarzt* 78: 651–656.

4. Bernstein DP, Fink L, Handelsman L, Foote J, Lovejoy M, Wenzel K, *et al.* (1994): Initial reliability and validity of a new retrospective measure of child abuse and neglect. *Am J Psychiatry* 151: 1132–1136.

5. Wingenfeld K, Spitzer C, Mensebach C, Grabe HJ, Hill A, Gast U, *et al.* (2010): [The German version of the Childhood Trauma Questionnaire (CTQ): preliminary psychometric properties]. *Psychother Psychosom Med Psychol* 60: 442–450.

6. Oldfield RC (1971): The assessment and analysis of handedness: The Edinburgh inventory. *Neuropsychologia* 9: 97–113.

7. Hutsebaut J, Kamphuis JH, Feenstra DJ, Weekers LC, De Saeger H (2017): Assessing DSM–5-oriented level of personality functioning: Development and psychometric evaluation of the Semi-Structured Interview for Personality Functioning DSM–5 (STiP-5.1). *Personal Disord Theory Res Treat* 8: 94–101.

8. Hutsebaut J, Kamphuis H, Feenstra DJ, Weekers LC, De Saeger H (2017): Assessing DSM–5-oriented level of personality functioning: Development and psychometric evaluation of the Semi-Structured Interview for Personality Functioning DSM–5 (STiP-5.1). *Personal Disord Theory Res Treat* 8: 94–101.

9. Weekers LC, Verhoeff SCE, Kamphuis JH, Hutsebaut J (2021): Assessing Criterion A in adolescents using the Semistructured Interview for Personality Functioning DSM–5. *Personal Disord Theory Res Treat* 12: 312–319.

10. Fydrich T, Renneberg B, Schmitz B, Wittchen H-U (1997): SKID II. Strukturiertes Klinisches Interview für DSM-IV, Achse II: Persönlichkeitsstörungen. Interviewheft. Eine deutschspeachige, erw. Bearb. d. amerikanischen Originalversion d. SKID-II von: M.B. First, R.L. Spitzer, M. Gibbon, J.B.W. Williams, L. Benjamin, (Version 3/96). Retrieved December 23, 2020, from https://pure.mpg.de/pubman/faces/ViewItemOverviewPage.jsp?itemId=item_1646353

11. Chanen AM, Jovev M, Djaja D, McDougall E, Yuen HP, Rawlings D, Jackson HJ (2008): Screening for Borderline Personality Disorder in Outpatient Youth. *J Personal Disord* 22: 353–364.

12. Sheehan DV, Sheehan KH, Shytle RD, Janavs J, Bannon Y, Rogers JE, *et al.* (2010): Reliability and validity of the Mini International Neuropsychiatric Interview for Children and Adolescents (MINI-KID). *J Clin Psychiatry* 71: 313–326.

13. First MB, Spitzer RL, Gibbon M, Williams JBW (2002): *Structured Clinical Interview for DSM-IV-TR Axis I Disorders, Research Version, Non-Patient Edition. (SCID-I/NP)*. New York: Biometrics Research, New York State Psychiatric Institute.

14. Wechsler D, Petermann F (2017): *WISC-V - Wechsler Intelligence Scale for Children – (German version) Fifth Edition |*. Testzentrale. Retrieved April 1, 2024, from https://www.testzentrale.ch/shop/wechsler-intelligence-scale-for-children-fifth-edition.html

15. Wechsler D, Petermann F (2012): *WAIS-IV - Wechsler Adult Intelligence Scale*. Testzentrale. Retrieved April 1, 2024, from https://www.testzentrale.ch/shop/wechsler-adult-intelligence-scale-fourth-edition.html

16. Hyatt CS, Owens MM, Crowe ML, Carter NT, Lynam DR, Miller JD (2020): The quandary of covarying: A brief review and empirical examination of covariate use in structural neuroimaging studies on psychological variables. *NeuroImage* 205: 116225.

17. Giedd JN (2004): Structural Magnetic Resonance Imaging of the Adolescent Brain. *Ann N Y Acad Sci* 1021: 77–85.

18. Luciana M (2013): Adolescent brain development in normality and psychopathology. *Dev Psychopathol* 25: 1325.

19. Meyer HC, Lee FS (2019): Translating Developmental Neuroscience to Understand Risk for Psychiatric Disorders. *Am J Psychiatry* 176: 179–185.

20. Blakemore S-J (2012): Imaging brain development: The adolescent brain. *NeuroImage* 61: 397–406.

21. Konrad K, Firk C, Uhlhaas PJ (2013): Brain Development During Adolescence: Neuroscientific Insights Into This Developmental Period. *Dtsch Ärztebl Int* 110: 425.
